# Supplementary material for: Discovering Common miRNA Signatures Underlying Female-Specific Cancers via a Machine Learning Approach Driven by the Cancer Hallmark ERBB
Source: Biomedicines. 2022 Jun 2;10(6):1306. doi: 10.3390/biomedicines10061306 (PMC9219956; doi:10.3390/biomedicines10061306)
Supplement: Supplementary file 1 [file biomedicines-10-01306-s001.zip › biomedicines-1705497-supplementary.pdf]

# Supplementary Materials

## Figure S1

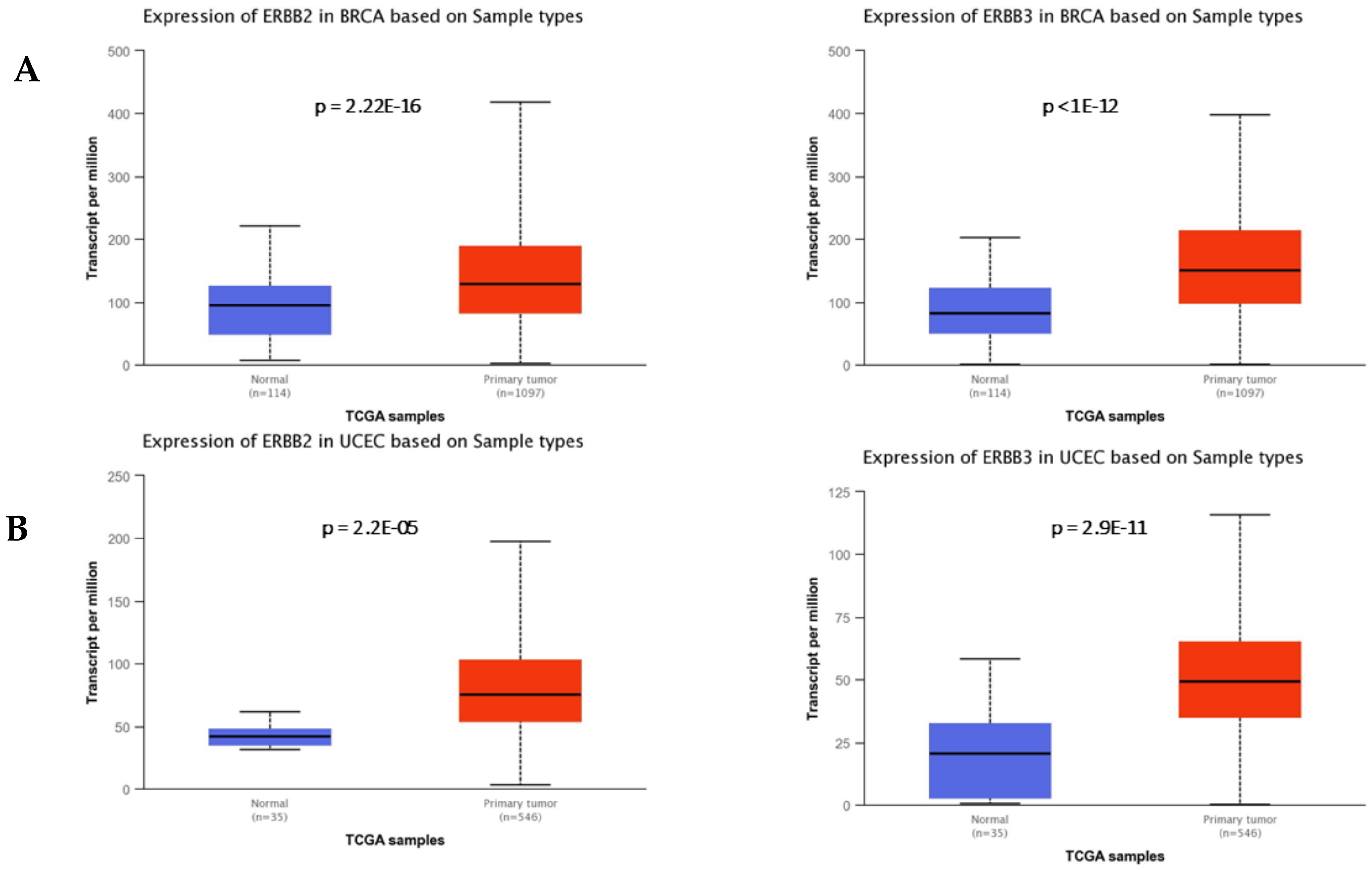


**Figure S1. Expression profile of ERBB2 and ERBB3 in breast cancer (TCGA-BRCA) and uterine corpus endometrial carcinoma (TCGA-UCEC), and normal samples (RNA-seq, transcript per million).** A) ERBB2 and ERBB3 expression in TCGA-BRCA dataset (on the left and on the right, respectively) by comparing N. 114 solid tissue normal vs N. 1091 breast invasive carcinoma. B) ERBB2 and ERBB3 expression in TCGA-UCEC dataset (on the left and on the right , respectively) by comparing N. 35 solid tissue normal vs N. 546 primary tumor samples. Statistical significance in each box plot (normal vs primary) two sample T-test <<0.01.

# Figure S2

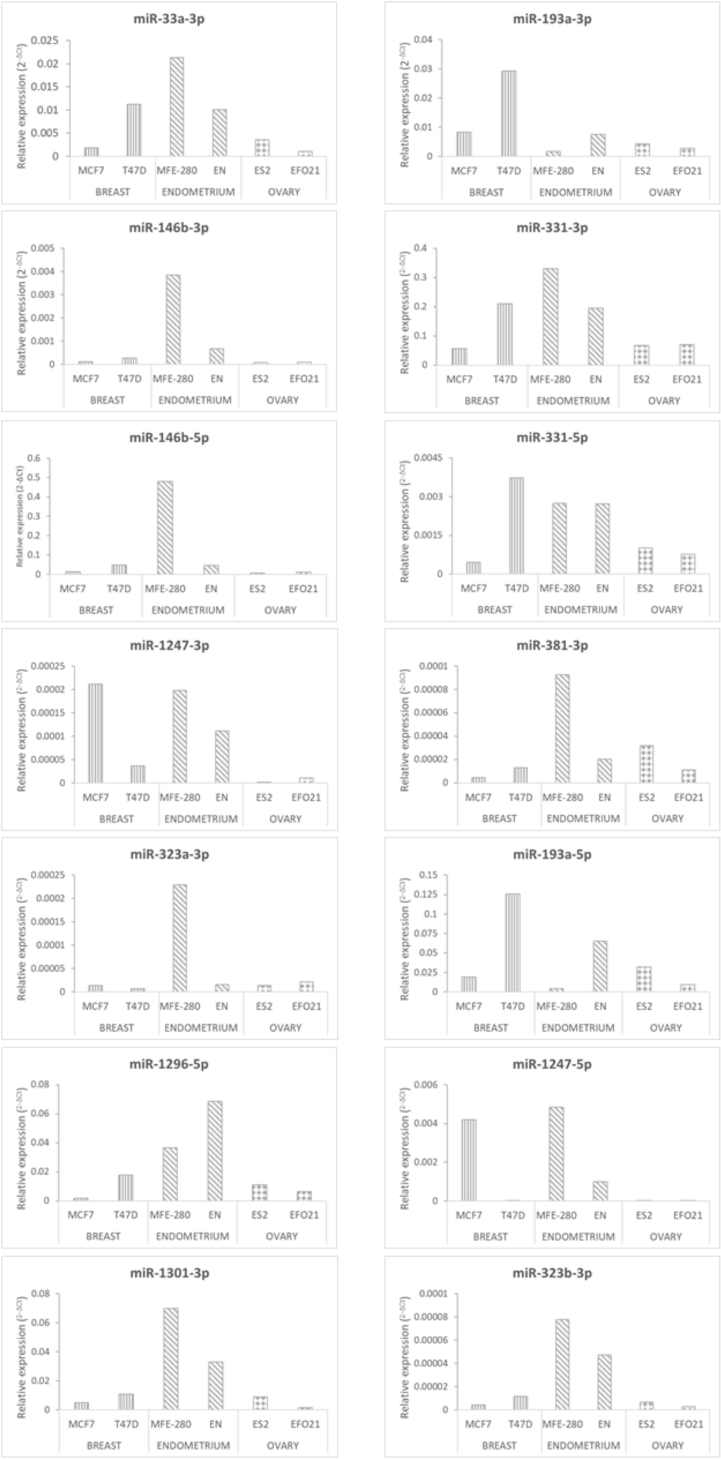


**Figure S2. Relative expression of the 14 selected miRNAs in cancer cell lines.** Each graph shows the expression level of miRNAs in all evaluated cancer cell lines (breast: MCF7, T47D; endometrium: MFE-280, EN; and ovary: ES2, EFO21). The relative expression of miRNAs is expressed here as 2-ΔCt.

**Table S1:** TCGA dataset barcodes

| Project | Sample_Type | barcode | TCGA_patient_barcode |
|---|---|---|---|
| TCGA-OV | Primary Tumor | TCGA-04-1331-01A-01R-1569-13 | TCGA-04-1331 |
| TCGA-OV | Primary Tumor | TCGA-04-1332-01A-01R-1564-13 | TCGA-04-1332 |
| TCGA-OV | Primary Tumor | TCGA-04-1336-01A-01R-1564-13 | TCGA-04-1336 |
| TCGA-OV | Primary Tumor | TCGA-04-1337-01A-01R-1564-13 | TCGA-04-1337 |
| TCGA-OV | Primary Tumor | TCGA-04-1341-01A-01R-1564-13 | TCGA-04-1341 |
| TCGA-OV | Primary Tumor | TCGA-04-1342-01A-01R-1564-13 | TCGA-04-1342 |
| TCGA-OV | Primary Tumor | TCGA-04-1343-01A-01R-1564-13 | TCGA-04-1343 |
| TCGA-OV | Primary Tumor | TCGA-04-1346-01A-01R-1569-13 | TCGA-04-1346 |
| TCGA-OV | Primary Tumor | TCGA-04-1347-01A-01R-1564-13 | TCGA-04-1347 |
| TCGA-OV | Primary Tumor | TCGA-04-1348-01A-01R-1565-13 | TCGA-04-1348 |
| TCGA-OV | Primary Tumor | TCGA-04-1349-01A-01R-1565-13 | TCGA-04-1349 |
| TCGA-OV | Primary Tumor | TCGA-04-1350-01A-01R-1565-13 | TCGA-04-1350 |
| TCGA-OV | Primary Tumor | TCGA-04-1356-01A-01R-1569-13 | TCGA-04-1356 |
| TCGA-OV | Primary Tumor | TCGA-04-1357-01A-01R-1565-13 | TCGA-04-1357 |
| TCGA-OV | Primary Tumor | TCGA-04-1361-01A-01R-1565-13 | TCGA-04-1361 |
| TCGA-OV | Primary Tumor | TCGA-04-1362-01A-01R-1565-13 | TCGA-04-1362 |
| TCGA-OV | Primary Tumor | TCGA-04-1364-01A-01R-1565-13 | TCGA-04-1364 |
| TCGA-OV | Primary Tumor | TCGA-04-1365-01A-01R-1565-13 | TCGA-04-1365 |
| TCGA-OV | Primary Tumor | TCGA-04-1367-01A-01R-1565-13 | TCGA-04-1367 |
| TCGA-OV | Primary Tumor | TCGA-04-1514-01A-01R-1566-13 | TCGA-04-1514 |
| TCGA-OV | Primary Tumor | TCGA-04-1517-01A-01R-1565-13 | TCGA-04-1517 |
| TCGA-OV | Primary Tumor | TCGA-04-1519-01A-01R-1565-13 | TCGA-04-1519 |
| TCGA-OV | Primary Tumor | TCGA-04-1525-01A-01R-1565-13 | TCGA-04-1525 |
| TCGA-OV | Primary Tumor | TCGA-04-1530-01A-02R-1569-13 | TCGA-04-1530 |
| TCGA-OV | Primary Tumor | TCGA-04-1536-01A-01R-1566-13 | TCGA-04-1536 |
| TCGA-OV | Primary Tumor | TCGA-04-1542-01A-01R-1566-13 | TCGA-04-1542 |
| TCGA-OV | Primary Tumor | TCGA-04-1638-01A-01R-1567-13 | TCGA-04-1638 |
| TCGA-OV | Primary Tumor | TCGA-04-1646-01A-01R-1567-13 | TCGA-04-1646 |
| TCGA-OV | Primary Tumor | TCGA-04-1648-01A-01R-1567-13 | TCGA-04-1648 |
| TCGA-OV | Primary Tumor | TCGA-04-1649-01A-01R-1567-13 | TCGA-04-1649 |
| TCGA-OV | Primary Tumor | TCGA-04-1651-01A-01R-1567-13 | TCGA-04-1651 |
| TCGA-OV | Primary Tumor | TCGA-04-1652-01A-01R-1567-13 | TCGA-04-1652 |
| TCGA-OV | Primary Tumor | TCGA-04-1654-01A-02R-1567-13 | TCGA-04-1654 |
| TCGA-OV | Primary Tumor | TCGA-04-1655-01A-01R-1566-13 | TCGA-04-1655 |
| TCGA-OV | Primary Tumor | TCGA-09-0364-01A-02R-1564-13 | TCGA-09-0364 |
| TCGA-OV | Primary Tumor | TCGA-09-0366-01A-01R-1564-13 | TCGA-09-0366 |
| TCGA-OV | Primary Tumor | TCGA-09-0366-01A-01R-1986-13 | TCGA-09-0366 |
| TCGA-OV | Primary Tumor | TCGA-09-0367-01A-01R-1564-13 | TCGA-09-0367 |
| TCGA-OV | Primary Tumor | TCGA-09-1661-01B-01R-1566-13 | TCGA-09-1661 |
| TCGA-OV | Primary Tumor | TCGA-09-1662-01A-01R-1566-13 | TCGA-09-1662 |
| TCGA-OV | Primary Tumor | TCGA-09-1665-01B-01R-1566-13 | TCGA-09-1665 |
| TCGA-OV | Primary Tumor | TCGA-09-1666-01A-01R-1566-13 | TCGA-09-1666 |
| TCGA-OV | Primary Tumor | TCGA-09-1667-01C-01R-1566-13 | TCGA-09-1667 |
| TCGA-OV | Primary Tumor | TCGA-09-1668-01B-01R-1566-13 | TCGA-09-1668 |
| TCGA-OV | Primary Tumor | TCGA-09-1669-01A-01R-1566-13 | TCGA-09-1669 |
| TCGA-OV | Primary Tumor | TCGA-09-1670-01A-01R-1566-13 | TCGA-09-1670 |
| TCGA-OV | Primary Tumor | TCGA-09-1673-01A-01R-1566-13 | TCGA-09-1673 |
| TCGA-OV | Primary Tumor | TCGA-09-1674-01A-01R-1566-13 | TCGA-09-1674 |
| TCGA-OV | Primary Tumor | TCGA-09-2044-01B-01R-1568-13 | TCGA-09-2044 |
| TCGA-OV | Primary Tumor | TCGA-09-2045-01A-01R-1568-13 | TCGA-09-2045 |
| TCGA-OV | Primary Tumor | TCGA-09-2048-01A-01R-1568-13 | TCGA-09-2048 |
| TCGA-OV | Primary Tumor | TCGA-09-2050-01A-01R-1568-13 | TCGA-09-2050 |
| TCGA-OV | Primary Tumor | TCGA-09-2051-01A-01R-1568-13 | TCGA-09-2051 |
| TCGA-OV | Primary Tumor | TCGA-09-2053-01C-01R-1568-13 | TCGA-09-2053 |
| TCGA-OV | Primary Tumor | TCGA-09-2054-01A-01R-1568-13 | TCGA-09-2054 |
| TCGA-OV | Primary Tumor | TCGA-09-2056-01B-01R-1568-13 | TCGA-09-2056 |
| TCGA-OV | Primary Tumor | TCGA-10-0926-01A-01R-1564-13 | TCGA-10-0926 |

| | | | |
|---|---|---|---|
| TCGA-OV | Primary Tumor | TCGA-10-0927-01A-02R-1564-13 | TCGA-10-0927 |
| TCGA-OV | Primary Tumor | TCGA-10-0928-01A-02R-1564-13 | TCGA-10-0928 |
| TCGA-OV | Primary Tumor | TCGA-10-0930-01A-02R-1564-13 | TCGA-10-0930 |
| TCGA-OV | Primary Tumor | TCGA-10-0931-01A-01R-1564-13 | TCGA-10-0931 |
| TCGA-OV | Primary Tumor | TCGA-10-0933-01A-01R-1569-13 | TCGA-10-0933 |
| TCGA-OV | Primary Tumor | TCGA-10-0934-01A-02R-1564-13 | TCGA-10-0934 |
| TCGA-OV | Primary Tumor | TCGA-10-0936-01A-01R-1564-13 | TCGA-10-0936 |
| TCGA-OV | Primary Tumor | TCGA-10-0937-01A-02R-1564-13 | TCGA-10-0937 |
| TCGA-OV | Primary Tumor | TCGA-10-0938-01A-02R-1564-13 | TCGA-10-0938 |
| TCGA-OV | Primary Tumor | TCGA-13-0714-01A-01R-1564-13 | TCGA-13-0714 |
| TCGA-OV | Primary Tumor | TCGA-13-0720-01A-01R-1564-13 | TCGA-13-0720 |
| TCGA-OV | Primary Tumor | TCGA-13-0723-01A-02R-1564-13 | TCGA-13-0723 |
| TCGA-OV | Primary Tumor | TCGA-13-0724-01A-01R-1564-13 | TCGA-13-0724 |
| TCGA-OV | Primary Tumor | TCGA-13-0725-01A-01R-1986-13 | TCGA-13-0725 |
| TCGA-OV | Primary Tumor | TCGA-13-0726-01A-01R-1564-13 | TCGA-13-0726 |
| TCGA-OV | Primary Tumor | TCGA-13-0727-01A-01R-1564-13 | TCGA-13-0727 |
| TCGA-OV | Primary Tumor | TCGA-13-0730-01A-01R-1564-13 | TCGA-13-0730 |
| TCGA-OV | Primary Tumor | TCGA-13-0751-01A-01R-1564-13 | TCGA-13-0751 |
| TCGA-OV | Primary Tumor | TCGA-13-0755-01A-01R-1564-13 | TCGA-13-0755 |
| TCGA-OV | Primary Tumor | TCGA-13-0757-01A-01R-1564-13 | TCGA-13-0757 |
| TCGA-OV | Primary Tumor | TCGA-13-0760-01A-01R-1564-13 | TCGA-13-0760 |
| TCGA-OV | Primary Tumor | TCGA-13-0761-01A-01R-1564-13 | TCGA-13-0761 |
| TCGA-OV | Primary Tumor | TCGA-13-0762-01A-01R-1564-13 | TCGA-13-0762 |
| TCGA-OV | Primary Tumor | TCGA-13-0765-01A-01R-1564-13 | TCGA-13-0765 |
| TCGA-OV | Primary Tumor | TCGA-13-0766-01A-02R-1564-13 | TCGA-13-0766 |
| TCGA-OV | Primary Tumor | TCGA-13-0768-01A-01R-1569-13 | TCGA-13-0768 |
| TCGA-OV | Primary Tumor | TCGA-13-0791-01A-01R-1564-13 | TCGA-13-0791 |
| TCGA-OV | Primary Tumor | TCGA-13-0792-01A-01R-1564-13 | TCGA-13-0792 |
| TCGA-OV | Primary Tumor | TCGA-13-0793-01A-01R-1564-13 | TCGA-13-0793 |
| TCGA-OV | Primary Tumor | TCGA-13-0794-01A-01R-1564-13 | TCGA-13-0794 |
| TCGA-OV | Primary Tumor | TCGA-13-0795-01A-01R-1564-13 | TCGA-13-0795 |
| TCGA-OV | Primary Tumor | TCGA-13-0797-01A-01R-1564-13 | TCGA-13-0797 |
| TCGA-OV | Primary Tumor | TCGA-13-0799-01A-01R-1564-13 | TCGA-13-0799 |
| TCGA-OV | Primary Tumor | TCGA-13-0800-01A-01R-1564-13 | TCGA-13-0800 |
| TCGA-OV | Primary Tumor | TCGA-13-0801-01A-01R-1564-13 | TCGA-13-0801 |
| TCGA-OV | Primary Tumor | TCGA-13-0802-01A-01R-1564-13 | TCGA-13-0802 |
| TCGA-OV | Primary Tumor | TCGA-13-0803-01A-01R-1564-13 | TCGA-13-0803 |
| TCGA-OV | Primary Tumor | TCGA-13-0804-01A-01R-1564-13 | TCGA-13-0804 |
| TCGA-OV | Primary Tumor | TCGA-13-0805-01A-01R-1564-13 | TCGA-13-0805 |
| TCGA-OV | Primary Tumor | TCGA-13-0883-01A-02R-1569-13 | TCGA-13-0883 |
| TCGA-OV | Primary Tumor | TCGA-13-0884-01B-01R-1565-13 | TCGA-13-0884 |
| TCGA-OV | Primary Tumor | TCGA-13-0885-01A-02R-1569-13 | TCGA-13-0885 |
| TCGA-OV | Primary Tumor | TCGA-13-0886-01A-01R-1569-13 | TCGA-13-0886 |
| TCGA-OV | Primary Tumor | TCGA-13-0887-01A-01R-1564-13 | TCGA-13-0887 |
| TCGA-OV | Primary Tumor | TCGA-13-0888-01A-01R-1564-13 | TCGA-13-0888 |
| TCGA-OV | Primary Tumor | TCGA-13-0889-01A-01R-1564-13 | TCGA-13-0889 |
| TCGA-OV | Primary Tumor | TCGA-13-0890-01A-01R-1564-13 | TCGA-13-0890 |
| TCGA-OV | Primary Tumor | TCGA-13-0891-01A-01R-1564-13 | TCGA-13-0891 |
| TCGA-OV | Primary Tumor | TCGA-13-0893-01B-01R-1565-13 | TCGA-13-0893 |
| TCGA-OV | Primary Tumor | TCGA-13-0894-01B-01R-1565-13 | TCGA-13-0894 |
| TCGA-OV | Primary Tumor | TCGA-13-0897-01A-01R-1564-13 | TCGA-13-0897 |
| TCGA-OV | Primary Tumor | TCGA-13-0899-01A-01R-1564-13 | TCGA-13-0899 |
| TCGA-OV | Primary Tumor | TCGA-13-0900-01B-01R-1565-13 | TCGA-13-0900 |
| TCGA-OV | Primary Tumor | TCGA-13-0901-01B-01R-1565-13 | TCGA-13-0901 |
| TCGA-OV | Primary Tumor | TCGA-13-0903-01A-01R-1564-13 | TCGA-13-0903 |
| TCGA-OV | Primary Tumor | TCGA-13-0904-01A-02R-1564-13 | TCGA-13-0904 |
| TCGA-OV | Primary Tumor | TCGA-13-0905-01B-01R-1565-13 | TCGA-13-0905 |
| TCGA-OV | Primary Tumor | TCGA-13-0906-01A-01R-1564-13 | TCGA-13-0906 |
| TCGA-OV | Primary Tumor | TCGA-13-0908-01B-01R-1565-13 | TCGA-13-0908 |

| | | | |
|---|---|---|---|
| TCGA-OV | Primary Tumor | TCGA-13-0910-01A-01R-1564-13 | TCGA-13-0910 |
| TCGA-OV | Primary Tumor | TCGA-13-0911-01A-01R-1564-13 | TCGA-13-0911 |
| TCGA-OV | Primary Tumor | TCGA-13-0912-01A-01R-1564-13 | TCGA-13-0912 |
| TCGA-OV | Primary Tumor | TCGA-13-0913-01A-01R-1564-13 | TCGA-13-0913 |
| TCGA-OV | Primary Tumor | TCGA-13-0916-01A-01R-1564-13 | TCGA-13-0916 |
| TCGA-OV | Primary Tumor | TCGA-13-0919-01A-01R-1564-13 | TCGA-13-0919 |
| TCGA-OV | Primary Tumor | TCGA-13-0920-01A-01R-1564-13 | TCGA-13-0920 |
| TCGA-OV | Primary Tumor | TCGA-13-0921-01A-01R-1564-13 | TCGA-13-0921 |
| TCGA-OV | Primary Tumor | TCGA-13-0923-01A-01R-1564-13 | TCGA-13-0923 |
| TCGA-OV | Primary Tumor | TCGA-13-0924-01A-01R-1564-13 | TCGA-13-0924 |
| TCGA-OV | Primary Tumor | TCGA-13-1403-01A-01R-1565-13 | TCGA-13-1403 |
| TCGA-OV | Primary Tumor | TCGA-13-1404-01A-01R-1565-13 | TCGA-13-1404 |
| TCGA-OV | Primary Tumor | TCGA-13-1405-01A-01R-1565-13 | TCGA-13-1405 |
| TCGA-OV | Primary Tumor | TCGA-13-1407-01A-01R-1565-13 | TCGA-13-1407 |
| TCGA-OV | Primary Tumor | TCGA-13-1408-01A-01R-1565-13 | TCGA-13-1408 |
| TCGA-OV | Primary Tumor | TCGA-13-1409-01A-01R-1565-13 | TCGA-13-1409 |
| TCGA-OV | Primary Tumor | TCGA-13-1410-01A-01R-1565-13 | TCGA-13-1410 |
| TCGA-OV | Primary Tumor | TCGA-13-1411-01A-01R-1565-13 | TCGA-13-1411 |
| TCGA-OV | Primary Tumor | TCGA-13-1412-01A-01R-1565-13 | TCGA-13-1412 |
| TCGA-OV | Primary Tumor | TCGA-13-1477-01A-01R-1565-13 | TCGA-13-1477 |
| TCGA-OV | Primary Tumor | TCGA-13-1481-01A-01R-1565-13 | TCGA-13-1481 |
| TCGA-OV | Primary Tumor | TCGA-13-1482-01A-01R-1565-13 | TCGA-13-1482 |
| TCGA-OV | Primary Tumor | TCGA-13-1483-01A-01R-1565-13 | TCGA-13-1483 |
| TCGA-OV | Primary Tumor | TCGA-13-1484-01A-01R-1565-13 | TCGA-13-1484 |
| TCGA-OV | Primary Tumor | TCGA-13-1485-01A-02R-1565-13 | TCGA-13-1485 |
| TCGA-OV | Primary Tumor | TCGA-13-1487-01A-01R-1565-13 | TCGA-13-1487 |
| TCGA-OV | Primary Tumor | TCGA-13-1488-01A-01R-1565-13 | TCGA-13-1488 |
| TCGA-OV | Primary Tumor | TCGA-13-1489-01A-01R-1565-13 | TCGA-13-1489 |
| TCGA-OV | Primary Tumor | TCGA-13-1491-01A-01R-1565-13 | TCGA-13-1491 |
| TCGA-OV | Primary Tumor | TCGA-13-1492-01A-01R-1565-13 | TCGA-13-1492 |
| TCGA-OV | Primary Tumor | TCGA-13-1494-01A-01R-1565-13 | TCGA-13-1494 |
| TCGA-OV | Primary Tumor | TCGA-13-1495-01A-01R-1565-13 | TCGA-13-1495 |
| TCGA-OV | Primary Tumor | TCGA-13-1496-01A-01R-1565-13 | TCGA-13-1496 |
| TCGA-OV | Primary Tumor | TCGA-13-1497-01A-01R-1565-13 | TCGA-13-1497 |
| TCGA-OV | Primary Tumor | TCGA-13-1498-01A-01R-1565-13 | TCGA-13-1498 |
| TCGA-OV | Primary Tumor | TCGA-13-1499-01A-01R-1565-13 | TCGA-13-1499 |
| TCGA-OV | Primary Tumor | TCGA-13-1500-01A-01R-1565-13 | TCGA-13-1500 |
| TCGA-OV | Primary Tumor | TCGA-13-1501-01A-01R-1565-13 | TCGA-13-1501 |
| TCGA-OV | Primary Tumor | TCGA-13-1504-01A-01R-1565-13 | TCGA-13-1504 |
| TCGA-OV | Primary Tumor | TCGA-13-1505-01A-01R-1565-13 | TCGA-13-1505 |
| TCGA-OV | Primary Tumor | TCGA-13-1506-01A-01R-1565-13 | TCGA-13-1506 |
| TCGA-OV | Primary Tumor | TCGA-13-1507-01A-01R-1565-13 | TCGA-13-1507 |
| TCGA-OV | Primary Tumor | TCGA-13-1509-01A-01R-1565-13 | TCGA-13-1509 |
| TCGA-OV | Primary Tumor | TCGA-13-1510-01A-02R-1565-13 | TCGA-13-1510 |
| TCGA-OV | Primary Tumor | TCGA-13-1511-01A-01R-1565-13 | TCGA-13-1511 |
| TCGA-OV | Primary Tumor | TCGA-13-1512-01A-01R-1565-13 | TCGA-13-1512 |
| TCGA-OV | Primary Tumor | TCGA-13-2060-01A-01R-1568-13 | TCGA-13-2060 |
| TCGA-OV | Primary Tumor | TCGA-13-A5FT-01A-11R-A407-13 | TCGA-13-A5FT |
| TCGA-OV | Primary Tumor | TCGA-13-A5FU-01A-11R-A407-13 | TCGA-13-A5FU |
| TCGA-OV | Primary Tumor | TCGA-20-0987-01A-02R-1564-13 | TCGA-20-0987 |
| TCGA-OV | Primary Tumor | TCGA-20-0990-01A-01R-1564-13 | TCGA-20-0990 |
| TCGA-OV | Primary Tumor | TCGA-20-0991-01A-01R-1564-13 | TCGA-20-0991 |
| TCGA-OV | Primary Tumor | TCGA-20-1682-01A-01R-1564-13 | TCGA-20-1682 |
| TCGA-OV | Primary Tumor | TCGA-20-1683-01A-01R-1566-13 | TCGA-20-1683 |
| TCGA-OV | Primary Tumor | TCGA-20-1684-01A-01R-1566-13 | TCGA-20-1684 |
| TCGA-OV | Primary Tumor | TCGA-20-1685-01A-01R-1566-13 | TCGA-20-1685 |
| TCGA-OV | Primary Tumor | TCGA-20-1686-01A-01R-1566-13 | TCGA-20-1686 |
| TCGA-OV | Primary Tumor | TCGA-20-1687-01A-01R-1566-13 | TCGA-20-1687 |
| TCGA-OV | Primary Tumor | TCGA-23-1021-01B-01R-1564-13 | TCGA-23-1021 |

| | | | |
|---|---|---|---|
| TCGA-OV | Primary Tumor | TCGA-23-1022-01A-01R-1564-13 | TCGA-23-1022 |
| TCGA-OV | Primary Tumor | TCGA-23-1023-01A-02R-1564-13 | TCGA-23-1023 |
| TCGA-OV | Primary Tumor | TCGA-23-1024-01A-02R-1564-13 | TCGA-23-1024 |
| TCGA-OV | Primary Tumor | TCGA-23-1026-01B-01R-1569-13 | TCGA-23-1026 |
| TCGA-OV | Primary Tumor | TCGA-23-1027-01A-02R-1564-13 | TCGA-23-1027 |
| TCGA-OV | Primary Tumor | TCGA-23-1028-01A-01R-1564-13 | TCGA-23-1028 |
| TCGA-OV | Primary Tumor | TCGA-23-1029-01B-01R-1567-13 | TCGA-23-1029 |
| TCGA-OV | Primary Tumor | TCGA-23-1030-01A-02R-1564-13 | TCGA-23-1030 |
| TCGA-OV | Primary Tumor | TCGA-23-1032-01A-02R-1564-13 | TCGA-23-1032 |
| TCGA-OV | Primary Tumor | TCGA-23-1107-01A-01R-1564-13 | TCGA-23-1107 |
| TCGA-OV | Primary Tumor | TCGA-23-1109-01A-01R-1564-13 | TCGA-23-1109 |
| TCGA-OV | Primary Tumor | TCGA-23-1110-01A-01R-1564-13 | TCGA-23-1110 |
| TCGA-OV | Primary Tumor | TCGA-23-1111-01A-01R-1567-13 | TCGA-23-1111 |
| TCGA-OV | Primary Tumor | TCGA-23-1113-01A-01R-1564-13 | TCGA-23-1113 |
| TCGA-OV | Primary Tumor | TCGA-23-1114-01B-01R-1566-13 | TCGA-23-1114 |
| TCGA-OV | Primary Tumor | TCGA-23-1116-01A-01R-1564-13 | TCGA-23-1116 |
| TCGA-OV | Primary Tumor | TCGA-23-1118-01A-01R-1564-13 | TCGA-23-1118 |
| TCGA-OV | Primary Tumor | TCGA-23-1119-01A-02R-1565-13 | TCGA-23-1119 |
| TCGA-OV | Primary Tumor | TCGA-23-1120-01A-02R-1565-13 | TCGA-23-1120 |
| TCGA-OV | Primary Tumor | TCGA-23-1121-01A-01R-1569-13 | TCGA-23-1121 |
| TCGA-OV | Primary Tumor | TCGA-23-1122-01A-01R-1565-13 | TCGA-23-1122 |
| TCGA-OV | Primary Tumor | TCGA-23-1123-01A-01R-1565-13 | TCGA-23-1123 |
| TCGA-OV | Primary Tumor | TCGA-23-1124-01A-01R-1565-13 | TCGA-23-1124 |
| TCGA-OV | Primary Tumor | TCGA-23-1809-01A-01R-1566-13 | TCGA-23-1809 |
| TCGA-OV | Primary Tumor | TCGA-23-2072-01A-01R-1568-13 | TCGA-23-2072 |
| TCGA-OV | Primary Tumor | TCGA-23-2077-01A-01R-1568-13 | TCGA-23-2077 |
| TCGA-OV | Primary Tumor | TCGA-23-2078-01A-01R-1568-13 | TCGA-23-2078 |
| TCGA-OV | Primary Tumor | TCGA-23-2079-01A-01R-1568-13 | TCGA-23-2079 |
| TCGA-OV | Primary Tumor | TCGA-23-2081-01A-01R-1568-13 | TCGA-23-2081 |
| TCGA-OV | Primary Tumor | TCGA-23-2084-01A-02R-1568-13 | TCGA-23-2084 |
| TCGA-OV | Primary Tumor | TCGA-24-0966-01A-01R-1564-13 | TCGA-24-0966 |
| TCGA-OV | Primary Tumor | TCGA-24-0968-01A-01R-1569-13 | TCGA-24-0968 |
| TCGA-OV | Primary Tumor | TCGA-24-0970-01B-01R-1565-13 | TCGA-24-0970 |
| TCGA-OV | Primary Tumor | TCGA-24-0975-01A-02R-1565-13 | TCGA-24-0975 |
| TCGA-OV | Primary Tumor | TCGA-24-0979-01A-01R-1565-13 | TCGA-24-0979 |
| TCGA-OV | Primary Tumor | TCGA-24-0982-01A-01R-1565-13 | TCGA-24-0982 |
| TCGA-OV | Primary Tumor | TCGA-24-1103-01A-01R-1565-13 | TCGA-24-1103 |
| TCGA-OV | Primary Tumor | TCGA-24-1104-01A-01R-1565-13 | TCGA-24-1104 |
| TCGA-OV | Primary Tumor | TCGA-24-1105-01A-01R-1565-13 | TCGA-24-1105 |
| TCGA-OV | Primary Tumor | TCGA-24-1413-01A-01R-1565-13 | TCGA-24-1413 |
| TCGA-OV | Primary Tumor | TCGA-24-1416-01A-01R-1565-13 | TCGA-24-1416 |
| TCGA-OV | Primary Tumor | TCGA-24-1417-01A-01R-1565-13 | TCGA-24-1417 |
| TCGA-OV | Primary Tumor | TCGA-24-1418-01A-01R-1565-13 | TCGA-24-1418 |
| TCGA-OV | Primary Tumor | TCGA-24-1419-01A-01R-1565-13 | TCGA-24-1419 |
| TCGA-OV | Primary Tumor | TCGA-24-1422-01A-01R-1565-13 | TCGA-24-1422 |
| TCGA-OV | Primary Tumor | TCGA-24-1423-01A-01R-1565-13 | TCGA-24-1423 |
| TCGA-OV | Primary Tumor | TCGA-24-1424-01A-01R-1565-13 | TCGA-24-1424 |
| TCGA-OV | Primary Tumor | TCGA-24-1425-01A-02R-1566-13 | TCGA-24-1425 |
| TCGA-OV | Primary Tumor | TCGA-24-1426-01A-01R-1565-13 | TCGA-24-1426 |
| TCGA-OV | Primary Tumor | TCGA-24-1427-01A-01R-1565-13 | TCGA-24-1427 |
| TCGA-OV | Primary Tumor | TCGA-24-1428-01A-01R-1564-13 | TCGA-24-1428 |
| TCGA-OV | Primary Tumor | TCGA-24-1430-01A-01R-1566-13 | TCGA-24-1430 |
| TCGA-OV | Primary Tumor | TCGA-24-1431-01A-01R-1566-13 | TCGA-24-1431 |
| TCGA-OV | Primary Tumor | TCGA-24-1434-01A-01R-1566-13 | TCGA-24-1434 |
| TCGA-OV | Primary Tumor | TCGA-24-1435-01A-01R-1566-13 | TCGA-24-1435 |
| TCGA-OV | Primary Tumor | TCGA-24-1436-01A-01R-1566-13 | TCGA-24-1436 |
| TCGA-OV | Primary Tumor | TCGA-24-1463-01A-01R-1566-13 | TCGA-24-1463 |
| TCGA-OV | Primary Tumor | TCGA-24-1464-01A-01R-1566-13 | TCGA-24-1464 |
| TCGA-OV | Primary Tumor | TCGA-24-1466-01A-01R-1566-13 | TCGA-24-1466 |

| | | | |
|---|---|---|---|
| TCGA-OV | Primary Tumor | TCGA-24-1467-01A-01R-1566-13 | TCGA-24-1467 |
| TCGA-OV | Primary Tumor | TCGA-24-1469-01A-01R-1566-13 | TCGA-24-1469 |
| TCGA-OV | Primary Tumor | TCGA-24-1470-01A-01R-1566-13 | TCGA-24-1470 |
| TCGA-OV | Primary Tumor | TCGA-24-1471-01A-01R-1566-13 | TCGA-24-1471 |
| TCGA-OV | Primary Tumor | TCGA-24-1474-01A-01R-1566-13 | TCGA-24-1474 |
| TCGA-OV | Primary Tumor | TCGA-24-1544-01A-01R-1566-13 | TCGA-24-1544 |
| TCGA-OV | Primary Tumor | TCGA-24-1545-01A-01R-1566-13 | TCGA-24-1545 |
| TCGA-OV | Primary Tumor | TCGA-24-1546-01A-01R-1566-13 | TCGA-24-1546 |
| TCGA-OV | Primary Tumor | TCGA-24-1548-01A-01R-1566-13 | TCGA-24-1548 |
| TCGA-OV | Primary Tumor | TCGA-24-1549-01A-01R-1566-13 | TCGA-24-1549 |
| TCGA-OV | Primary Tumor | TCGA-24-1550-01A-01R-1566-13 | TCGA-24-1550 |
| TCGA-OV | Primary Tumor | TCGA-24-1551-01A-01R-1566-13 | TCGA-24-1551 |
| TCGA-OV | Primary Tumor | TCGA-24-1552-01A-01R-1566-13 | TCGA-24-1552 |
| TCGA-OV | Primary Tumor | TCGA-24-1553-01A-01R-1566-13 | TCGA-24-1553 |
| TCGA-OV | Primary Tumor | TCGA-24-1555-01A-01R-1566-13 | TCGA-24-1555 |
| TCGA-OV | Primary Tumor | TCGA-24-1556-01A-01R-1566-13 | TCGA-24-1556 |
| TCGA-OV | Primary Tumor | TCGA-24-1557-01A-01R-1566-13 | TCGA-24-1557 |
| TCGA-OV | Primary Tumor | TCGA-24-1558-01A-01R-1566-13 | TCGA-24-1558 |
| TCGA-OV | Primary Tumor | TCGA-24-1560-01A-01R-1566-13 | TCGA-24-1560 |
| TCGA-OV | Primary Tumor | TCGA-24-1562-01A-01R-1566-13 | TCGA-24-1562 |
| TCGA-OV | Primary Tumor | TCGA-24-1563-01A-01R-1566-13 | TCGA-24-1563 |
| TCGA-OV | Primary Tumor | TCGA-24-1564-01A-01R-1566-13 | TCGA-24-1564 |
| TCGA-OV | Primary Tumor | TCGA-24-1565-01A-01R-1566-13 | TCGA-24-1565 |
| TCGA-OV | Primary Tumor | TCGA-24-1567-01A-01R-1566-13 | TCGA-24-1567 |
| TCGA-OV | Primary Tumor | TCGA-24-1603-01A-01R-1566-13 | TCGA-24-1603 |
| TCGA-OV | Primary Tumor | TCGA-24-1604-01A-01R-1566-13 | TCGA-24-1604 |
| TCGA-OV | Primary Tumor | TCGA-24-1614-01A-01R-1566-13 | TCGA-24-1614 |
| TCGA-OV | Primary Tumor | TCGA-24-1616-01A-01R-1566-13 | TCGA-24-1616 |
| TCGA-OV | Primary Tumor | TCGA-24-1842-01A-01R-1567-13 | TCGA-24-1842 |
| TCGA-OV | Primary Tumor | TCGA-24-1843-01A-01R-1567-13 | TCGA-24-1843 |
| TCGA-OV | Primary Tumor | TCGA-24-1844-01A-01R-1567-13 | TCGA-24-1844 |
| TCGA-OV | Primary Tumor | TCGA-24-1845-01A-01R-1567-13 | TCGA-24-1845 |
| TCGA-OV | Primary Tumor | TCGA-24-1846-01A-01R-1567-13 | TCGA-24-1846 |
| TCGA-OV | Primary Tumor | TCGA-24-1847-01A-01R-1566-13 | TCGA-24-1847 |
| TCGA-OV | Primary Tumor | TCGA-24-1849-01A-01R-1567-13 | TCGA-24-1849 |
| TCGA-OV | Primary Tumor | TCGA-24-1850-01A-01R-1567-13 | TCGA-24-1850 |
| TCGA-OV | Primary Tumor | TCGA-24-1920-01A-01R-1567-13 | TCGA-24-1920 |
| TCGA-OV | Primary Tumor | TCGA-24-1923-01A-01R-1567-13 | TCGA-24-1923 |
| TCGA-OV | Primary Tumor | TCGA-24-1924-01A-01R-1567-13 | TCGA-24-1924 |
| TCGA-OV | Primary Tumor | TCGA-24-1928-01A-01R-1567-13 | TCGA-24-1928 |
| TCGA-OV | Primary Tumor | TCGA-24-1930-01A-01R-1567-13 | TCGA-24-1930 |
| TCGA-OV | Primary Tumor | TCGA-24-2019-01A-02R-1568-13 | TCGA-24-2019 |
| TCGA-OV | Primary Tumor | TCGA-24-2020-01A-01R-1567-13 | TCGA-24-2020 |
| TCGA-OV | Primary Tumor | TCGA-24-2023-01A-01R-1567-13 | TCGA-24-2023 |
| TCGA-OV | Primary Tumor | TCGA-24-2024-01A-02R-1568-13 | TCGA-24-2024 |
| TCGA-OV | Primary Tumor | TCGA-24-2026-01A-01R-1567-13 | TCGA-24-2026 |
| TCGA-OV | Primary Tumor | TCGA-24-2027-01A-01R-1567-13 | TCGA-24-2027 |
| TCGA-OV | Primary Tumor | TCGA-24-2029-01A-01R-1567-13 | TCGA-24-2029 |
| TCGA-OV | Primary Tumor | TCGA-24-2030-01A-01R-1568-13 | TCGA-24-2030 |
| TCGA-OV | Primary Tumor | TCGA-24-2033-01A-01R-1568-13 | TCGA-24-2033 |
| TCGA-OV | Primary Tumor | TCGA-24-2035-01A-01R-1568-13 | TCGA-24-2035 |
| TCGA-OV | Primary Tumor | TCGA-24-2036-01A-01R-1568-13 | TCGA-24-2036 |
| TCGA-OV | Primary Tumor | TCGA-24-2038-01A-01R-1568-13 | TCGA-24-2038 |
| TCGA-OV | Primary Tumor | TCGA-24-2254-01A-01R-1568-13 | TCGA-24-2254 |
| TCGA-OV | Primary Tumor | TCGA-24-2260-01A-01R-1568-13 | TCGA-24-2260 |
| TCGA-OV | Primary Tumor | TCGA-24-2261-01A-01R-1568-13 | TCGA-24-2261 |
| TCGA-OV | Primary Tumor | TCGA-24-2262-01A-01R-1568-13 | TCGA-24-2262 |
| TCGA-OV | Primary Tumor | TCGA-24-2267-01A-01R-1568-13 | TCGA-24-2267 |
| TCGA-OV | Primary Tumor | TCGA-24-2271-01A-01R-1568-13 | TCGA-24-2271 |

| | | | |
|---|---|---|---|
| TCGA-OV | Primary Tumor | TCGA-24-2280-01A-01R-1568-13 | TCGA-24-2280 |
| TCGA-OV | Primary Tumor | TCGA-24-2281-01A-01R-1568-13 | TCGA-24-2281 |
| TCGA-OV | Primary Tumor | TCGA-24-2288-01A-01R-1568-13 | TCGA-24-2288 |
| TCGA-OV | Primary Tumor | TCGA-24-2289-01A-01R-1568-13 | TCGA-24-2289 |
| TCGA-OV | Primary Tumor | TCGA-24-2290-01A-01R-1568-13 | TCGA-24-2290 |
| TCGA-OV | Primary Tumor | TCGA-24-2293-01A-01R-1568-13 | TCGA-24-2293 |
| TCGA-OV | Primary Tumor | TCGA-24-2297-01A-01R-1568-13 | TCGA-24-2297 |
| TCGA-OV | Primary Tumor | TCGA-24-2298-01A-01R-1569-13 | TCGA-24-2298 |
| TCGA-OV | Primary Tumor | TCGA-25-1312-01A-01R-1565-13 | TCGA-25-1312 |
| TCGA-OV | Primary Tumor | TCGA-25-1313-01A-01R-1565-13 | TCGA-25-1313 |
| TCGA-OV | Primary Tumor | TCGA-25-1314-01A-01R-1565-13 | TCGA-25-1314 |
| TCGA-OV | Primary Tumor | TCGA-25-1315-01A-01R-1565-13 | TCGA-25-1315 |
| TCGA-OV | Primary Tumor | TCGA-25-1316-01A-01R-1565-13 | TCGA-25-1316 |
| TCGA-OV | Primary Tumor | TCGA-25-1317-01A-01R-1565-13 | TCGA-25-1317 |
| TCGA-OV | Primary Tumor | TCGA-25-1318-01A-01R-1565-13 | TCGA-25-1318 |
| TCGA-OV | Primary Tumor | TCGA-25-1319-01A-01R-1565-13 | TCGA-25-1319 |
| TCGA-OV | Primary Tumor | TCGA-25-1320-01A-01R-1565-13 | TCGA-25-1320 |
| TCGA-OV | Primary Tumor | TCGA-25-1321-01A-01R-1565-13 | TCGA-25-1321 |
| TCGA-OV | Primary Tumor | TCGA-25-1322-01A-01R-1565-13 | TCGA-25-1322 |
| TCGA-OV | Primary Tumor | TCGA-25-1323-01A-01R-1565-13 | TCGA-25-1323 |
| TCGA-OV | Primary Tumor | TCGA-25-1324-01A-01R-1565-13 | TCGA-25-1324 |
| TCGA-OV | Primary Tumor | TCGA-25-1325-01A-01R-1565-13 | TCGA-25-1325 |
| TCGA-OV | Primary Tumor | TCGA-25-1326-01A-01R-1565-13 | TCGA-25-1326 |
| TCGA-OV | Primary Tumor | TCGA-25-1328-01A-01R-1565-13 | TCGA-25-1328 |
| TCGA-OV | Primary Tumor | TCGA-25-1329-01A-01R-1565-13 | TCGA-25-1329 |
| TCGA-OV | Primary Tumor | TCGA-25-1623-01A-01R-1566-13 | TCGA-25-1623 |
| TCGA-OV | Primary Tumor | TCGA-25-1625-01A-01R-1566-13 | TCGA-25-1625 |
| TCGA-OV | Primary Tumor | TCGA-25-1626-01A-01R-1566-13 | TCGA-25-1626 |
| TCGA-OV | Primary Tumor | TCGA-25-1627-01A-01R-1566-13 | TCGA-25-1627 |
| TCGA-OV | Primary Tumor | TCGA-25-1628-01A-01R-1566-13 | TCGA-25-1628 |
| TCGA-OV | Primary Tumor | TCGA-25-1630-01A-01R-1566-13 | TCGA-25-1630 |
| TCGA-OV | Primary Tumor | TCGA-25-1631-01A-01R-1569-13 | TCGA-25-1631 |
| TCGA-OV | Primary Tumor | TCGA-25-1632-01A-01R-1566-13 | TCGA-25-1632 |
| TCGA-OV | Primary Tumor | TCGA-25-1633-01A-01R-1566-13 | TCGA-25-1633 |
| TCGA-OV | Primary Tumor | TCGA-25-1634-01A-01R-1566-13 | TCGA-25-1634 |
| TCGA-OV | Primary Tumor | TCGA-25-1635-01A-01R-1566-13 | TCGA-25-1635 |
| TCGA-OV | Primary Tumor | TCGA-25-1870-01A-01R-1567-13 | TCGA-25-1870 |
| TCGA-OV | Primary Tumor | TCGA-25-1871-01A-01R-1567-13 | TCGA-25-1871 |
| TCGA-OV | Primary Tumor | TCGA-25-1877-01A-01R-1567-13 | TCGA-25-1877 |
| TCGA-OV | Primary Tumor | TCGA-25-1878-01A-01R-1567-13 | TCGA-25-1878 |
| TCGA-OV | Primary Tumor | TCGA-25-2042-01A-01R-1568-13 | TCGA-25-2042 |
| TCGA-OV | Primary Tumor | TCGA-25-2391-01A-01R-1569-13 | TCGA-25-2391 |
| TCGA-OV | Primary Tumor | TCGA-25-2392-01A-01R-1569-13 | TCGA-25-2392 |
| TCGA-OV | Primary Tumor | TCGA-25-2393-01A-01R-1569-13 | TCGA-25-2393 |
| TCGA-OV | Primary Tumor | TCGA-25-2396-01A-01R-1569-13 | TCGA-25-2396 |
| TCGA-OV | Primary Tumor | TCGA-25-2397-01A-01R-1569-13 | TCGA-25-2397 |
| TCGA-OV | Primary Tumor | TCGA-25-2398-01A-01R-1569-13 | TCGA-25-2398 |
| TCGA-OV | Primary Tumor | TCGA-25-2399-01A-01R-1569-13 | TCGA-25-2399 |
| TCGA-OV | Primary Tumor | TCGA-25-2400-01A-01R-1569-13 | TCGA-25-2400 |
| TCGA-OV | Primary Tumor | TCGA-25-2401-01A-01R-1569-13 | TCGA-25-2401 |
| TCGA-OV | Primary Tumor | TCGA-25-2404-01A-01R-1569-13 | TCGA-25-2404 |
| TCGA-OV | Primary Tumor | TCGA-25-2408-01A-01R-1569-13 | TCGA-25-2408 |
| TCGA-OV | Primary Tumor | TCGA-25-2409-01A-01R-1569-13 | TCGA-25-2409 |
| TCGA-OV | Primary Tumor | TCGA-29-1688-01A-01R-1566-13 | TCGA-29-1688 |
| TCGA-OV | Primary Tumor | TCGA-29-1690-01A-01R-1566-13 | TCGA-29-1690 |
| TCGA-OV | Primary Tumor | TCGA-29-1691-01A-01R-1566-13 | TCGA-29-1691 |
| TCGA-OV | Primary Tumor | TCGA-29-1693-01A-01R-1567-13 | TCGA-29-1693 |
| TCGA-OV | Primary Tumor | TCGA-29-1694-01A-01R-1567-13 | TCGA-29-1694 |
| TCGA-OV | Primary Tumor | TCGA-29-1695-01A-01R-1567-13 | TCGA-29-1695 |

| | | | |
|---|---|---|---|
| TCGA-OV | Primary Tumor | TCGA-29-1696-01A-01R-1567-13 | TCGA-29-1696 |
| TCGA-OV | Primary Tumor | TCGA-29-1697-01A-01R-1567-13 | TCGA-29-1697 |
| TCGA-OV | Primary Tumor | TCGA-29-1698-01A-01R-1567-13 | TCGA-29-1698 |
| TCGA-OV | Primary Tumor | TCGA-29-1699-01A-01R-1567-13 | TCGA-29-1699 |
| TCGA-OV | Primary Tumor | TCGA-29-1701-01A-01R-1567-13 | TCGA-29-1701 |
| TCGA-OV | Primary Tumor | TCGA-29-1702-01A-01R-1567-13 | TCGA-29-1702 |
| TCGA-OV | Primary Tumor | TCGA-29-1703-01A-01R-1567-13 | TCGA-29-1703 |
| TCGA-OV | Primary Tumor | TCGA-29-1705-01A-01R-1567-13 | TCGA-29-1705 |
| TCGA-OV | Primary Tumor | TCGA-29-1707-01A-01R-1567-13 | TCGA-29-1707 |
| TCGA-OV | Primary Tumor | TCGA-29-1710-01A-02R-1567-13 | TCGA-29-1710 |
| TCGA-OV | Primary Tumor | TCGA-29-1711-01A-01R-1567-13 | TCGA-29-1711 |
| TCGA-OV | Primary Tumor | TCGA-29-1761-01A-01R-1567-13 | TCGA-29-1761 |
| TCGA-OV | Primary Tumor | TCGA-29-1762-01A-01R-1567-13 | TCGA-29-1762 |
| TCGA-OV | Primary Tumor | TCGA-29-1763-01A-02R-1567-13 | TCGA-29-1763 |
| TCGA-OV | Primary Tumor | TCGA-29-1764-01A-01R-1567-13 | TCGA-29-1764 |
| TCGA-OV | Primary Tumor | TCGA-29-1766-01A-01R-1567-13 | TCGA-29-1766 |
| TCGA-OV | Primary Tumor | TCGA-29-1768-01A-01R-1567-13 | TCGA-29-1768 |
| TCGA-OV | Primary Tumor | TCGA-29-1769-01A-01R-1567-13 | TCGA-29-1769 |
| TCGA-OV | Primary Tumor | TCGA-29-1770-01A-01R-1567-13 | TCGA-29-1770 |
| TCGA-OV | Primary Tumor | TCGA-29-1771-01A-01R-1567-13 | TCGA-29-1771 |
| TCGA-OV | Primary Tumor | TCGA-29-1774-01A-01R-1567-13 | TCGA-29-1774 |
| TCGA-OV | Primary Tumor | TCGA-29-1776-01A-01R-1567-13 | TCGA-29-1776 |
| TCGA-OV | Primary Tumor | TCGA-29-1777-01A-01R-1567-13 | TCGA-29-1777 |
| TCGA-OV | Primary Tumor | TCGA-29-1778-01A-01R-1567-13 | TCGA-29-1778 |
| TCGA-OV | Primary Tumor | TCGA-29-1781-01A-01R-1567-13 | TCGA-29-1781 |
| TCGA-OV | Primary Tumor | TCGA-29-1783-01A-01R-1567-13 | TCGA-29-1783 |
| TCGA-OV | Primary Tumor | TCGA-29-1784-01A-02R-1567-13 | TCGA-29-1784 |
| TCGA-OV | Primary Tumor | TCGA-29-1785-01A-01R-1567-13 | TCGA-29-1785 |
| TCGA-OV | Primary Tumor | TCGA-29-2414-01A-02R-1569-13 | TCGA-29-2414 |
| TCGA-OV | Primary Tumor | TCGA-29-2425-01A-01R-1569-13 | TCGA-29-2425 |
| TCGA-OV | Primary Tumor | TCGA-29-2427-01A-01R-1569-13 | TCGA-29-2427 |
| TCGA-OV | Primary Tumor | TCGA-29-2428-01A-01R-1569-13 | TCGA-29-2428 |
| TCGA-OV | Primary Tumor | TCGA-29-A5NZ-01A-11R-A407-13 | TCGA-29-A5NZ |
| TCGA-OV | Primary Tumor | TCGA-30-1714-01A-02R-1567-13 | TCGA-30-1714 |
| TCGA-OV | Primary Tumor | TCGA-30-1718-01A-01R-1567-13 | TCGA-30-1718 |
| TCGA-OV | Primary Tumor | TCGA-30-1853-01A-02R-1567-13 | TCGA-30-1853 |
| TCGA-OV | Primary Tumor | TCGA-30-1855-01A-01R-1567-13 | TCGA-30-1855 |
| TCGA-OV | Primary Tumor | TCGA-30-1856-01A-01R-1567-13 | TCGA-30-1856 |
| TCGA-OV | Primary Tumor | TCGA-30-1857-01A-02R-1569-13 | TCGA-30-1857 |
| TCGA-OV | Primary Tumor | TCGA-30-1859-01A-01R-1567-13 | TCGA-30-1859 |
| TCGA-OV | Primary Tumor | TCGA-30-1860-01A-01R-1568-13 | TCGA-30-1860 |
| TCGA-OV | Primary Tumor | TCGA-30-1861-01A-01R-1568-13 | TCGA-30-1861 |
| TCGA-OV | Primary Tumor | TCGA-30-1862-01A-02R-1568-13 | TCGA-30-1862 |
| TCGA-OV | Primary Tumor | TCGA-30-1866-01A-02R-1568-13 | TCGA-30-1866 |
| TCGA-OV | Primary Tumor | TCGA-30-1867-01A-01R-1568-13 | TCGA-30-1867 |
| TCGA-OV | Primary Tumor | TCGA-30-1880-01A-01R-1568-13 | TCGA-30-1880 |
| TCGA-OV | Primary Tumor | TCGA-30-1887-01A-01R-1568-13 | TCGA-30-1887 |
| TCGA-OV | Primary Tumor | TCGA-30-1891-01A-01R-1568-13 | TCGA-30-1891 |
| TCGA-OV | Primary Tumor | TCGA-30-1892-01A-01R-1568-13 | TCGA-30-1892 |
| TCGA-OV | Primary Tumor | TCGA-31-1944-01A-01R-1568-13 | TCGA-31-1944 |
| TCGA-OV | Primary Tumor | TCGA-31-1946-01A-01R-1568-13 | TCGA-31-1946 |
| TCGA-OV | Primary Tumor | TCGA-31-1950-01A-01R-1568-13 | TCGA-31-1950 |
| TCGA-OV | Primary Tumor | TCGA-31-1951-01A-01R-1568-13 | TCGA-31-1951 |
| TCGA-OV | Primary Tumor | TCGA-31-1953-01A-01R-1568-13 | TCGA-31-1953 |
| TCGA-OV | Primary Tumor | TCGA-31-1956-01A-01R-1568-13 | TCGA-31-1956 |
| TCGA-OV | Primary Tumor | TCGA-31-1959-01A-01R-1568-13 | TCGA-31-1959 |
| TCGA-OV | Primary Tumor | TCGA-36-1568-01A-01R-1566-13 | TCGA-36-1568 |
| TCGA-OV | Primary Tumor | TCGA-36-1569-01A-01R-1566-13 | TCGA-36-1569 |
| TCGA-OV | Primary Tumor | TCGA-36-1570-01A-01R-1566-13 | TCGA-36-1570 |

| | | | |
|---|---|---|---|
| TCGA-OV | Primary Tumor | TCGA-36-1571-01A-01R-1566-13 | TCGA-36-1571 |
| TCGA-OV | Primary Tumor | TCGA-36-1574-01A-01R-1566-13 | TCGA-36-1574 |
| TCGA-OV | Primary Tumor | TCGA-36-1575-01A-01R-1566-13 | TCGA-36-1575 |
| TCGA-OV | Primary Tumor | TCGA-36-1576-01A-01R-1566-13 | TCGA-36-1576 |
| TCGA-OV | Primary Tumor | TCGA-36-1577-01A-01R-1566-13 | TCGA-36-1577 |
| TCGA-OV | Primary Tumor | TCGA-36-1578-01A-01R-1566-13 | TCGA-36-1578 |
| TCGA-OV | Primary Tumor | TCGA-36-1580-01A-01R-1566-13 | TCGA-36-1580 |
| TCGA-OV | Primary Tumor | TCGA-36-1581-01A-01R-1566-13 | TCGA-36-1581 |
| TCGA-OV | Primary Tumor | TCGA-3P-A9WA-01A-11R-A407-13 | TCGA-3P-A9WA |
| TCGA-OV | Primary Tumor | TCGA-57-1582-01A-01R-1566-13 | TCGA-57-1582 |
| TCGA-OV | Primary Tumor | TCGA-57-1583-01A-01R-1566-13 | TCGA-57-1583 |
| TCGA-OV | Primary Tumor | TCGA-57-1584-01A-01R-1566-13 | TCGA-57-1584 |
| TCGA-OV | Primary Tumor | TCGA-57-1585-01A-01R-1566-13 | TCGA-57-1585 |
| TCGA-OV | Primary Tumor | TCGA-57-1586-01A-02R-1567-13 | TCGA-57-1586 |
| TCGA-OV | Primary Tumor | TCGA-57-1993-01A-01R-1568-13 | TCGA-57-1993 |
| TCGA-OV | Primary Tumor | TCGA-57-1994-01A-01R-1568-13 | TCGA-57-1994 |
| TCGA-OV | Primary Tumor | TCGA-59-2348-01A-01R-1569-13 | TCGA-59-2348 |
| TCGA-OV | Primary Tumor | TCGA-59-2350-01A-01R-1569-13 | TCGA-59-2350 |
| TCGA-OV | Primary Tumor | TCGA-59-2351-01A-01R-1569-13 | TCGA-59-2351 |
| TCGA-OV | Primary Tumor | TCGA-59-2352-01A-01R-1569-13 | TCGA-59-2352 |
| TCGA-OV | Primary Tumor | TCGA-59-2354-01A-01R-1569-13 | TCGA-59-2354 |
| TCGA-OV | Primary Tumor | TCGA-59-2355-01A-01R-1569-13 | TCGA-59-2355 |
| TCGA-OV | Primary Tumor | TCGA-59-2363-01A-01R-1569-13 | TCGA-59-2363 |
| TCGA-OV | Primary Tumor | TCGA-59-A5PD-01A-11R-A407-13 | TCGA-59-A5PD |
| TCGA-OV | Primary Tumor | TCGA-5X-AA5U-01A-11R-A407-13 | TCGA-5X-AA5U |
| TCGA-OV | Primary Tumor | TCGA-61-1721-01A-01R-1569-13 | TCGA-61-1721 |
| TCGA-OV | Primary Tumor | TCGA-61-1724-01A-01R-1568-13 | TCGA-61-1724 |
| TCGA-OV | Primary Tumor | TCGA-61-1725-01A-01R-1567-13 | TCGA-61-1725 |
| TCGA-OV | Primary Tumor | TCGA-61-1728-01A-01R-1568-13 | TCGA-61-1728 |
| TCGA-OV | Primary Tumor | TCGA-61-1733-01A-01R-1567-13 | TCGA-61-1733 |
| TCGA-OV | Primary Tumor | TCGA-61-1736-01B-01R-1568-13 | TCGA-61-1736 |
| TCGA-OV | Primary Tumor | TCGA-61-1737-01A-01R-1567-13 | TCGA-61-1737 |
| TCGA-OV | Primary Tumor | TCGA-61-1738-01A-01R-1567-13 | TCGA-61-1738 |
| TCGA-OV | Primary Tumor | TCGA-61-1740-01A-01R-1567-13 | TCGA-61-1740 |
| TCGA-OV | Primary Tumor | TCGA-61-1741-01A-02R-1567-13 | TCGA-61-1741 |
| TCGA-OV | Primary Tumor | TCGA-61-1743-01A-01R-1568-13 | TCGA-61-1743 |
| TCGA-OV | Primary Tumor | TCGA-61-1895-01A-01R-1567-13 | TCGA-61-1895 |
| TCGA-OV | Primary Tumor | TCGA-61-1899-01A-01R-1567-13 | TCGA-61-1899 |
| TCGA-OV | Primary Tumor | TCGA-61-1900-01A-01R-1567-13 | TCGA-61-1900 |
| TCGA-OV | Primary Tumor | TCGA-61-1901-01A-01R-1567-13 | TCGA-61-1901 |
| TCGA-OV | Primary Tumor | TCGA-61-1906-01A-01R-1567-13 | TCGA-61-1906 |
| TCGA-OV | Primary Tumor | TCGA-61-1907-01A-01R-1567-13 | TCGA-61-1907 |
| TCGA-OV | Primary Tumor | TCGA-61-1910-01A-01R-1567-13 | TCGA-61-1910 |
| TCGA-OV | Primary Tumor | TCGA-61-1911-01A-01R-1567-13 | TCGA-61-1911 |
| TCGA-OV | Primary Tumor | TCGA-61-1913-01A-01R-1567-13 | TCGA-61-1913 |
| TCGA-OV | Primary Tumor | TCGA-61-1914-01A-01R-1567-13 | TCGA-61-1914 |
| TCGA-OV | Primary Tumor | TCGA-61-1915-01A-01R-1567-13 | TCGA-61-1915 |
| TCGA-OV | Primary Tumor | TCGA-61-1917-01A-01R-1568-13 | TCGA-61-1917 |
| TCGA-OV | Primary Tumor | TCGA-61-1918-01A-01R-1568-13 | TCGA-61-1918 |
| TCGA-OV | Primary Tumor | TCGA-61-1919-01A-01R-1568-13 | TCGA-61-1919 |
| TCGA-OV | Primary Tumor | TCGA-61-1995-01A-01R-1568-13 | TCGA-61-1995 |
| TCGA-OV | Primary Tumor | TCGA-61-1998-01A-01R-1568-13 | TCGA-61-1998 |
| TCGA-OV | Primary Tumor | TCGA-61-2000-01A-01R-1568-13 | TCGA-61-2000 |
| TCGA-OV | Primary Tumor | TCGA-61-2002-01A-01R-1568-13 | TCGA-61-2002 |
| TCGA-OV | Primary Tumor | TCGA-61-2003-01A-01R-1568-13 | TCGA-61-2003 |
| TCGA-OV | Primary Tumor | TCGA-61-2008-01A-02R-1568-13 | TCGA-61-2008 |
| TCGA-OV | Primary Tumor | TCGA-61-2009-01A-01R-1568-13 | TCGA-61-2009 |
| TCGA-OV | Primary Tumor | TCGA-61-2012-01A-01R-1568-13 | TCGA-61-2012 |
| TCGA-OV | Primary Tumor | TCGA-61-2016-01A-01R-1568-13 | TCGA-61-2016 |

| | | | |
|---|---|---|---|
| TCGA-OV | Primary Tumor | TCGA-61-2088-01A-01R-1568-13 | TCGA-61-2088 |
| TCGA-OV | Primary Tumor | TCGA-61-2092-01A-01R-1568-13 | TCGA-61-2092 |
| TCGA-OV | Primary Tumor | TCGA-61-2094-01A-01R-1568-13 | TCGA-61-2094 |
| TCGA-OV | Primary Tumor | TCGA-61-2095-01A-01R-1568-13 | TCGA-61-2095 |
| TCGA-OV | Primary Tumor | TCGA-61-2097-01A-02R-1568-13 | TCGA-61-2097 |
| TCGA-OV | Primary Tumor | TCGA-61-2098-01A-01R-1568-13 | TCGA-61-2098 |
| TCGA-OV | Primary Tumor | TCGA-61-2101-01A-01R-1568-13 | TCGA-61-2101 |
| TCGA-OV | Primary Tumor | TCGA-61-2102-01A-01R-1568-13 | TCGA-61-2102 |
| TCGA-OV | Primary Tumor | TCGA-61-2104-01A-01R-1568-13 | TCGA-61-2104 |
| TCGA-OV | Primary Tumor | TCGA-61-2109-01A-01R-1568-13 | TCGA-61-2109 |
| TCGA-OV | Primary Tumor | TCGA-61-2110-01A-01R-1568-13 | TCGA-61-2110 |
| TCGA-OV | Primary Tumor | TCGA-61-2111-01A-01R-1568-13 | TCGA-61-2111 |
| TCGA-OV | Primary Tumor | TCGA-61-2113-01A-01R-1568-13 | TCGA-61-2113 |
| TCGA-OV | Primary Tumor | TCGA-72-4232-01A-01R-1013-13 | TCGA-72-4232 |
| TCGA-OV | Primary Tumor | TCGA-72-4236-01A-01R-1013-13 | TCGA-72-4236 |
| TCGA-OV | Primary Tumor | TCGA-72-4237-01A-01R-1013-13 | TCGA-72-4237 |
| TCGA-OV | Primary Tumor | TCGA-OY-A56P-01A-12R-A407-13 | TCGA-OY-A56P |
| TCGA-OV | Primary Tumor | TCGA-OY-A56Q-01A-11R-A407-13 | TCGA-OY-A56Q |
| TCGA-OV | Primary Tumor | TCGA-VG-A8LO-01A-11R-A407-13 | TCGA-VG-A8LO |
| TCGA-OV | Primary Tumor | TCGA-WR-A838-01A-12R-A407-13 | TCGA-WR-A838 |
| TCGA-BRCA | Primary Tumor | TCGA-3C-AAAU-01A-11R-A41G-13 | TCGA-3C-AAAU |
| TCGA-BRCA | Primary Tumor | TCGA-3C-AALI-01A-11R-A41G-13 | TCGA-3C-AALI |
| TCGA-BRCA | Primary Tumor | TCGA-3C-AALJ-01A-31R-A41G-13 | TCGA-3C-AALJ |
| TCGA-BRCA | Primary Tumor | TCGA-3C-AALK-01A-11R-A41G-13 | TCGA-3C-AALK |
| TCGA-BRCA | Primary Tumor | TCGA-4H-AAAK-01A-12R-A41G-13 | TCGA-4H-AAAK |
| TCGA-BRCA | Primary Tumor | TCGA-5L-AAT0-01A-12R-A41G-13 | TCGA-5L-AAT0 |
| TCGA-BRCA | Primary Tumor | TCGA-5L-AAT1-01A-12R-A41G-13 | TCGA-5L-AAT1 |
| TCGA-BRCA | Primary Tumor | TCGA-5T-A9QA-01A-11R-A41G-13 | TCGA-5T-A9QA |
| TCGA-BRCA | Primary Tumor | TCGA-A1-A0SB-01A-11R-A143-13 | TCGA-A1-A0SB |
| TCGA-BRCA | Primary Tumor | TCGA-A1-A0SD-01A-11R-A114-13 | TCGA-A1-A0SD |
| TCGA-BRCA | Primary Tumor | TCGA-A1-A0SE-01A-11R-A085-13 | TCGA-A1-A0SE |
| TCGA-BRCA | Primary Tumor | TCGA-A1-A0SF-01A-11R-A143-13 | TCGA-A1-A0SF |
| TCGA-BRCA | Primary Tumor | TCGA-A1-A0SG-01A-11R-A143-13 | TCGA-A1-A0SG |
| TCGA-BRCA | Primary Tumor | TCGA-A1-A0SH-01A-11R-A085-13 | TCGA-A1-A0SH |
| TCGA-BRCA | Primary Tumor | TCGA-A1-A0SI-01A-11R-A143-13 | TCGA-A1-A0SI |
| TCGA-BRCA | Primary Tumor | TCGA-A1-A0SJ-01A-11R-A085-13 | TCGA-A1-A0SJ |
| TCGA-BRCA | Primary Tumor | TCGA-A1-A0SK-01A-12R-A085-13 | TCGA-A1-A0SK |
| TCGA-BRCA | Primary Tumor | TCGA-A1-A0SM-01A-11R-A085-13 | TCGA-A1-A0SM |
| TCGA-BRCA | Primary Tumor | TCGA-A1-A0SN-01A-11R-A143-13 | TCGA-A1-A0SN |
| TCGA-BRCA | Primary Tumor | TCGA-A1-A0SO-01A-22R-A085-13 | TCGA-A1-A0SO |
| TCGA-BRCA | Primary Tumor | TCGA-A1-A0SP-01A-11R-A085-13 | TCGA-A1-A0SP |
| TCGA-BRCA | Primary Tumor | TCGA-A1-A0SQ-01A-21R-A143-13 | TCGA-A1-A0SQ |
| TCGA-BRCA | Primary Tumor | TCGA-A2-A04N-01A-11R-A114-13 | TCGA-A2-A04N |
| TCGA-BRCA | Primary Tumor | TCGA-A2-A04P-01A-31R-A035-13 | TCGA-A2-A04P |
| TCGA-BRCA | Primary Tumor | TCGA-A2-A04Q-01A-21R-A035-13 | TCGA-A2-A04Q |
| TCGA-BRCA | Primary Tumor | TCGA-A2-A04R-01A-41R-A108-13 | TCGA-A2-A04R |
| TCGA-BRCA | Primary Tumor | TCGA-A2-A04T-01A-21R-A035-13 | TCGA-A2-A04T |
| TCGA-BRCA | Primary Tumor | TCGA-A2-A04U-01A-11R-A114-13 | TCGA-A2-A04U |
| TCGA-BRCA | Primary Tumor | TCGA-A2-A04V-01A-21R-A035-13 | TCGA-A2-A04V |
| TCGA-BRCA | Primary Tumor | TCGA-A2-A04W-01A-31R-A114-13 | TCGA-A2-A04W |
| TCGA-BRCA | Primary Tumor | TCGA-A2-A04X-01A-21R-A035-13 | TCGA-A2-A04X |
| TCGA-BRCA | Primary Tumor | TCGA-A2-A04Y-01A-21R-A035-13 | TCGA-A2-A04Y |
| TCGA-BRCA | Primary Tumor | TCGA-A2-A0CK-01A-11R-A22I-13 | TCGA-A2-A0CK |
| TCGA-BRCA | Primary Tumor | TCGA-A2-A0CL-01A-11R-A114-13 | TCGA-A2-A0CL |
| TCGA-BRCA | Primary Tumor | TCGA-A2-A0CM-01A-31R-A035-13 | TCGA-A2-A0CM |
| TCGA-BRCA | Primary Tumor | TCGA-A2-A0CO-01A-13R-A22I-13 | TCGA-A2-A0CO |
| TCGA-BRCA | Primary Tumor | TCGA-A2-A0CP-01A-11R-A035-13 | TCGA-A2-A0CP |
| TCGA-BRCA | Primary Tumor | TCGA-A2-A0CQ-01A-21R-A035-13 | TCGA-A2-A0CQ |
| TCGA-BRCA | Primary Tumor | TCGA-A2-A0CR-01A-11R-A22I-13 | TCGA-A2-A0CR |

| | | | |
|---|---|---|---|
| TCGA-BRCA | Primary Tumor | TCGA-A2-A0CS-01A-11R-A114-13 | TCGA-A2-A0CS |
| TCGA-BRCA | Primary Tumor | TCGA-A2-A0CT-01A-31R-A057-13 | TCGA-A2-A0CT |
| TCGA-BRCA | Primary Tumor | TCGA-A2-A0CU-01A-12R-A035-13 | TCGA-A2-A0CU |
| TCGA-BRCA | Primary Tumor | TCGA-A2-A0CV-01A-31R-A114-13 | TCGA-A2-A0CV |
| TCGA-BRCA | Primary Tumor | TCGA-A2-A0CW-01A-21R-A114-13 | TCGA-A2-A0CW |
| TCGA-BRCA | Primary Tumor | TCGA-A2-A0CY-01A-12R-A035-13 | TCGA-A2-A0CY |
| TCGA-BRCA | Primary Tumor | TCGA-A2-A0CZ-01A-11R-A035-13 | TCGA-A2-A0CZ |
| TCGA-BRCA | Primary Tumor | TCGA-A2-A0D0-01A-11R-A010-13 | TCGA-A2-A0D0 |
| TCGA-BRCA | Primary Tumor | TCGA-A2-A0D1-01A-11R-A035-13 | TCGA-A2-A0D1 |
| TCGA-BRCA | Primary Tumor | TCGA-A2-A0D2-01A-21R-A035-13 | TCGA-A2-A0D2 |
| TCGA-BRCA | Primary Tumor | TCGA-A2-A0D3-01A-11R-A114-13 | TCGA-A2-A0D3 |
| TCGA-BRCA | Primary Tumor | TCGA-A2-A0D4-01A-11R-A038-13 | TCGA-A2-A0D4 |
| TCGA-BRCA | Primary Tumor | TCGA-A2-A0EM-01A-11R-A035-13 | TCGA-A2-A0EM |
| TCGA-BRCA | Primary Tumor | TCGA-A2-A0EN-01A-13R-A085-13 | TCGA-A2-A0EN |
| TCGA-BRCA | Primary Tumor | TCGA-A2-A0EO-01A-11R-A035-13 | TCGA-A2-A0EO |
| TCGA-BRCA | Primary Tumor | TCGA-A2-A0EP-01A-52R-A22V-13 | TCGA-A2-A0EP |
| TCGA-BRCA | Primary Tumor | TCGA-A2-A0EQ-01A-11R-A035-13 | TCGA-A2-A0EQ |
| TCGA-BRCA | Primary Tumor | TCGA-A2-A0ER-01A-21R-A035-13 | TCGA-A2-A0ER |
| TCGA-BRCA | Primary Tumor | TCGA-A2-A0ES-01A-11R-A114-13 | TCGA-A2-A0ES |
| TCGA-BRCA | Primary Tumor | TCGA-A2-A0ET-01A-31R-A035-13 | TCGA-A2-A0ET |
| TCGA-BRCA | Primary Tumor | TCGA-A2-A0EU-01A-22R-A057-13 | TCGA-A2-A0EU |
| TCGA-BRCA | Primary Tumor | TCGA-A2-A0EV-01A-11R-A035-13 | TCGA-A2-A0EV |
| TCGA-BRCA | Primary Tumor | TCGA-A2-A0EW-01A-21R-A114-13 | TCGA-A2-A0EW |
| TCGA-BRCA | Primary Tumor | TCGA-A2-A0EX-01A-21R-A035-13 | TCGA-A2-A0EX |
| TCGA-BRCA | Primary Tumor | TCGA-A2-A0EY-01A-11R-A035-13 | TCGA-A2-A0EY |
| TCGA-BRCA | Primary Tumor | TCGA-A2-A0ST-01A-12R-A085-13 | TCGA-A2-A0ST |
| TCGA-BRCA | Primary Tumor | TCGA-A2-A0SU-01A-11R-A085-13 | TCGA-A2-A0SU |
| TCGA-BRCA | Primary Tumor | TCGA-A2-A0SV-01A-11R-A085-13 | TCGA-A2-A0SV |
| TCGA-BRCA | Primary Tumor | TCGA-A2-A0SW-01A-11R-A085-13 | TCGA-A2-A0SW |
| TCGA-BRCA | Primary Tumor | TCGA-A2-A0SX-01A-12R-A085-13 | TCGA-A2-A0SX |
| TCGA-BRCA | Primary Tumor | TCGA-A2-A0SY-01A-31R-A085-13 | TCGA-A2-A0SY |
| TCGA-BRCA | Primary Tumor | TCGA-A2-A0T0-01A-22R-A085-13 | TCGA-A2-A0T0 |
| TCGA-BRCA | Primary Tumor | TCGA-A2-A0T1-01A-21R-A085-13 | TCGA-A2-A0T1 |
| TCGA-BRCA | Primary Tumor | TCGA-A2-A0T2-01A-11R-A085-13 | TCGA-A2-A0T2 |
| TCGA-BRCA | Primary Tumor | TCGA-A2-A0T3-01A-21R-A114-13 | TCGA-A2-A0T3 |
| TCGA-BRCA | Primary Tumor | TCGA-A2-A0T4-01A-31R-A085-13 | TCGA-A2-A0T4 |
| TCGA-BRCA | Primary Tumor | TCGA-A2-A0T5-01A-21R-A085-13 | TCGA-A2-A0T5 |
| TCGA-BRCA | Primary Tumor | TCGA-A2-A0T6-01A-11R-A085-13 | TCGA-A2-A0T6 |
| TCGA-BRCA | Primary Tumor | TCGA-A2-A0T7-01A-21R-A085-13 | TCGA-A2-A0T7 |
| TCGA-BRCA | Primary Tumor | TCGA-A2-A0YC-01A-11R-A108-13 | TCGA-A2-A0YC |
| TCGA-BRCA | Primary Tumor | TCGA-A2-A0YD-01A-11R-A108-13 | TCGA-A2-A0YD |
| TCGA-BRCA | Primary Tumor | TCGA-A2-A0YF-01A-21R-A108-13 | TCGA-A2-A0YF |
| TCGA-BRCA | Primary Tumor | TCGA-A2-A0YG-01A-21R-A108-13 | TCGA-A2-A0YG |
| TCGA-BRCA | Primary Tumor | TCGA-A2-A0YH-01A-11R-A108-13 | TCGA-A2-A0YH |
| TCGA-BRCA | Primary Tumor | TCGA-A2-A0YI-01A-31R-A10I-13 | TCGA-A2-A0YI |
| TCGA-BRCA | Primary Tumor | TCGA-A2-A0YK-01A-22R-A108-13 | TCGA-A2-A0YK |
| TCGA-BRCA | Primary Tumor | TCGA-A2-A0YL-01A-21R-A108-13 | TCGA-A2-A0YL |
| TCGA-BRCA | Primary Tumor | TCGA-A2-A0YM-01A-11R-A108-13 | TCGA-A2-A0YM |
| TCGA-BRCA | Primary Tumor | TCGA-A2-A0YT-01A-11R-A108-13 | TCGA-A2-A0YT |
| TCGA-BRCA | Primary Tumor | TCGA-A2-A1FV-01A-11R-A13P-13 | TCGA-A2-A1FV |
| TCGA-BRCA | Primary Tumor | TCGA-A2-A1FW-01A-11R-A13P-13 | TCGA-A2-A1FW |
| TCGA-BRCA | Primary Tumor | TCGA-A2-A1FX-01A-11R-A13P-13 | TCGA-A2-A1FX |
| TCGA-BRCA | Primary Tumor | TCGA-A2-A1FZ-01A-51R-A14C-13 | TCGA-A2-A1FZ |
| TCGA-BRCA | Primary Tumor | TCGA-A2-A1G0-01A-11R-A13P-13 | TCGA-A2-A1G0 |
| TCGA-BRCA | Primary Tumor | TCGA-A2-A1G1-01A-21R-A13P-13 | TCGA-A2-A1G1 |
| TCGA-BRCA | Primary Tumor | TCGA-A2-A1G4-01A-11R-A13P-13 | TCGA-A2-A1G4 |
| TCGA-BRCA | Primary Tumor | TCGA-A2-A1G6-01A-11R-A13P-13 | TCGA-A2-A1G6 |
| TCGA-BRCA | Primary Tumor | TCGA-A2-A259-01A-11R-A16E-13 | TCGA-A2-A259 |
| TCGA-BRCA | Primary Tumor | TCGA-A2-A25A-01A-12R-A16E-13 | TCGA-A2-A25A |

| | | | |
|---|---|---|---|
| TCGA-BRCA | Primary Tumor | TCGA-A2-A25B-01A-11R-A168-13 | TCGA-A2-A25B |
| TCGA-BRCA | Primary Tumor | TCGA-A2-A25C-01A-11R-A168-13 | TCGA-A2-A25C |
| TCGA-BRCA | Primary Tumor | TCGA-A2-A25D-01A-12R-A16E-13 | TCGA-A2-A25D |
| TCGA-BRCA | Primary Tumor | TCGA-A2-A25E-01A-11R-A168-13 | TCGA-A2-A25E |
| TCGA-BRCA | Primary Tumor | TCGA-A2-A25F-01A-11R-A168-13 | TCGA-A2-A25F |
| TCGA-BRCA | Primary Tumor | TCGA-A2-A3KC-01A-11R-A214-13 | TCGA-A2-A3KC |
| TCGA-BRCA | Primary Tumor | TCGA-A2-A3KD-01A-12R-A214-13 | TCGA-A2-A3KD |
| TCGA-BRCA | Primary Tumor | TCGA-A2-A3XS-01A-11R-A22V-13 | TCGA-A2-A3XS |
| TCGA-BRCA | Primary Tumor | TCGA-A2-A3XT-01A-11R-A22V-13 | TCGA-A2-A3XT |
| TCGA-BRCA | Primary Tumor | TCGA-A2-A3XU-01A-12R-A22V-13 | TCGA-A2-A3XU |
| TCGA-BRCA | Primary Tumor | TCGA-A2-A3XV-01A-21R-A23A-13 | TCGA-A2-A3XV |
| TCGA-BRCA | Primary Tumor | TCGA-A2-A3XW-01A-11R-A23A-13 | TCGA-A2-A3XW |
| TCGA-BRCA | Primary Tumor | TCGA-A2-A3XX-01A-21R-A23A-13 | TCGA-A2-A3XX |
| TCGA-BRCA | Primary Tumor | TCGA-A2-A3XY-01A-11R-A23A-13 | TCGA-A2-A3XY |
| TCGA-BRCA | Primary Tumor | TCGA-A2-A3XZ-01A-42R-A23A-13 | TCGA-A2-A3XZ |
| TCGA-BRCA | Primary Tumor | TCGA-A2-A3Y0-01A-11R-A23A-13 | TCGA-A2-A3Y0 |
| TCGA-BRCA | Primary Tumor | TCGA-A2-A4RW-01A-21R-A25Z-13 | TCGA-A2-A4RW |
| TCGA-BRCA | Primary Tumor | TCGA-A2-A4RX-01A-11R-A25Z-13 | TCGA-A2-A4RX |
| TCGA-BRCA | Primary Tumor | TCGA-A2-A4RY-01A-31R-A25Z-13 | TCGA-A2-A4RY |
| TCGA-BRCA | Primary Tumor | TCGA-A2-A4S0-01A-21R-A25Z-13 | TCGA-A2-A4S0 |
| TCGA-BRCA | Primary Tumor | TCGA-A2-A4S1-01A-21R-A25Z-13 | TCGA-A2-A4S1 |
| TCGA-BRCA | Primary Tumor | TCGA-A2-A4S2-01A-12R-A25Z-13 | TCGA-A2-A4S2 |
| TCGA-BRCA | Primary Tumor | TCGA-A2-A4S3-01A-21R-A25Z-13 | TCGA-A2-A4S3 |
| TCGA-BRCA | Primary Tumor | TCGA-A7-A0CD-01A-11R-A010-13 | TCGA-A7-A0CD |
| TCGA-BRCA | Primary Tumor | TCGA-A7-A0CE-01A-11R-A010-13 | TCGA-A7-A0CE |
| TCGA-BRCA | Primary Tumor | TCGA-A7-A0CG-01A-11R-A038-13 | TCGA-A7-A0CG |
| TCGA-BRCA | Primary Tumor | TCGA-A7-A0CH-01A-21R-A038-13 | TCGA-A7-A0CH |
| TCGA-BRCA | Primary Tumor | TCGA-A7-A0CJ-01A-21R-A010-13 | TCGA-A7-A0CJ |
| TCGA-BRCA | Primary Tumor | TCGA-A7-A0D9-01A-31R-A057-13 | TCGA-A7-A0D9 |
| TCGA-BRCA | Primary Tumor | TCGA-A7-A0DA-01A-31R-A114-13 | TCGA-A7-A0DA |
| TCGA-BRCA | Primary Tumor | TCGA-A7-A0DB-01A-11R-A010-13 | TCGA-A7-A0DB |
| TCGA-BRCA | Primary Tumor | TCGA-A7-A0DB-01A-11R-A27D-13 | TCGA-A7-A0DB |
| TCGA-BRCA | Primary Tumor | TCGA-A7-A0DB-01C-02R-A27D-13 | TCGA-A7-A0DB |
| TCGA-BRCA | Primary Tumor | TCGA-A7-A0DC-01A-11R-A010-13 | TCGA-A7-A0DC |
| TCGA-BRCA | Primary Tumor | TCGA-A7-A0DC-01B-04R-A22P-13 | TCGA-A7-A0DC |
| TCGA-BRCA | Primary Tumor | TCGA-A7-A13D-01A-13R-A12O-13 | TCGA-A7-A13D |
| TCGA-BRCA | Primary Tumor | TCGA-A7-A13D-01A-13R-A27D-13 | TCGA-A7-A13D |
| TCGA-BRCA | Primary Tumor | TCGA-A7-A13D-01B-04R-A27D-13 | TCGA-A7-A13D |
| TCGA-BRCA | Primary Tumor | TCGA-A7-A13E-01A-11R-A12O-13 | TCGA-A7-A13E |
| TCGA-BRCA | Primary Tumor | TCGA-A7-A13E-01A-11R-A27D-13 | TCGA-A7-A13E |
| TCGA-BRCA | Primary Tumor | TCGA-A7-A13E-01B-06R-A27D-13 | TCGA-A7-A13E |
| TCGA-BRCA | Primary Tumor | TCGA-A7-A13F-01A-11R-A12O-13 | TCGA-A7-A13F |
| TCGA-BRCA | Primary Tumor | TCGA-A7-A13G-01A-11R-A13P-13 | TCGA-A7-A13G |
| TCGA-BRCA | Primary Tumor | TCGA-A7-A13G-01B-04R-A22P-13 | TCGA-A7-A13G |
| TCGA-BRCA | Primary Tumor | TCGA-A7-A13H-01A-11R-A22I-13 | TCGA-A7-A13H |
| TCGA-BRCA | Primary Tumor | TCGA-A7-A26E-01A-11R-A168-13 | TCGA-A7-A26E |
| TCGA-BRCA | Primary Tumor | TCGA-A7-A26E-01A-11R-A27D-13 | TCGA-A7-A26E |
| TCGA-BRCA | Primary Tumor | TCGA-A7-A26E-01B-06R-A27D-13 | TCGA-A7-A26E |
| TCGA-BRCA | Primary Tumor | TCGA-A7-A26F-01A-21R-A168-13 | TCGA-A7-A26F |
| TCGA-BRCA | Primary Tumor | TCGA-A7-A26F-01B-04R-A22P-13 | TCGA-A7-A26F |
| TCGA-BRCA | Primary Tumor | TCGA-A7-A26G-01A-21R-A168-13 | TCGA-A7-A26G |
| TCGA-BRCA | Primary Tumor | TCGA-A7-A26H-01A-11R-A168-13 | TCGA-A7-A26H |
| TCGA-BRCA | Primary Tumor | TCGA-A7-A26I-01A-11R-A168-13 | TCGA-A7-A26I |
| TCGA-BRCA | Primary Tumor | TCGA-A7-A26I-01B-06R-A22P-13 | TCGA-A7-A26I |
| TCGA-BRCA | Primary Tumor | TCGA-A7-A26J-01A-11R-A168-13 | TCGA-A7-A26J |
| TCGA-BRCA | Primary Tumor | TCGA-A7-A26J-01A-11R-A27D-13 | TCGA-A7-A26J |
| TCGA-BRCA | Primary Tumor | TCGA-A7-A26J-01B-02R-A27D-13 | TCGA-A7-A26J |
| TCGA-BRCA | Primary Tumor | TCGA-A7-A2KD-01A-31R-A21U-13 | TCGA-A7-A2KD |
| TCGA-BRCA | Primary Tumor | TCGA-A7-A3IY-01A-21R-A21U-13 | TCGA-A7-A3IY |

| | | | |
|---|---|---|---|
| TCGA-BRCA | Primary Tumor | TCGA-A7-A3IZ-01A-11R-A214-13 | TCGA-A7-A3IZ |
| TCGA-BRCA | Primary Tumor | TCGA-A7-A3J0-01A-11R-A214-13 | TCGA-A7-A3J0 |
| TCGA-BRCA | Primary Tumor | TCGA-A7-A3J1-01A-11R-A214-13 | TCGA-A7-A3J1 |
| TCGA-BRCA | Primary Tumor | TCGA-A7-A3RF-01A-11R-A22I-13 | TCGA-A7-A3RF |
| TCGA-BRCA | Primary Tumor | TCGA-A7-A425-01A-11R-A24J-13 | TCGA-A7-A425 |
| TCGA-BRCA | Primary Tumor | TCGA-A7-A426-01A-22R-A24J-13 | TCGA-A7-A426 |
| TCGA-BRCA | Primary Tumor | TCGA-A7-A4SA-01A-11R-A25Z-13 | TCGA-A7-A4SA |
| TCGA-BRCA | Primary Tumor | TCGA-A7-A4SB-01A-21R-A25Z-13 | TCGA-A7-A4SB |
| TCGA-BRCA | Primary Tumor | TCGA-A7-A4SC-01A-12R-A25Z-13 | TCGA-A7-A4SC |
| TCGA-BRCA | Primary Tumor | TCGA-A7-A4SD-01A-11R-A25Z-13 | TCGA-A7-A4SD |
| TCGA-BRCA | Primary Tumor | TCGA-A7-A4SE-01A-11R-A25Z-13 | TCGA-A7-A4SE |
| TCGA-BRCA | Primary Tumor | TCGA-A7-A4SF-01A-11R-A25Z-13 | TCGA-A7-A4SF |
| TCGA-BRCA | Primary Tumor | TCGA-A7-A56D-01A-11R-A27U-13 | TCGA-A7-A56D |
| TCGA-BRCA | Primary Tumor | TCGA-A7-A5ZV-01A-11R-A28I-13 | TCGA-A7-A5ZV |
| TCGA-BRCA | Primary Tumor | TCGA-A7-A5ZW-01A-12R-A29V-13 | TCGA-A7-A5ZW |
| TCGA-BRCA | Primary Tumor | TCGA-A7-A5ZX-01A-12R-A29V-13 | TCGA-A7-A5ZX |
| TCGA-BRCA | Primary Tumor | TCGA-A7-A6VV-01A-22R-A33A-13 | TCGA-A7-A6VV |
| TCGA-BRCA | Primary Tumor | TCGA-A7-A6VW-01A-21R-A33A-13 | TCGA-A7-A6VW |
| TCGA-BRCA | Primary Tumor | TCGA-A7-A6VX-01A-12R-A33A-13 | TCGA-A7-A6VX |
| TCGA-BRCA | Primary Tumor | TCGA-A7-A6VY-01A-12R-A33A-13 | TCGA-A7-A6VY |
| TCGA-BRCA | Primary Tumor | TCGA-A8-A06N-01A-11R-A010-13 | TCGA-A8-A06N |
| TCGA-BRCA | Primary Tumor | TCGA-A8-A06O-01A-11R-A010-13 | TCGA-A8-A06O |
| TCGA-BRCA | Primary Tumor | TCGA-A8-A06P-01A-11R-A010-13 | TCGA-A8-A06P |
| TCGA-BRCA | Primary Tumor | TCGA-A8-A06Q-01A-11R-A035-13 | TCGA-A8-A06Q |
| TCGA-BRCA | Primary Tumor | TCGA-A8-A06R-01A-11R-A010-13 | TCGA-A8-A06R |
| TCGA-BRCA | Primary Tumor | TCGA-A8-A06T-01A-11R-A010-13 | TCGA-A8-A06T |
| TCGA-BRCA | Primary Tumor | TCGA-A8-A06U-01A-11R-A010-13 | TCGA-A8-A06U |
| TCGA-BRCA | Primary Tumor | TCGA-A8-A06Y-01A-21R-A010-13 | TCGA-A8-A06Y |
| TCGA-BRCA | Primary Tumor | TCGA-A8-A06Z-01A-11R-A010-13 | TCGA-A8-A06Z |
| TCGA-BRCA | Primary Tumor | TCGA-A8-A075-01A-11R-A085-13 | TCGA-A8-A075 |
| TCGA-BRCA | Primary Tumor | TCGA-A8-A076-01A-21R-A010-13 | TCGA-A8-A076 |
| TCGA-BRCA | Primary Tumor | TCGA-A8-A079-01A-21R-A010-13 | TCGA-A8-A079 |
| TCGA-BRCA | Primary Tumor | TCGA-A8-A07B-01A-11R-A010-13 | TCGA-A8-A07B |
| TCGA-BRCA | Primary Tumor | TCGA-A8-A07C-01A-11R-A035-13 | TCGA-A8-A07C |
| TCGA-BRCA | Primary Tumor | TCGA-A8-A07E-01A-11R-A035-13 | TCGA-A8-A07E |
| TCGA-BRCA | Primary Tumor | TCGA-A8-A07F-01A-11R-A038-13 | TCGA-A8-A07F |
| TCGA-BRCA | Primary Tumor | TCGA-A8-A07G-01A-11R-A035-13 | TCGA-A8-A07G |
| TCGA-BRCA | Primary Tumor | TCGA-A8-A07I-01A-11R-A010-13 | TCGA-A8-A07I |
| TCGA-BRCA | Primary Tumor | TCGA-A8-A07J-01A-21R-A057-13 | TCGA-A8-A07J |
| TCGA-BRCA | Primary Tumor | TCGA-A8-A07L-01A-11R-A010-13 | TCGA-A8-A07L |
| TCGA-BRCA | Primary Tumor | TCGA-A8-A07O-01A-11R-A038-13 | TCGA-A8-A07O |
| TCGA-BRCA | Primary Tumor | TCGA-A8-A07P-01A-11R-A010-13 | TCGA-A8-A07P |
| TCGA-BRCA | Primary Tumor | TCGA-A8-A07R-01A-21R-A035-13 | TCGA-A8-A07R |
| TCGA-BRCA | Primary Tumor | TCGA-A8-A07S-01A-11R-A035-13 | TCGA-A8-A07S |
| TCGA-BRCA | Primary Tumor | TCGA-A8-A07U-01A-11R-A035-13 | TCGA-A8-A07U |
| TCGA-BRCA | Primary Tumor | TCGA-A8-A07W-01A-11R-A010-13 | TCGA-A8-A07W |
| TCGA-BRCA | Primary Tumor | TCGA-A8-A07Z-01A-11R-A010-13 | TCGA-A8-A07Z |
| TCGA-BRCA | Primary Tumor | TCGA-A8-A081-01A-11R-A010-13 | TCGA-A8-A081 |
| TCGA-BRCA | Primary Tumor | TCGA-A8-A082-01A-11R-A010-13 | TCGA-A8-A082 |
| TCGA-BRCA | Primary Tumor | TCGA-A8-A083-01A-21R-A010-13 | TCGA-A8-A083 |
| TCGA-BRCA | Primary Tumor | TCGA-A8-A084-01A-21R-A010-13 | TCGA-A8-A084 |
| TCGA-BRCA | Primary Tumor | TCGA-A8-A085-01A-11R-A010-13 | TCGA-A8-A085 |
| TCGA-BRCA | Primary Tumor | TCGA-A8-A086-01A-11R-A010-13 | TCGA-A8-A086 |
| TCGA-BRCA | Primary Tumor | TCGA-A8-A08A-01A-11R-A010-13 | TCGA-A8-A08A |
| TCGA-BRCA | Primary Tumor | TCGA-A8-A08B-01A-11R-A010-13 | TCGA-A8-A08B |
| TCGA-BRCA | Primary Tumor | TCGA-A8-A08G-01A-11R-A010-13 | TCGA-A8-A08G |
| TCGA-BRCA | Primary Tumor | TCGA-A8-A08H-01A-21R-A010-13 | TCGA-A8-A08H |
| TCGA-BRCA | Primary Tumor | TCGA-A8-A08I-01A-11R-A010-13 | TCGA-A8-A08I |
| TCGA-BRCA | Primary Tumor | TCGA-A8-A08J-01A-11R-A010-13 | TCGA-A8-A08J |

| | | | |
|---|---|---|---|
| TCGA-BRCA | Primary Tumor | TCGA-A8-A08L-01A-11R-A010-13 | TCGA-A8-A08L |
| TCGA-BRCA | Primary Tumor | TCGA-A8-A08O-01A-21R-A057-13 | TCGA-A8-A08O |
| TCGA-BRCA | Primary Tumor | TCGA-A8-A08R-01A-11R-A035-13 | TCGA-A8-A08R |
| TCGA-BRCA | Primary Tumor | TCGA-A8-A08S-01A-11R-A035-13 | TCGA-A8-A08S |
| TCGA-BRCA | Primary Tumor | TCGA-A8-A08T-01A-21R-A010-13 | TCGA-A8-A08T |
| TCGA-BRCA | Primary Tumor | TCGA-A8-A08X-01A-21R-A010-13 | TCGA-A8-A08X |
| TCGA-BRCA | Primary Tumor | TCGA-A8-A08Z-01A-21R-A010-13 | TCGA-A8-A08Z |
| TCGA-BRCA | Primary Tumor | TCGA-A8-A090-01A-11R-A010-13 | TCGA-A8-A090 |
| TCGA-BRCA | Primary Tumor | TCGA-A8-A091-01A-11R-A010-13 | TCGA-A8-A091 |
| TCGA-BRCA | Primary Tumor | TCGA-A8-A092-01A-11R-A010-13 | TCGA-A8-A092 |
| TCGA-BRCA | Primary Tumor | TCGA-A8-A093-01A-11R-A010-13 | TCGA-A8-A093 |
| TCGA-BRCA | Primary Tumor | TCGA-A8-A094-01A-11R-A038-13 | TCGA-A8-A094 |
| TCGA-BRCA | Primary Tumor | TCGA-A8-A095-01A-11R-A010-13 | TCGA-A8-A095 |
| TCGA-BRCA | Primary Tumor | TCGA-A8-A096-01A-11R-A038-13 | TCGA-A8-A096 |
| TCGA-BRCA | Primary Tumor | TCGA-A8-A097-01A-11R-A035-13 | TCGA-A8-A097 |
| TCGA-BRCA | Primary Tumor | TCGA-A8-A099-01A-11R-A010-13 | TCGA-A8-A099 |
| TCGA-BRCA | Primary Tumor | TCGA-A8-A09A-01A-11R-A038-13 | TCGA-A8-A09A |
| TCGA-BRCA | Primary Tumor | TCGA-A8-A09B-01A-11R-A010-13 | TCGA-A8-A09B |
| TCGA-BRCA | Primary Tumor | TCGA-A8-A09C-01A-11R-A010-13 | TCGA-A8-A09C |
| TCGA-BRCA | Primary Tumor | TCGA-A8-A09D-01A-11R-A010-13 | TCGA-A8-A09D |
| TCGA-BRCA | Primary Tumor | TCGA-A8-A09E-01A-11R-A010-13 | TCGA-A8-A09E |
| TCGA-BRCA | Primary Tumor | TCGA-A8-A09G-01A-21R-A010-13 | TCGA-A8-A09G |
| TCGA-BRCA | Primary Tumor | TCGA-A8-A09I-01A-22R-A035-13 | TCGA-A8-A09I |
| TCGA-BRCA | Primary Tumor | TCGA-A8-A09K-01A-11R-A010-13 | TCGA-A8-A09K |
| TCGA-BRCA | Primary Tumor | TCGA-A8-A09M-01A-11R-A010-13 | TCGA-A8-A09M |
| TCGA-BRCA | Primary Tumor | TCGA-A8-A09N-01A-11R-A010-13 | TCGA-A8-A09N |
| TCGA-BRCA | Primary Tumor | TCGA-A8-A09Q-01A-11R-A010-13 | TCGA-A8-A09Q |
| TCGA-BRCA | Primary Tumor | TCGA-A8-A09R-01A-11R-A010-13 | TCGA-A8-A09R |
| TCGA-BRCA | Primary Tumor | TCGA-A8-A09T-01A-11R-A010-13 | TCGA-A8-A09T |
| TCGA-BRCA | Primary Tumor | TCGA-A8-A09X-01A-11R-A010-13 | TCGA-A8-A09X |
| TCGA-BRCA | Primary Tumor | TCGA-A8-A09Z-01A-11R-A010-13 | TCGA-A8-A09Z |
| TCGA-BRCA | Primary Tumor | TCGA-A8-A0A1-01A-11R-A010-13 | TCGA-A8-A0A1 |
| TCGA-BRCA | Primary Tumor | TCGA-A8-A0A2-01A-11R-A035-13 | TCGA-A8-A0A2 |
| TCGA-BRCA | Primary Tumor | TCGA-A8-A0A4-01A-11R-A010-13 | TCGA-A8-A0A4 |
| TCGA-BRCA | Primary Tumor | TCGA-A8-A0A6-01A-12R-A057-13 | TCGA-A8-A0A6 |
| TCGA-BRCA | Primary Tumor | TCGA-A8-A0A7-01A-11R-A010-13 | TCGA-A8-A0A7 |
| TCGA-BRCA | Primary Tumor | TCGA-A8-A0A9-01A-11R-A010-13 | TCGA-A8-A0A9 |
| TCGA-BRCA | Primary Tumor | TCGA-A8-A0AB-01A-11R-A035-13 | TCGA-A8-A0AB |
| TCGA-BRCA | Primary Tumor | TCGA-A8-A0AD-01A-11R-A057-13 | TCGA-A8-A0AD |
| TCGA-BRCA | Primary Tumor | TCGA-AC-A23C-01A-12R-A168-13 | TCGA-AC-A23C |
| TCGA-BRCA | Primary Tumor | TCGA-AC-A23E-01A-11R-A156-13 | TCGA-AC-A23E |
| TCGA-BRCA | Primary Tumor | TCGA-AC-A23G-01A-11R-A214-13 | TCGA-AC-A23G |
| TCGA-BRCA | Primary Tumor | TCGA-AC-A23H-01A-11R-A156-13 | TCGA-AC-A23H |
| TCGA-BRCA | Primary Tumor | TCGA-AC-A2B8-01A-11R-A17A-13 | TCGA-AC-A2B8 |
| TCGA-BRCA | Primary Tumor | TCGA-AC-A2BK-01A-11R-A21U-13 | TCGA-AC-A2BK |
| TCGA-BRCA | Primary Tumor | TCGA-AC-A2BM-01A-11R-A21U-13 | TCGA-AC-A2BM |
| TCGA-BRCA | Primary Tumor | TCGA-AC-A2FB-01A-11R-A17A-13 | TCGA-AC-A2FB |
| TCGA-BRCA | Primary Tumor | TCGA-AC-A2FE-01A-11R-A19V-13 | TCGA-AC-A2FE |
| TCGA-BRCA | Primary Tumor | TCGA-AC-A2FF-01A-11R-A17A-13 | TCGA-AC-A2FF |
| TCGA-BRCA | Primary Tumor | TCGA-AC-A2FG-01A-11R-A17A-13 | TCGA-AC-A2FG |
| TCGA-BRCA | Primary Tumor | TCGA-AC-A2FK-01A-12R-A17X-13 | TCGA-AC-A2FK |
| TCGA-BRCA | Primary Tumor | TCGA-AC-A2FM-01A-11R-A19V-13 | TCGA-AC-A2FM |
| TCGA-BRCA | Primary Tumor | TCGA-AC-A2FO-01A-11R-A17X-13 | TCGA-AC-A2FO |
| TCGA-BRCA | Primary Tumor | TCGA-AC-A2QH-01A-11R-A18L-13 | TCGA-AC-A2QH |
| TCGA-BRCA | Primary Tumor | TCGA-AC-A2QH-01B-04R-A22P-13 | TCGA-AC-A2QH |
| TCGA-BRCA | Primary Tumor | TCGA-AC-A2QI-01A-12R-A19V-13 | TCGA-AC-A2QI |
| TCGA-BRCA | Primary Tumor | TCGA-AC-A2QJ-01A-12R-A19V-13 | TCGA-AC-A2QJ |
| TCGA-BRCA | Primary Tumor | TCGA-AC-A3BB-01A-21R-A19V-13 | TCGA-AC-A3BB |
| TCGA-BRCA | Primary Tumor | TCGA-AC-A3EH-01A-22R-A22I-13 | TCGA-AC-A3EH |

| | | | |
|---|---|---|---|
| TCGA-BRCA | Primary Tumor | TCGA-AC-A3HN-01A-11R-A214-13 | TCGA-AC-A3HN |
| TCGA-BRCA | Primary Tumor | TCGA-AC-A3OD-01A-11R-A21U-13 | TCGA-AC-A3OD |
| TCGA-BRCA | Primary Tumor | TCGA-AC-A3OD-01B-06R-A22P-13 | TCGA-AC-A3OD |
| TCGA-BRCA | Primary Tumor | TCGA-AC-A3QP-01A-11R-A22V-13 | TCGA-AC-A3QP |
| TCGA-BRCA | Primary Tumor | TCGA-AC-A3QQ-01A-11R-A22I-13 | TCGA-AC-A3QQ |
| TCGA-BRCA | Primary Tumor | TCGA-AC-A3QQ-01B-06R-A22P-13 | TCGA-AC-A3QQ |
| TCGA-BRCA | Primary Tumor | TCGA-AC-A3TM-01A-11R-A22I-13 | TCGA-AC-A3TM |
| TCGA-BRCA | Primary Tumor | TCGA-AC-A3TN-01A-11R-A22I-13 | TCGA-AC-A3TN |
| TCGA-BRCA | Primary Tumor | TCGA-AC-A3W5-01A-11R-A22I-13 | TCGA-AC-A3W5 |
| TCGA-BRCA | Primary Tumor | TCGA-AC-A3W6-01A-12R-A22I-13 | TCGA-AC-A3W6 |
| TCGA-BRCA | Primary Tumor | TCGA-AC-A3W7-01A-11R-A22I-13 | TCGA-AC-A3W7 |
| TCGA-BRCA | Primary Tumor | TCGA-AC-A3YI-01A-21R-A23A-13 | TCGA-AC-A3YI |
| TCGA-BRCA | Primary Tumor | TCGA-AC-A3YJ-01A-11R-A22V-13 | TCGA-AC-A3YJ |
| TCGA-BRCA | Primary Tumor | TCGA-AC-A4ZE-01A-11R-A41G-13 | TCGA-AC-A4ZE |
| TCGA-BRCA | Primary Tumor | TCGA-AC-A5EH-01A-11R-A28I-13 | TCGA-AC-A5EH |
| TCGA-BRCA | Primary Tumor | TCGA-AC-A5EI-01A-11R-A27U-13 | TCGA-AC-A5EI |
| TCGA-BRCA | Primary Tumor | TCGA-AC-A5XS-01A-11R-A29V-13 | TCGA-AC-A5XS |
| TCGA-BRCA | Primary Tumor | TCGA-AC-A5XU-01A-11R-A28I-13 | TCGA-AC-A5XU |
| TCGA-BRCA | Primary Tumor | TCGA-AC-A62V-01A-11R-A31S-13 | TCGA-AC-A62V |
| TCGA-BRCA | Primary Tumor | TCGA-AC-A62X-01A-11R-A29V-13 | TCGA-AC-A62X |
| TCGA-BRCA | Primary Tumor | TCGA-AC-A62Y-01A-11R-A29V-13 | TCGA-AC-A62Y |
| TCGA-BRCA | Primary Tumor | TCGA-AC-A6IV-01A-12R-A33A-13 | TCGA-AC-A6IV |
| TCGA-BRCA | Primary Tumor | TCGA-AC-A6IW-01A-12R-A33A-13 | TCGA-AC-A6IW |
| TCGA-BRCA | Primary Tumor | TCGA-AC-A6IX-01A-12R-A32K-13 | TCGA-AC-A6IX |
| TCGA-BRCA | Primary Tumor | TCGA-AC-A6NO-01A-12R-A33A-13 | TCGA-AC-A6NO |
| TCGA-BRCA | Primary Tumor | TCGA-AC-A7VB-01A-11R-A358-13 | TCGA-AC-A7VB |
| TCGA-BRCA | Primary Tumor | TCGA-AC-A7VC-01A-11R-A358-13 | TCGA-AC-A7VC |
| TCGA-BRCA | Primary Tumor | TCGA-AC-A8OP-01A-11R-A36A-13 | TCGA-AC-A8OP |
| TCGA-BRCA | Primary Tumor | TCGA-AC-A8OQ-01A-11R-A41G-13 | TCGA-AC-A8OQ |
| TCGA-BRCA | Primary Tumor | TCGA-AC-A8OR-01A-21R-A41G-13 | TCGA-AC-A8OR |
| TCGA-BRCA | Primary Tumor | TCGA-AC-A8OS-01A-12R-A41G-13 | TCGA-AC-A8OS |
| TCGA-BRCA | Primary Tumor | TCGA-AN-A03X-01A-21R-A010-13 | TCGA-AN-A03X |
| TCGA-BRCA | Primary Tumor | TCGA-AN-A03Y-01A-21R-A010-13 | TCGA-AN-A03Y |
| TCGA-BRCA | Primary Tumor | TCGA-AN-A041-01A-11R-A035-13 | TCGA-AN-A041 |
| TCGA-BRCA | Primary Tumor | TCGA-AN-A046-01A-21R-A035-13 | TCGA-AN-A046 |
| TCGA-BRCA | Primary Tumor | TCGA-AN-A049-01A-21R-A010-13 | TCGA-AN-A049 |
| TCGA-BRCA | Primary Tumor | TCGA-AN-A04A-01A-21R-A035-13 | TCGA-AN-A04A |
| TCGA-BRCA | Primary Tumor | TCGA-AN-A04C-01A-21R-A035-13 | TCGA-AN-A04C |
| TCGA-BRCA | Primary Tumor | TCGA-AN-A04D-01A-21R-A035-13 | TCGA-AN-A04D |
| TCGA-BRCA | Primary Tumor | TCGA-AN-A0AJ-01A-11R-A010-13 | TCGA-AN-A0AJ |
| TCGA-BRCA | Primary Tumor | TCGA-AN-A0AK-01A-21R-A010-13 | TCGA-AN-A0AK |
| TCGA-BRCA | Primary Tumor | TCGA-AN-A0AL-01A-11R-A010-13 | TCGA-AN-A0AL |
| TCGA-BRCA | Primary Tumor | TCGA-AN-A0AM-01A-11R-A035-13 | TCGA-AN-A0AM |
| TCGA-BRCA | Primary Tumor | TCGA-AN-A0AR-01A-11R-A010-13 | TCGA-AN-A0AR |
| TCGA-BRCA | Primary Tumor | TCGA-AN-A0AS-01A-11R-A010-13 | TCGA-AN-A0AS |
| TCGA-BRCA | Primary Tumor | TCGA-AN-A0AT-01A-11R-A035-13 | TCGA-AN-A0AT |
| TCGA-BRCA | Primary Tumor | TCGA-AN-A0FD-01A-11R-A035-13 | TCGA-AN-A0FD |
| TCGA-BRCA | Primary Tumor | TCGA-AN-A0FF-01A-11R-A035-13 | TCGA-AN-A0FF |
| TCGA-BRCA | Primary Tumor | TCGA-AN-A0FJ-01A-11R-A010-13 | TCGA-AN-A0FJ |
| TCGA-BRCA | Primary Tumor | TCGA-AN-A0FK-01A-11R-A035-13 | TCGA-AN-A0FK |
| TCGA-BRCA | Primary Tumor | TCGA-AN-A0FL-01A-11R-A035-13 | TCGA-AN-A0FL |
| TCGA-BRCA | Primary Tumor | TCGA-AN-A0FN-01A-11R-A035-13 | TCGA-AN-A0FN |
| TCGA-BRCA | Primary Tumor | TCGA-AN-A0FS-01A-11R-A035-13 | TCGA-AN-A0FS |
| TCGA-BRCA | Primary Tumor | TCGA-AN-A0FT-01A-11R-A035-13 | TCGA-AN-A0FT |
| TCGA-BRCA | Primary Tumor | TCGA-AN-A0FV-01A-11R-A010-13 | TCGA-AN-A0FV |
| TCGA-BRCA | Primary Tumor | TCGA-AN-A0FW-01A-11R-A035-13 | TCGA-AN-A0FW |
| TCGA-BRCA | Primary Tumor | TCGA-AN-A0FX-01A-11R-A035-13 | TCGA-AN-A0FX |
| TCGA-BRCA | Primary Tumor | TCGA-AN-A0FY-01A-11R-A035-13 | TCGA-AN-A0FY |
| TCGA-BRCA | Primary Tumor | TCGA-AN-A0FZ-01A-11R-A035-13 | TCGA-AN-A0FZ |

| | | | |
|---|---|---|---|
| TCGA-BRCA | Primary Tumor | TCGA-AN-A0G0-01A-11R-A035-13 | TCGA-AN-A0G0 |
| TCGA-BRCA | Primary Tumor | TCGA-AN-A0XL-01A-11R-A10I-13 | TCGA-AN-A0XL |
| TCGA-BRCA | Primary Tumor | TCGA-AN-A0XN-01A-21R-A108-13 | TCGA-AN-A0XN |
| TCGA-BRCA | Primary Tumor | TCGA-AN-A0XO-01A-11R-A108-13 | TCGA-AN-A0XO |
| TCGA-BRCA | Primary Tumor | TCGA-AN-A0XP-01A-11R-A108-13 | TCGA-AN-A0XP |
| TCGA-BRCA | Primary Tumor | TCGA-AN-A0XR-01A-11R-A108-13 | TCGA-AN-A0XR |
| TCGA-BRCA | Primary Tumor | TCGA-AN-A0XS-01A-22R-A108-13 | TCGA-AN-A0XS |
| TCGA-BRCA | Primary Tumor | TCGA-AN-A0XT-01A-11R-A108-13 | TCGA-AN-A0XT |
| TCGA-BRCA | Primary Tumor | TCGA-AN-A0XU-01A-11R-A108-13 | TCGA-AN-A0XU |
| TCGA-BRCA | Primary Tumor | TCGA-AN-A0XV-01A-11R-A108-13 | TCGA-AN-A0XV |
| TCGA-BRCA | Primary Tumor | TCGA-AN-A0XW-01A-11R-A108-13 | TCGA-AN-A0XW |
| TCGA-BRCA | Primary Tumor | TCGA-AO-A03L-01A-41R-A057-13 | TCGA-AO-A03L |
| TCGA-BRCA | Primary Tumor | TCGA-AO-A03M-01B-11R-A10I-13 | TCGA-AO-A03M |
| TCGA-BRCA | Primary Tumor | TCGA-AO-A03N-01B-11R-A10I-13 | TCGA-AO-A03N |
| TCGA-BRCA | Primary Tumor | TCGA-AO-A03O-01A-11R-A010-13 | TCGA-AO-A03O |
| TCGA-BRCA | Primary Tumor | TCGA-AO-A03R-01A-21R-A035-13 | TCGA-AO-A03R |
| TCGA-BRCA | Primary Tumor | TCGA-AO-A03T-01A-21R-A035-13 | TCGA-AO-A03T |
| TCGA-BRCA | Primary Tumor | TCGA-AO-A03U-01B-21R-A10I-13 | TCGA-AO-A03U |
| TCGA-BRCA | Primary Tumor | TCGA-AO-A03V-01A-11R-A114-13 | TCGA-AO-A03V |
| TCGA-BRCA | Primary Tumor | TCGA-AO-A0J2-01A-11R-A035-13 | TCGA-AO-A0J2 |
| TCGA-BRCA | Primary Tumor | TCGA-AO-A0J3-01A-11R-A035-13 | TCGA-AO-A0J3 |
| TCGA-BRCA | Primary Tumor | TCGA-AO-A0J4-01A-11R-A035-13 | TCGA-AO-A0J4 |
| TCGA-BRCA | Primary Tumor | TCGA-AO-A0J5-01A-11R-A035-13 | TCGA-AO-A0J5 |
| TCGA-BRCA | Primary Tumor | TCGA-AO-A0J6-01A-11R-A035-13 | TCGA-AO-A0J6 |
| TCGA-BRCA | Primary Tumor | TCGA-AO-A0J7-01A-11R-A035-13 | TCGA-AO-A0J7 |
| TCGA-BRCA | Primary Tumor | TCGA-AO-A0J8-01A-21R-A035-13 | TCGA-AO-A0J8 |
| TCGA-BRCA | Primary Tumor | TCGA-AO-A0J9-01A-11R-A035-13 | TCGA-AO-A0J9 |
| TCGA-BRCA | Primary Tumor | TCGA-AO-A0JA-01A-11R-A057-13 | TCGA-AO-A0JA |
| TCGA-BRCA | Primary Tumor | TCGA-AO-A0JB-01A-11R-A057-13 | TCGA-AO-A0JB |
| TCGA-BRCA | Primary Tumor | TCGA-AO-A0JC-01A-11R-A057-13 | TCGA-AO-A0JC |
| TCGA-BRCA | Primary Tumor | TCGA-AO-A0JD-01A-11R-A057-13 | TCGA-AO-A0JD |
| TCGA-BRCA | Primary Tumor | TCGA-AO-A0JE-01A-11R-A057-13 | TCGA-AO-A0JE |
| TCGA-BRCA | Primary Tumor | TCGA-AO-A0JF-01A-11R-A057-13 | TCGA-AO-A0JF |
| TCGA-BRCA | Primary Tumor | TCGA-AO-A0JG-01A-31R-A085-13 | TCGA-AO-A0JG |
| TCGA-BRCA | Primary Tumor | TCGA-AO-A0JI-01A-21R-A057-13 | TCGA-AO-A0JI |
| TCGA-BRCA | Primary Tumor | TCGA-AO-A0JJ-01A-11R-A057-13 | TCGA-AO-A0JJ |
| TCGA-BRCA | Primary Tumor | TCGA-AO-A0JL-01A-11R-A057-13 | TCGA-AO-A0JL |
| TCGA-BRCA | Primary Tumor | TCGA-AO-A0JM-01A-21R-A057-13 | TCGA-AO-A0JM |
| TCGA-BRCA | Primary Tumor | TCGA-AO-A124-01A-11R-A10I-13 | TCGA-AO-A124 |
| TCGA-BRCA | Primary Tumor | TCGA-AO-A125-01A-11R-A10I-13 | TCGA-AO-A125 |
| TCGA-BRCA | Primary Tumor | TCGA-AO-A126-01A-11R-A10I-13 | TCGA-AO-A126 |
| TCGA-BRCA | Primary Tumor | TCGA-AO-A128-01A-11R-A10I-13 | TCGA-AO-A128 |
| TCGA-BRCA | Primary Tumor | TCGA-AO-A129-01A-21R-A10I-13 | TCGA-AO-A129 |
| TCGA-BRCA | Primary Tumor | TCGA-AO-A12A-01A-21R-A114-13 | TCGA-AO-A12A |
| TCGA-BRCA | Primary Tumor | TCGA-AO-A12B-01A-11R-A10I-13 | TCGA-AO-A12B |
| TCGA-BRCA | Primary Tumor | TCGA-AO-A12C-01A-11R-A10I-13 | TCGA-AO-A12C |
| TCGA-BRCA | Primary Tumor | TCGA-AO-A12D-01A-11R-A114-13 | TCGA-AO-A12D |
| TCGA-BRCA | Primary Tumor | TCGA-AO-A12E-01A-11R-A10I-13 | TCGA-AO-A12E |
| TCGA-BRCA | Primary Tumor | TCGA-AO-A12F-01A-11R-A114-13 | TCGA-AO-A12F |
| TCGA-BRCA | Primary Tumor | TCGA-AO-A12G-01A-11R-A10I-13 | TCGA-AO-A12G |
| TCGA-BRCA | Primary Tumor | TCGA-AO-A12H-01A-11R-A114-13 | TCGA-AO-A12H |
| TCGA-BRCA | Primary Tumor | TCGA-AO-A1KO-01A-31R-A13P-13 | TCGA-AO-A1KO |
| TCGA-BRCA | Primary Tumor | TCGA-AO-A1KP-01A-11R-A13P-13 | TCGA-AO-A1KP |
| TCGA-BRCA | Primary Tumor | TCGA-AO-A1KQ-01A-11R-A13P-13 | TCGA-AO-A1KQ |
| TCGA-BRCA | Primary Tumor | TCGA-AO-A1KR-01A-12R-A143-13 | TCGA-AO-A1KR |
| TCGA-BRCA | Primary Tumor | TCGA-AO-A1KS-01A-11R-A13P-13 | TCGA-AO-A1KS |
| TCGA-BRCA | Primary Tumor | TCGA-AO-A1KT-01A-11R-A13P-13 | TCGA-AO-A1KT |
| TCGA-BRCA | Primary Tumor | TCGA-AQ-A04H-01B-11R-A10I-13 | TCGA-AQ-A04H |
| TCGA-BRCA | Primary Tumor | TCGA-AQ-A04J-01A-02R-A035-13 | TCGA-AQ-A04J |

| | | | |
|---|---|---|---|
| TCGA-BRCA | Primary Tumor | TCGA-AQ-A04L-01B-21R-A10I-13 | TCGA-AQ-A04L |
| TCGA-BRCA | Primary Tumor | TCGA-AQ-A0Y5-01A-11R-A14L-13 | TCGA-AQ-A0Y5 |
| TCGA-BRCA | Primary Tumor | TCGA-AQ-A1H2-01A-11R-A13P-13 | TCGA-AQ-A1H2 |
| TCGA-BRCA | Primary Tumor | TCGA-AQ-A1H3-01A-31R-A13P-13 | TCGA-AQ-A1H3 |
| TCGA-BRCA | Primary Tumor | TCGA-AQ-A54N-01A-11R-A25Z-13 | TCGA-AQ-A54N |
| TCGA-BRCA | Primary Tumor | TCGA-AQ-A54O-01A-11R-A25Z-13 | TCGA-AQ-A54O |
| TCGA-BRCA | Primary Tumor | TCGA-AQ-A7U7-01A-22R-A358-13 | TCGA-AQ-A7U7 |
| TCGA-BRCA | Primary Tumor | TCGA-AR-A0TP-01A-11R-A085-13 | TCGA-AR-A0TP |
| TCGA-BRCA | Primary Tumor | TCGA-AR-A0TQ-01A-11R-A085-13 | TCGA-AR-A0TQ |
| TCGA-BRCA | Primary Tumor | TCGA-AR-A0TR-01A-11R-A085-13 | TCGA-AR-A0TR |
| TCGA-BRCA | Primary Tumor | TCGA-AR-A0TS-01A-11R-A114-13 | TCGA-AR-A0TS |
| TCGA-BRCA | Primary Tumor | TCGA-AR-A0TT-01A-31R-A085-13 | TCGA-AR-A0TT |
| TCGA-BRCA | Primary Tumor | TCGA-AR-A0TV-01A-21R-A085-13 | TCGA-AR-A0TV |
| TCGA-BRCA | Primary Tumor | TCGA-AR-A0TX-01A-11R-A085-13 | TCGA-AR-A0TX |
| TCGA-BRCA | Primary Tumor | TCGA-AR-A0TY-01A-12R-A114-13 | TCGA-AR-A0TY |
| TCGA-BRCA | Primary Tumor | TCGA-AR-A0TZ-01A-12R-A085-13 | TCGA-AR-A0TZ |
| TCGA-BRCA | Primary Tumor | TCGA-AR-A0U2-01A-11R-A108-13 | TCGA-AR-A0U2 |
| TCGA-BRCA | Primary Tumor | TCGA-AR-A0U3-01A-11R-A108-13 | TCGA-AR-A0U3 |
| TCGA-BRCA | Primary Tumor | TCGA-AR-A0U4-01A-11R-A108-13 | TCGA-AR-A0U4 |
| TCGA-BRCA | Primary Tumor | TCGA-AR-A1AH-01A-11R-A12C-13 | TCGA-AR-A1AH |
| TCGA-BRCA | Primary Tumor | TCGA-AR-A1AI-01A-11R-A12O-13 | TCGA-AR-A1AI |
| TCGA-BRCA | Primary Tumor | TCGA-AR-A1AJ-01A-21R-A12O-13 | TCGA-AR-A1AJ |
| TCGA-BRCA | Primary Tumor | TCGA-AR-A1AK-01A-21R-A12O-13 | TCGA-AR-A1AK |
| TCGA-BRCA | Primary Tumor | TCGA-AR-A1AL-01A-21R-A12O-13 | TCGA-AR-A1AL |
| TCGA-BRCA | Primary Tumor | TCGA-AR-A1AM-01A-41R-A22I-13 | TCGA-AR-A1AM |
| TCGA-BRCA | Primary Tumor | TCGA-AR-A1AN-01A-11R-A12O-13 | TCGA-AR-A1AN |
| TCGA-BRCA | Primary Tumor | TCGA-AR-A1AO-01A-11R-A12O-13 | TCGA-AR-A1AO |
| TCGA-BRCA | Primary Tumor | TCGA-AR-A1AP-01A-11R-A12O-13 | TCGA-AR-A1AP |
| TCGA-BRCA | Primary Tumor | TCGA-AR-A1AQ-01A-11R-A12O-13 | TCGA-AR-A1AQ |
| TCGA-BRCA | Primary Tumor | TCGA-AR-A1AR-01A-31R-A136-13 | TCGA-AR-A1AR |
| TCGA-BRCA | Primary Tumor | TCGA-AR-A1AS-01A-11R-A12O-13 | TCGA-AR-A1AS |
| TCGA-BRCA | Primary Tumor | TCGA-AR-A1AT-01A-11R-A12O-13 | TCGA-AR-A1AT |
| TCGA-BRCA | Primary Tumor | TCGA-AR-A1AU-01A-11R-A12O-13 | TCGA-AR-A1AU |
| TCGA-BRCA | Primary Tumor | TCGA-AR-A1AV-01A-21R-A12O-13 | TCGA-AR-A1AV |
| TCGA-BRCA | Primary Tumor | TCGA-AR-A1AW-01A-21R-A12O-13 | TCGA-AR-A1AW |
| TCGA-BRCA | Primary Tumor | TCGA-AR-A1AX-01A-11R-A12O-13 | TCGA-AR-A1AX |
| TCGA-BRCA | Primary Tumor | TCGA-AR-A1AY-01A-21R-A12O-13 | TCGA-AR-A1AY |
| TCGA-BRCA | Primary Tumor | TCGA-AR-A24H-01A-11R-A168-13 | TCGA-AR-A24H |
| TCGA-BRCA | Primary Tumor | TCGA-AR-A24K-01A-11R-A168-13 | TCGA-AR-A24K |
| TCGA-BRCA | Primary Tumor | TCGA-AR-A24L-01A-11R-A168-13 | TCGA-AR-A24L |
| TCGA-BRCA | Primary Tumor | TCGA-AR-A24M-01A-11R-A168-13 | TCGA-AR-A24M |
| TCGA-BRCA | Primary Tumor | TCGA-AR-A24N-01A-11R-A168-13 | TCGA-AR-A24N |
| TCGA-BRCA | Primary Tumor | TCGA-AR-A24O-01A-11R-A168-13 | TCGA-AR-A24O |
| TCGA-BRCA | Primary Tumor | TCGA-AR-A24P-01A-11R-A168-13 | TCGA-AR-A24P |
| TCGA-BRCA | Primary Tumor | TCGA-AR-A24Q-01A-12R-A168-13 | TCGA-AR-A24Q |
| TCGA-BRCA | Primary Tumor | TCGA-AR-A24R-01A-11R-A168-13 | TCGA-AR-A24R |
| TCGA-BRCA | Primary Tumor | TCGA-AR-A24S-01A-11R-A168-13 | TCGA-AR-A24S |
| TCGA-BRCA | Primary Tumor | TCGA-AR-A24T-01A-11R-A168-13 | TCGA-AR-A24T |
| TCGA-BRCA | Primary Tumor | TCGA-AR-A24U-01A-11R-A168-13 | TCGA-AR-A24U |
| TCGA-BRCA | Primary Tumor | TCGA-AR-A24V-01A-21R-A168-13 | TCGA-AR-A24V |
| TCGA-BRCA | Primary Tumor | TCGA-AR-A24W-01A-11R-A168-13 | TCGA-AR-A24W |
| TCGA-BRCA | Primary Tumor | TCGA-AR-A24X-01A-11R-A168-13 | TCGA-AR-A24X |
| TCGA-BRCA | Primary Tumor | TCGA-AR-A24Z-01A-11R-A168-13 | TCGA-AR-A24Z |
| TCGA-BRCA | Primary Tumor | TCGA-AR-A250-01A-31R-A168-13 | TCGA-AR-A250 |
| TCGA-BRCA | Primary Tumor | TCGA-AR-A251-01A-12R-A168-13 | TCGA-AR-A251 |
| TCGA-BRCA | Primary Tumor | TCGA-AR-A252-01A-11R-A168-13 | TCGA-AR-A252 |
| TCGA-BRCA | Primary Tumor | TCGA-AR-A254-01A-21R-A168-13 | TCGA-AR-A254 |
| TCGA-BRCA | Primary Tumor | TCGA-AR-A255-01A-11R-A168-13 | TCGA-AR-A255 |
| TCGA-BRCA | Primary Tumor | TCGA-AR-A256-01A-11R-A168-13 | TCGA-AR-A256 |

| | | | |
|---|---|---|---|
| TCGA-BRCA | Primary Tumor | TCGA-AR-A2LE-01A-11R-A17X-13 | TCGA-AR-A2LE |
| TCGA-BRCA | Primary Tumor | TCGA-AR-A2LH-01A-31R-A18L-13 | TCGA-AR-A2LH |
| TCGA-BRCA | Primary Tumor | TCGA-AR-A2LJ-01A-12R-A19V-13 | TCGA-AR-A2LJ |
| TCGA-BRCA | Primary Tumor | TCGA-AR-A2LK-01A-11R-A17X-13 | TCGA-AR-A2LK |
| TCGA-BRCA | Primary Tumor | TCGA-AR-A2LL-01A-11R-A17X-13 | TCGA-AR-A2LL |
| TCGA-BRCA | Primary Tumor | TCGA-AR-A2LM-01A-11R-A17X-13 | TCGA-AR-A2LM |
| TCGA-BRCA | Primary Tumor | TCGA-AR-A2LN-01A-21R-A18L-13 | TCGA-AR-A2LN |
| TCGA-BRCA | Primary Tumor | TCGA-AR-A2LO-01A-31R-A18L-13 | TCGA-AR-A2LO |
| TCGA-BRCA | Primary Tumor | TCGA-AR-A2LQ-01A-22R-A18L-13 | TCGA-AR-A2LQ |
| TCGA-BRCA | Primary Tumor | TCGA-AR-A2LR-01A-12R-A18L-13 | TCGA-AR-A2LR |
| TCGA-BRCA | Primary Tumor | TCGA-AR-A5QM-01A-11R-A27U-13 | TCGA-AR-A5QM |
| TCGA-BRCA | Primary Tumor | TCGA-AR-A5QN-01A-12R-A28I-13 | TCGA-AR-A5QN |
| TCGA-BRCA | Primary Tumor | TCGA-AR-A5QP-01A-11R-A28I-13 | TCGA-AR-A5QP |
| TCGA-BRCA | Primary Tumor | TCGA-AR-A5QQ-01A-11R-A28I-13 | TCGA-AR-A5QQ |
| TCGA-BRCA | Primary Tumor | TCGA-B6-A0I1-01A-11R-A21U-13 | TCGA-B6-A0I1 |
| TCGA-BRCA | Primary Tumor | TCGA-B6-A0I2-01A-11R-A035-13 | TCGA-B6-A0I2 |
| TCGA-BRCA | Primary Tumor | TCGA-B6-A0I5-01A-11R-A035-13 | TCGA-B6-A0I5 |
| TCGA-BRCA | Primary Tumor | TCGA-B6-A0I6-01A-11R-A035-13 | TCGA-B6-A0I6 |
| TCGA-BRCA | Primary Tumor | TCGA-B6-A0I8-01A-11R-A035-13 | TCGA-B6-A0I8 |
| TCGA-BRCA | Primary Tumor | TCGA-B6-A0I9-01A-11R-A035-13 | TCGA-B6-A0I9 |
| TCGA-BRCA | Primary Tumor | TCGA-B6-A0IA-01A-11R-A035-13 | TCGA-B6-A0IA |
| TCGA-BRCA | Primary Tumor | TCGA-B6-A0IB-01A-11R-A035-13 | TCGA-B6-A0IB |
| TCGA-BRCA | Primary Tumor | TCGA-B6-A0IC-01A-11R-A035-13 | TCGA-B6-A0IC |
| TCGA-BRCA | Primary Tumor | TCGA-B6-A0IE-01A-11R-A035-13 | TCGA-B6-A0IE |
| TCGA-BRCA | Primary Tumor | TCGA-B6-A0IG-01A-11R-A035-13 | TCGA-B6-A0IG |
| TCGA-BRCA | Primary Tumor | TCGA-B6-A0IH-01A-11R-A114-13 | TCGA-B6-A0IH |
| TCGA-BRCA | Primary Tumor | TCGA-B6-A0IJ-01A-11R-A035-13 | TCGA-B6-A0IJ |
| TCGA-BRCA | Primary Tumor | TCGA-B6-A0IK-01A-12R-A057-13 | TCGA-B6-A0IK |
| TCGA-BRCA | Primary Tumor | TCGA-B6-A0IM-01A-11R-A035-13 | TCGA-B6-A0IM |
| TCGA-BRCA | Primary Tumor | TCGA-B6-A0IN-01A-11R-A035-13 | TCGA-B6-A0IN |
| TCGA-BRCA | Primary Tumor | TCGA-B6-A0IO-01A-11R-A035-13 | TCGA-B6-A0IO |
| TCGA-BRCA | Primary Tumor | TCGA-B6-A0IP-01A-11R-A035-13 | TCGA-B6-A0IP |
| TCGA-BRCA | Primary Tumor | TCGA-B6-A0IQ-01A-11R-A035-13 | TCGA-B6-A0IQ |
| TCGA-BRCA | Primary Tumor | TCGA-B6-A0RE-01A-11R-A057-13 | TCGA-B6-A0RE |
| TCGA-BRCA | Primary Tumor | TCGA-B6-A0RG-01A-11R-A057-13 | TCGA-B6-A0RG |
| TCGA-BRCA | Primary Tumor | TCGA-B6-A0RH-01A-21R-A114-13 | TCGA-B6-A0RH |
| TCGA-BRCA | Primary Tumor | TCGA-B6-A0RI-01A-11R-A057-13 | TCGA-B6-A0RI |
| TCGA-BRCA | Primary Tumor | TCGA-B6-A0RL-01A-11R-A085-13 | TCGA-B6-A0RL |
| TCGA-BRCA | Primary Tumor | TCGA-B6-A0RM-01A-11R-A085-13 | TCGA-B6-A0RM |
| TCGA-BRCA | Primary Tumor | TCGA-B6-A0RN-01A-12R-A085-13 | TCGA-B6-A0RN |
| TCGA-BRCA | Primary Tumor | TCGA-B6-A0RO-01A-22R-A085-13 | TCGA-B6-A0RO |
| TCGA-BRCA | Primary Tumor | TCGA-B6-A0RP-01A-21R-A085-13 | TCGA-B6-A0RP |
| TCGA-BRCA | Primary Tumor | TCGA-B6-A0RQ-01A-11R-A114-13 | TCGA-B6-A0RQ |
| TCGA-BRCA | Primary Tumor | TCGA-B6-A0RS-01A-11R-A085-13 | TCGA-B6-A0RS |
| TCGA-BRCA | Primary Tumor | TCGA-B6-A0RT-01A-21R-A085-13 | TCGA-B6-A0RT |
| TCGA-BRCA | Primary Tumor | TCGA-B6-A0RU-01A-11R-A085-13 | TCGA-B6-A0RU |
| TCGA-BRCA | Primary Tumor | TCGA-B6-A0RV-01A-11R-A085-13 | TCGA-B6-A0RV |
| TCGA-BRCA | Primary Tumor | TCGA-B6-A0WS-01A-11R-A114-13 | TCGA-B6-A0WS |
| TCGA-BRCA | Primary Tumor | TCGA-B6-A0WT-01A-11R-A108-13 | TCGA-B6-A0WT |
| TCGA-BRCA | Primary Tumor | TCGA-B6-A0WV-01A-11R-A108-13 | TCGA-B6-A0WV |
| TCGA-BRCA | Primary Tumor | TCGA-B6-A0WW-01A-11R-A108-13 | TCGA-B6-A0WW |
| TCGA-BRCA | Primary Tumor | TCGA-B6-A0WX-01A-11R-A108-13 | TCGA-B6-A0WX |
| TCGA-BRCA | Primary Tumor | TCGA-B6-A0WY-01A-11R-A108-13 | TCGA-B6-A0WY |
| TCGA-BRCA | Primary Tumor | TCGA-B6-A0WZ-01A-11R-A108-13 | TCGA-B6-A0WZ |
| TCGA-BRCA | Primary Tumor | TCGA-B6-A0X0-01A-21R-A114-13 | TCGA-B6-A0X0 |
| TCGA-BRCA | Primary Tumor | TCGA-B6-A0X4-01A-11R-A108-13 | TCGA-B6-A0X4 |
| TCGA-BRCA | Primary Tumor | TCGA-B6-A0X5-01A-21R-A108-13 | TCGA-B6-A0X5 |
| TCGA-BRCA | Primary Tumor | TCGA-B6-A0X7-01A-11R-A10I-13 | TCGA-B6-A0X7 |
| TCGA-BRCA | Primary Tumor | TCGA-B6-A1KC-01A-11R-A13P-13 | TCGA-B6-A1KC |

| | | | |
|---|---|---|---|
| TCGA-BRCA | Primary Tumor | TCGA-B6-A1KC-01B-11R-A156-13 | TCGA-B6-A1KC |
| TCGA-BRCA | Primary Tumor | TCGA-B6-A1KF-01A-11R-A13P-13 | TCGA-B6-A1KF |
| TCGA-BRCA | Primary Tumor | TCGA-B6-A1KI-01A-11R-A14L-13 | TCGA-B6-A1KI |
| TCGA-BRCA | Primary Tumor | TCGA-B6-A1KN-01A-11R-A13P-13 | TCGA-B6-A1KN |
| TCGA-BRCA | Primary Tumor | TCGA-B6-A2IU-01A-32R-A18L-13 | TCGA-B6-A2IU |
| TCGA-BRCA | Primary Tumor | TCGA-B6-A3ZX-01A-11R-A23A-13 | TCGA-B6-A3ZX |
| TCGA-BRCA | Primary Tumor | TCGA-B6-A400-01A-11R-A23A-13 | TCGA-B6-A400 |
| TCGA-BRCA | Primary Tumor | TCGA-B6-A401-01A-11R-A23A-13 | TCGA-B6-A401 |
| TCGA-BRCA | Primary Tumor | TCGA-B6-A402-01A-11R-A23A-13 | TCGA-B6-A402 |
| TCGA-BRCA | Primary Tumor | TCGA-B6-A408-01A-12R-A24J-13 | TCGA-B6-A408 |
| TCGA-BRCA | Primary Tumor | TCGA-B6-A409-01A-11R-A24J-13 | TCGA-B6-A409 |
| TCGA-BRCA | Primary Tumor | TCGA-B6-A40B-01A-11R-A23A-13 | TCGA-B6-A40B |
| TCGA-BRCA | Primary Tumor | TCGA-B6-A40C-01A-11R-A23A-13 | TCGA-B6-A40C |
| TCGA-BRCA | Primary Tumor | TCGA-BH-A0AU-01A-11R-A12O-13 | TCGA-BH-A0AU |
| TCGA-BRCA | Primary Tumor | TCGA-BH-A0AV-01A-31R-A114-13 | TCGA-BH-A0AV |
| TCGA-BRCA | Primary Tumor | TCGA-BH-A0AW-01A-11R-A057-13 | TCGA-BH-A0AW |
| TCGA-BRCA | Primary Tumor | TCGA-BH-A0AY-01A-21R-A010-13 | TCGA-BH-A0AY |
| TCGA-BRCA | Primary Tumor | TCGA-BH-A0AZ-01A-21R-A12O-13 | TCGA-BH-A0AZ |
| TCGA-BRCA | Primary Tumor | TCGA-BH-A0B0-01A-21R-A114-13 | TCGA-BH-A0B0 |
| TCGA-BRCA | Primary Tumor | TCGA-BH-A0B1-01A-12R-A057-13 | TCGA-BH-A0B1 |
| TCGA-BRCA | Primary Tumor | TCGA-BH-A0B2-01A-11R-A10I-13 | TCGA-BH-A0B2 |
| TCGA-BRCA | Primary Tumor | TCGA-BH-A0B3-01A-11R-A057-13 | TCGA-BH-A0B3 |
| TCGA-BRCA | Primary Tumor | TCGA-BH-A0B4-01A-11R-A010-13 | TCGA-BH-A0B4 |
| TCGA-BRCA | Primary Tumor | TCGA-BH-A0B5-01A-11R-A12O-13 | TCGA-BH-A0B5 |
| TCGA-BRCA | Primary Tumor | TCGA-BH-A0B6-01A-11R-A19V-13 | TCGA-BH-A0B6 |
| TCGA-BRCA | Primary Tumor | TCGA-BH-A0B7-01A-12R-A114-13 | TCGA-BH-A0B7 |
| TCGA-BRCA | Primary Tumor | TCGA-BH-A0B8-01A-21R-A057-13 | TCGA-BH-A0B8 |
| TCGA-BRCA | Primary Tumor | TCGA-BH-A0B9-01A-11R-A057-13 | TCGA-BH-A0B9 |
| TCGA-BRCA | Primary Tumor | TCGA-BH-A0BA-01A-11R-A057-13 | TCGA-BH-A0BA |
| TCGA-BRCA | Primary Tumor | TCGA-BH-A0BC-01A-22R-A085-13 | TCGA-BH-A0BC |
| TCGA-BRCA | Primary Tumor | TCGA-BH-A0BD-01A-11R-A035-13 | TCGA-BH-A0BD |
| TCGA-BRCA | Primary Tumor | TCGA-BH-A0BF-01A-21R-A12O-13 | TCGA-BH-A0BF |
| TCGA-BRCA | Primary Tumor | TCGA-BH-A0BG-01A-11R-A114-13 | TCGA-BH-A0BG |
| TCGA-BRCA | Primary Tumor | TCGA-BH-A0BJ-01A-11R-A057-13 | TCGA-BH-A0BJ |
| TCGA-BRCA | Primary Tumor | TCGA-BH-A0BL-01A-11R-A114-13 | TCGA-BH-A0BL |
| TCGA-BRCA | Primary Tumor | TCGA-BH-A0BM-01A-11R-A057-13 | TCGA-BH-A0BM |
| TCGA-BRCA | Primary Tumor | TCGA-BH-A0BO-01A-23R-A12C-13 | TCGA-BH-A0BO |
| TCGA-BRCA | Primary Tumor | TCGA-BH-A0BP-01A-11R-A114-13 | TCGA-BH-A0BP |
| TCGA-BRCA | Primary Tumor | TCGA-BH-A0BQ-01A-21R-A114-13 | TCGA-BH-A0BQ |
| TCGA-BRCA | Primary Tumor | TCGA-BH-A0BR-01A-21R-A114-13 | TCGA-BH-A0BR |
| TCGA-BRCA | Primary Tumor | TCGA-BH-A0BS-01A-11R-A12O-13 | TCGA-BH-A0BS |
| TCGA-BRCA | Primary Tumor | TCGA-BH-A0BT-01A-11R-A12O-13 | TCGA-BH-A0BT |
| TCGA-BRCA | Primary Tumor | TCGA-BH-A0BV-01A-11R-A010-13 | TCGA-BH-A0BV |
| TCGA-BRCA | Primary Tumor | TCGA-BH-A0BW-01A-11R-A114-13 | TCGA-BH-A0BW |
| TCGA-BRCA | Primary Tumor | TCGA-BH-A0BZ-01A-31R-A12O-13 | TCGA-BH-A0BZ |
| TCGA-BRCA | Primary Tumor | TCGA-BH-A0C0-01A-21R-A057-13 | TCGA-BH-A0C0 |
| TCGA-BRCA | Primary Tumor | TCGA-BH-A0C1-01B-11R-A12C-13 | TCGA-BH-A0C1 |
| TCGA-BRCA | Primary Tumor | TCGA-BH-A0C3-01A-21R-A12O-13 | TCGA-BH-A0C3 |
| TCGA-BRCA | Primary Tumor | TCGA-BH-A0C7-01B-11R-A114-13 | TCGA-BH-A0C7 |
| TCGA-BRCA | Primary Tumor | TCGA-BH-A0DD-01A-31R-A12O-13 | TCGA-BH-A0DD |
| TCGA-BRCA | Primary Tumor | TCGA-BH-A0DE-01A-11R-A114-13 | TCGA-BH-A0DE |
| TCGA-BRCA | Primary Tumor | TCGA-BH-A0DG-01A-21R-A12O-13 | TCGA-BH-A0DG |
| TCGA-BRCA | Primary Tumor | TCGA-BH-A0DH-01A-11R-A085-13 | TCGA-BH-A0DH |
| TCGA-BRCA | Primary Tumor | TCGA-BH-A0DI-01A-21R-A12O-13 | TCGA-BH-A0DI |
| TCGA-BRCA | Primary Tumor | TCGA-BH-A0DK-01A-21R-A057-13 | TCGA-BH-A0DK |
| TCGA-BRCA | Primary Tumor | TCGA-BH-A0DL-01A-11R-A114-13 | TCGA-BH-A0DL |
| TCGA-BRCA | Primary Tumor | TCGA-BH-A0DO-01B-11R-A12C-13 | TCGA-BH-A0DO |
| TCGA-BRCA | Primary Tumor | TCGA-BH-A0DP-01A-21R-A057-13 | TCGA-BH-A0DP |
| TCGA-BRCA | Primary Tumor | TCGA-BH-A0DQ-01A-11R-A085-13 | TCGA-BH-A0DQ |

| | | | |
|---|---|---|---|
| TCGA-BRCA | Primary Tumor | TCGA-BH-A0DS-01A-11R-A057-13 | TCGA-BH-A0DS |
| TCGA-BRCA | Primary Tumor | TCGA-BH-A0DT-01A-21R-A12C-13 | TCGA-BH-A0DT |
| TCGA-BRCA | Primary Tumor | TCGA-BH-A0DV-01A-21R-A12O-13 | TCGA-BH-A0DV |
| TCGA-BRCA | Primary Tumor | TCGA-BH-A0DX-01A-11R-A114-13 | TCGA-BH-A0DX |
| TCGA-BRCA | Primary Tumor | TCGA-BH-A0DZ-01A-11R-A010-13 | TCGA-BH-A0DZ |
| TCGA-BRCA | Primary Tumor | TCGA-BH-A0E0-01A-11R-A057-13 | TCGA-BH-A0E0 |
| TCGA-BRCA | Primary Tumor | TCGA-BH-A0E1-01A-11R-A057-13 | TCGA-BH-A0E1 |
| TCGA-BRCA | Primary Tumor | TCGA-BH-A0E2-01A-11R-A057-13 | TCGA-BH-A0E2 |
| TCGA-BRCA | Primary Tumor | TCGA-BH-A0E6-01A-11R-A035-13 | TCGA-BH-A0E6 |
| TCGA-BRCA | Primary Tumor | TCGA-BH-A0E7-01A-11R-A035-13 | TCGA-BH-A0E7 |
| TCGA-BRCA | Primary Tumor | TCGA-BH-A0E9-01B-11R-A114-13 | TCGA-BH-A0E9 |
| TCGA-BRCA | Primary Tumor | TCGA-BH-A0EA-01A-11R-A114-13 | TCGA-BH-A0EA |
| TCGA-BRCA | Primary Tumor | TCGA-BH-A0EE-01A-11R-A035-13 | TCGA-BH-A0EE |
| TCGA-BRCA | Primary Tumor | TCGA-BH-A0EI-01A-11R-A114-13 | TCGA-BH-A0EI |
| TCGA-BRCA | Primary Tumor | TCGA-BH-A0GY-01A-11R-A057-13 | TCGA-BH-A0GY |
| TCGA-BRCA | Primary Tumor | TCGA-BH-A0GZ-01A-11R-A057-13 | TCGA-BH-A0GZ |
| TCGA-BRCA | Primary Tumor | TCGA-BH-A0H0-01A-11R-A057-13 | TCGA-BH-A0H0 |
| TCGA-BRCA | Primary Tumor | TCGA-BH-A0H3-01A-11R-A12O-13 | TCGA-BH-A0H3 |
| TCGA-BRCA | Primary Tumor | TCGA-BH-A0H5-01A-21R-A114-13 | TCGA-BH-A0H5 |
| TCGA-BRCA | Primary Tumor | TCGA-BH-A0H6-01A-21R-A057-13 | TCGA-BH-A0H6 |
| TCGA-BRCA | Primary Tumor | TCGA-BH-A0H7-01A-13R-A057-13 | TCGA-BH-A0H7 |
| TCGA-BRCA | Primary Tumor | TCGA-BH-A0H9-01A-11R-A057-13 | TCGA-BH-A0H9 |
| TCGA-BRCA | Primary Tumor | TCGA-BH-A0HA-01A-11R-A12O-13 | TCGA-BH-A0HA |
| TCGA-BRCA | Primary Tumor | TCGA-BH-A0HB-01A-11R-A057-13 | TCGA-BH-A0HB |
| TCGA-BRCA | Primary Tumor | TCGA-BH-A0HF-01A-11R-A057-13 | TCGA-BH-A0HF |
| TCGA-BRCA | Primary Tumor | TCGA-BH-A0HI-01A-11R-A085-13 | TCGA-BH-A0HI |
| TCGA-BRCA | Primary Tumor | TCGA-BH-A0HK-01A-11R-A057-13 | TCGA-BH-A0HK |
| TCGA-BRCA | Primary Tumor | TCGA-BH-A0HL-01A-11R-A10V-13 | TCGA-BH-A0HL |
| TCGA-BRCA | Primary Tumor | TCGA-BH-A0HN-01A-11R-A10V-13 | TCGA-BH-A0HN |
| TCGA-BRCA | Primary Tumor | TCGA-BH-A0HO-01A-11R-A035-13 | TCGA-BH-A0HO |
| TCGA-BRCA | Primary Tumor | TCGA-BH-A0HP-01A-12R-A085-13 | TCGA-BH-A0HP |
| TCGA-BRCA | Primary Tumor | TCGA-BH-A0HQ-01A-11R-A035-13 | TCGA-BH-A0HQ |
| TCGA-BRCA | Primary Tumor | TCGA-BH-A0HU-01A-11R-A035-13 | TCGA-BH-A0HU |
| TCGA-BRCA | Primary Tumor | TCGA-BH-A0HW-01A-11R-A035-13 | TCGA-BH-A0HW |
| TCGA-BRCA | Primary Tumor | TCGA-BH-A0HX-01A-21R-A057-13 | TCGA-BH-A0HX |
| TCGA-BRCA | Primary Tumor | TCGA-BH-A0HY-01A-11R-A057-13 | TCGA-BH-A0HY |
| TCGA-BRCA | Primary Tumor | TCGA-BH-A0RX-01A-21R-A085-13 | TCGA-BH-A0RX |
| TCGA-BRCA | Primary Tumor | TCGA-BH-A0W3-01A-11R-A108-13 | TCGA-BH-A0W3 |
| TCGA-BRCA | Primary Tumor | TCGA-BH-A0W4-01A-11R-A108-13 | TCGA-BH-A0W4 |
| TCGA-BRCA | Primary Tumor | TCGA-BH-A0W5-01A-11R-A108-13 | TCGA-BH-A0W5 |
| TCGA-BRCA | Primary Tumor | TCGA-BH-A0W7-01A-11R-A114-13 | TCGA-BH-A0W7 |
| TCGA-BRCA | Primary Tumor | TCGA-BH-A0WA-01A-11R-A108-13 | TCGA-BH-A0WA |
| TCGA-BRCA | Primary Tumor | TCGA-BH-A18F-01A-11R-A12C-13 | TCGA-BH-A18F |
| TCGA-BRCA | Primary Tumor | TCGA-BH-A18G-01A-11R-A12C-13 | TCGA-BH-A18G |
| TCGA-BRCA | Primary Tumor | TCGA-BH-A18H-01A-11R-A12C-13 | TCGA-BH-A18H |
| TCGA-BRCA | Primary Tumor | TCGA-BH-A18I-01A-11R-A12C-13 | TCGA-BH-A18I |
| TCGA-BRCA | Primary Tumor | TCGA-BH-A18J-01A-11R-A12C-13 | TCGA-BH-A18J |
| TCGA-BRCA | Primary Tumor | TCGA-BH-A18K-01A-11R-A12C-13 | TCGA-BH-A18K |
| TCGA-BRCA | Primary Tumor | TCGA-BH-A18L-01A-32R-A12C-13 | TCGA-BH-A18L |
| TCGA-BRCA | Primary Tumor | TCGA-BH-A18M-01A-11R-A12C-13 | TCGA-BH-A18M |
| TCGA-BRCA | Primary Tumor | TCGA-BH-A18N-01A-11R-A12C-13 | TCGA-BH-A18N |
| TCGA-BRCA | Primary Tumor | TCGA-BH-A18P-01A-11R-A12C-13 | TCGA-BH-A18P |
| TCGA-BRCA | Primary Tumor | TCGA-BH-A18Q-01A-12R-A12C-13 | TCGA-BH-A18Q |
| TCGA-BRCA | Primary Tumor | TCGA-BH-A18R-01A-11R-A12C-13 | TCGA-BH-A18R |
| TCGA-BRCA | Primary Tumor | TCGA-BH-A18S-01A-11R-A12C-13 | TCGA-BH-A18S |
| TCGA-BRCA | Primary Tumor | TCGA-BH-A18T-01A-11R-A12C-13 | TCGA-BH-A18T |
| TCGA-BRCA | Primary Tumor | TCGA-BH-A18U-01A-21R-A12C-13 | TCGA-BH-A18U |
| TCGA-BRCA | Primary Tumor | TCGA-BH-A18V-01A-11R-A12C-13 | TCGA-BH-A18V |
| TCGA-BRCA | Primary Tumor | TCGA-BH-A1EN-01A-11R-A13P-13 | TCGA-BH-A1EN |

| | | | |
|---|---|---|---|
| TCGA-BRCA | Primary Tumor | TCGA-BH-A1EO-01A-11R-A136-13 | TCGA-BH-A1EO |
| TCGA-BRCA | Primary Tumor | TCGA-BH-A1ES-01A-11R-A136-13 | TCGA-BH-A1ES |
| TCGA-BRCA | Primary Tumor | TCGA-BH-A1ET-01A-11R-A136-13 | TCGA-BH-A1ET |
| TCGA-BRCA | Primary Tumor | TCGA-BH-A1EU-01A-11R-A136-13 | TCGA-BH-A1EU |
| TCGA-BRCA | Primary Tumor | TCGA-BH-A1EV-01A-11R-A136-13 | TCGA-BH-A1EV |
| TCGA-BRCA | Primary Tumor | TCGA-BH-A1EW-01A-11R-A136-13 | TCGA-BH-A1EW |
| TCGA-BRCA | Primary Tumor | TCGA-BH-A1EX-01A-11R-A13P-13 | TCGA-BH-A1EX |
| TCGA-BRCA | Primary Tumor | TCGA-BH-A1EY-01A-11R-A13P-13 | TCGA-BH-A1EY |
| TCGA-BRCA | Primary Tumor | TCGA-BH-A1F0-01A-11R-A136-13 | TCGA-BH-A1F0 |
| TCGA-BRCA | Primary Tumor | TCGA-BH-A1F2-01A-31R-A13P-13 | TCGA-BH-A1F2 |
| TCGA-BRCA | Primary Tumor | TCGA-BH-A1F5-01A-12R-A13P-13 | TCGA-BH-A1F5 |
| TCGA-BRCA | Primary Tumor | TCGA-BH-A1F6-01A-11R-A13P-13 | TCGA-BH-A1F6 |
| TCGA-BRCA | Primary Tumor | TCGA-BH-A1F8-01A-11R-A13P-13 | TCGA-BH-A1F8 |
| TCGA-BRCA | Primary Tumor | TCGA-BH-A1FB-01A-11R-A13P-13 | TCGA-BH-A1FB |
| TCGA-BRCA | Primary Tumor | TCGA-BH-A1FC-01A-11R-A13P-13 | TCGA-BH-A1FC |
| TCGA-BRCA | Primary Tumor | TCGA-BH-A1FE-01A-11R-A13P-13 | TCGA-BH-A1FE |
| TCGA-BRCA | Primary Tumor | TCGA-BH-A1FG-01A-11R-A13P-13 | TCGA-BH-A1FG |
| TCGA-BRCA | Primary Tumor | TCGA-BH-A1FH-01A-12R-A13P-13 | TCGA-BH-A1FH |
| TCGA-BRCA | Primary Tumor | TCGA-BH-A1FJ-01A-11R-A13P-13 | TCGA-BH-A1FJ |
| TCGA-BRCA | Primary Tumor | TCGA-BH-A1FL-01A-11R-A13P-13 | TCGA-BH-A1FL |
| TCGA-BRCA | Primary Tumor | TCGA-BH-A1FM-01A-11R-A13P-13 | TCGA-BH-A1FM |
| TCGA-BRCA | Primary Tumor | TCGA-BH-A1FN-01A-11R-A13P-13 | TCGA-BH-A1FN |
| TCGA-BRCA | Primary Tumor | TCGA-BH-A1FR-01A-11R-A13P-13 | TCGA-BH-A1FR |
| TCGA-BRCA | Primary Tumor | TCGA-BH-A1FU-01A-11R-A14C-13 | TCGA-BH-A1FU |
| TCGA-BRCA | Primary Tumor | TCGA-BH-A201-01A-11R-A14L-13 | TCGA-BH-A201 |
| TCGA-BRCA | Primary Tumor | TCGA-BH-A202-01A-11R-A14L-13 | TCGA-BH-A202 |
| TCGA-BRCA | Primary Tumor | TCGA-BH-A203-01A-12R-A168-13 | TCGA-BH-A203 |
| TCGA-BRCA | Primary Tumor | TCGA-BH-A204-01A-11R-A156-13 | TCGA-BH-A204 |
| TCGA-BRCA | Primary Tumor | TCGA-BH-A208-01A-11R-A156-13 | TCGA-BH-A208 |
| TCGA-BRCA | Primary Tumor | TCGA-BH-A209-01A-11R-A156-13 | TCGA-BH-A209 |
| TCGA-BRCA | Primary Tumor | TCGA-BH-A28O-01A-11R-A22I-13 | TCGA-BH-A28O |
| TCGA-BRCA | Primary Tumor | TCGA-BH-A28Q-01A-11R-A16E-13 | TCGA-BH-A28Q |
| TCGA-BRCA | Primary Tumor | TCGA-BH-A2L8-01A-11R-A18L-13 | TCGA-BH-A2L8 |
| TCGA-BRCA | Primary Tumor | TCGA-BH-A42T-01A-11R-A24J-13 | TCGA-BH-A42T |
| TCGA-BRCA | Primary Tumor | TCGA-BH-A42U-01A-12R-A24J-13 | TCGA-BH-A42U |
| TCGA-BRCA | Primary Tumor | TCGA-BH-A42V-01A-11R-A24J-13 | TCGA-BH-A42V |
| TCGA-BRCA | Primary Tumor | TCGA-BH-A5IZ-01A-11R-A27U-13 | TCGA-BH-A5IZ |
| TCGA-BRCA | Primary Tumor | TCGA-BH-A5J0-01A-11R-A27U-13 | TCGA-BH-A5J0 |
| TCGA-BRCA | Primary Tumor | TCGA-BH-A6R8-01A-21R-A33A-13 | TCGA-BH-A6R8 |
| TCGA-BRCA | Primary Tumor | TCGA-BH-A6R9-01A-21R-A32K-13 | TCGA-BH-A6R9 |
| TCGA-BRCA | Primary Tumor | TCGA-BH-A8FY-01A-11R-A36A-13 | TCGA-BH-A8FY |
| TCGA-BRCA | Primary Tumor | TCGA-BH-A8FZ-01A-11R-A358-13 | TCGA-BH-A8FZ |
| TCGA-BRCA | Primary Tumor | TCGA-BH-A8G0-01A-11R-A358-13 | TCGA-BH-A8G0 |
| TCGA-BRCA | Primary Tumor | TCGA-BH-AB28-01A-31R-A41G-13 | TCGA-BH-AB28 |
| TCGA-BRCA | Primary Tumor | TCGA-C8-A12K-01A-21R-A114-13 | TCGA-C8-A12K |
| TCGA-BRCA | Primary Tumor | TCGA-C8-A12L-01A-11R-A114-13 | TCGA-C8-A12L |
| TCGA-BRCA | Primary Tumor | TCGA-C8-A12M-01A-11R-A114-13 | TCGA-C8-A12M |
| TCGA-BRCA | Primary Tumor | TCGA-C8-A12N-01A-11R-A114-13 | TCGA-C8-A12N |
| TCGA-BRCA | Primary Tumor | TCGA-C8-A12O-01A-11R-A114-13 | TCGA-C8-A12O |
| TCGA-BRCA | Primary Tumor | TCGA-C8-A12P-01A-11R-A114-13 | TCGA-C8-A12P |
| TCGA-BRCA | Primary Tumor | TCGA-C8-A12Q-01A-11R-A114-13 | TCGA-C8-A12Q |
| TCGA-BRCA | Primary Tumor | TCGA-C8-A12T-01A-11R-A114-13 | TCGA-C8-A12T |
| TCGA-BRCA | Primary Tumor | TCGA-C8-A12U-01A-11R-A114-13 | TCGA-C8-A12U |
| TCGA-BRCA | Primary Tumor | TCGA-C8-A12V-01A-11R-A114-13 | TCGA-C8-A12V |
| TCGA-BRCA | Primary Tumor | TCGA-C8-A12W-01A-11R-A114-13 | TCGA-C8-A12W |
| TCGA-BRCA | Primary Tumor | TCGA-C8-A12X-01A-11R-A114-13 | TCGA-C8-A12X |
| TCGA-BRCA | Primary Tumor | TCGA-C8-A12Y-01A-11R-A12C-13 | TCGA-C8-A12Y |
| TCGA-BRCA | Primary Tumor | TCGA-C8-A12Z-01A-11R-A114-13 | TCGA-C8-A12Z |
| TCGA-BRCA | Primary Tumor | TCGA-C8-A130-01A-31R-A114-13 | TCGA-C8-A130 |

| | | | |
|---|---|---|---|
| TCGA-BRCA | Primary Tumor | TCGA-C8-A131-01A-11R-A114-13 | TCGA-C8-A131 |
| TCGA-BRCA | Primary Tumor | TCGA-C8-A132-01A-31R-A114-13 | TCGA-C8-A132 |
| TCGA-BRCA | Primary Tumor | TCGA-C8-A133-01A-32R-A12C-13 | TCGA-C8-A133 |
| TCGA-BRCA | Primary Tumor | TCGA-C8-A134-01A-11R-A114-13 | TCGA-C8-A134 |
| TCGA-BRCA | Primary Tumor | TCGA-C8-A135-01A-11R-A114-13 | TCGA-C8-A135 |
| TCGA-BRCA | Primary Tumor | TCGA-C8-A137-01A-11R-A114-13 | TCGA-C8-A137 |
| TCGA-BRCA | Primary Tumor | TCGA-C8-A138-01A-11R-A114-13 | TCGA-C8-A138 |
| TCGA-BRCA | Primary Tumor | TCGA-C8-A1HE-01A-11R-A13P-13 | TCGA-C8-A1HE |
| TCGA-BRCA | Primary Tumor | TCGA-C8-A1HF-01A-11R-A136-13 | TCGA-C8-A1HF |
| TCGA-BRCA | Primary Tumor | TCGA-C8-A1HG-01A-11R-A136-13 | TCGA-C8-A1HG |
| TCGA-BRCA | Primary Tumor | TCGA-C8-A1HI-01A-11R-A136-13 | TCGA-C8-A1HI |
| TCGA-BRCA | Primary Tumor | TCGA-C8-A1HJ-01A-11R-A13P-13 | TCGA-C8-A1HJ |
| TCGA-BRCA | Primary Tumor | TCGA-C8-A1HK-01A-21R-A13P-13 | TCGA-C8-A1HK |
| TCGA-BRCA | Primary Tumor | TCGA-C8-A1HL-01A-11R-A136-13 | TCGA-C8-A1HL |
| TCGA-BRCA | Primary Tumor | TCGA-C8-A1HM-01A-12R-A136-13 | TCGA-C8-A1HM |
| TCGA-BRCA | Primary Tumor | TCGA-C8-A1HN-01A-11R-A136-13 | TCGA-C8-A1HN |
| TCGA-BRCA | Primary Tumor | TCGA-C8-A1HO-01A-11R-A13P-13 | TCGA-C8-A1HO |
| TCGA-BRCA | Primary Tumor | TCGA-C8-A26V-01A-11R-A16E-13 | TCGA-C8-A26V |
| TCGA-BRCA | Primary Tumor | TCGA-C8-A26W-01A-11R-A16E-13 | TCGA-C8-A26W |
| TCGA-BRCA | Primary Tumor | TCGA-C8-A26X-01A-31R-A16E-13 | TCGA-C8-A26X |
| TCGA-BRCA | Primary Tumor | TCGA-C8-A26Y-01A-11R-A16E-13 | TCGA-C8-A26Y |
| TCGA-BRCA | Primary Tumor | TCGA-C8-A26Z-01A-11R-A16E-13 | TCGA-C8-A26Z |
| TCGA-BRCA | Primary Tumor | TCGA-C8-A273-01A-11R-A16E-13 | TCGA-C8-A273 |
| TCGA-BRCA | Primary Tumor | TCGA-C8-A274-01A-11R-A16E-13 | TCGA-C8-A274 |
| TCGA-BRCA | Primary Tumor | TCGA-C8-A275-01A-21R-A16E-13 | TCGA-C8-A275 |
| TCGA-BRCA | Primary Tumor | TCGA-C8-A278-01A-11R-A168-13 | TCGA-C8-A278 |
| TCGA-BRCA | Primary Tumor | TCGA-C8-A27A-01A-11R-A168-13 | TCGA-C8-A27A |
| TCGA-BRCA | Primary Tumor | TCGA-C8-A27B-01A-11R-A168-13 | TCGA-C8-A27B |
| TCGA-BRCA | Primary Tumor | TCGA-C8-A3M7-01A-12R-A21U-13 | TCGA-C8-A3M7 |
| TCGA-BRCA | Primary Tumor | TCGA-C8-A3M8-01A-11R-A214-13 | TCGA-C8-A3M8 |
| TCGA-BRCA | Primary Tumor | TCGA-C8-A8HP-01A-11R-A36A-13 | TCGA-C8-A8HP |
| TCGA-BRCA | Primary Tumor | TCGA-C8-A8HQ-01A-11R-A36A-13 | TCGA-C8-A8HQ |
| TCGA-BRCA | Primary Tumor | TCGA-C8-A8HR-01A-11R-A36A-13 | TCGA-C8-A8HR |
| TCGA-BRCA | Primary Tumor | TCGA-C8-A9FZ-01A-11R-A41G-13 | TCGA-C8-A9FZ |
| TCGA-BRCA | Primary Tumor | TCGA-D8-A13Y-01A-11R-A114-13 | TCGA-D8-A13Y |
| TCGA-BRCA | Primary Tumor | TCGA-D8-A13Z-01A-11R-A114-13 | TCGA-D8-A13Z |
| TCGA-BRCA | Primary Tumor | TCGA-D8-A140-01A-11R-A114-13 | TCGA-D8-A140 |
| TCGA-BRCA | Primary Tumor | TCGA-D8-A141-01A-11R-A114-13 | TCGA-D8-A141 |
| TCGA-BRCA | Primary Tumor | TCGA-D8-A142-01A-11R-A114-13 | TCGA-D8-A142 |
| TCGA-BRCA | Primary Tumor | TCGA-D8-A143-01A-11R-A114-13 | TCGA-D8-A143 |
| TCGA-BRCA | Primary Tumor | TCGA-D8-A145-01A-11R-A114-13 | TCGA-D8-A145 |
| TCGA-BRCA | Primary Tumor | TCGA-D8-A146-01A-31R-A114-13 | TCGA-D8-A146 |
| TCGA-BRCA | Primary Tumor | TCGA-D8-A147-01A-11R-A114-13 | TCGA-D8-A147 |
| TCGA-BRCA | Primary Tumor | TCGA-D8-A1J8-01A-11R-A13P-13 | TCGA-D8-A1J8 |
| TCGA-BRCA | Primary Tumor | TCGA-D8-A1J9-01A-11R-A13P-13 | TCGA-D8-A1J9 |
| TCGA-BRCA | Primary Tumor | TCGA-D8-A1JA-01A-11R-A13P-13 | TCGA-D8-A1JA |
| TCGA-BRCA | Primary Tumor | TCGA-D8-A1JB-01A-11R-A13P-13 | TCGA-D8-A1JB |
| TCGA-BRCA | Primary Tumor | TCGA-D8-A1JC-01A-11R-A13P-13 | TCGA-D8-A1JC |
| TCGA-BRCA | Primary Tumor | TCGA-D8-A1JD-01A-11R-A13P-13 | TCGA-D8-A1JD |
| TCGA-BRCA | Primary Tumor | TCGA-D8-A1JE-01A-11R-A13P-13 | TCGA-D8-A1JE |
| TCGA-BRCA | Primary Tumor | TCGA-D8-A1JF-01A-11R-A13P-13 | TCGA-D8-A1JF |
| TCGA-BRCA | Primary Tumor | TCGA-D8-A1JG-01B-11R-A13P-13 | TCGA-D8-A1JG |
| TCGA-BRCA | Primary Tumor | TCGA-D8-A1JH-01A-11R-A13P-13 | TCGA-D8-A1JH |
| TCGA-BRCA | Primary Tumor | TCGA-D8-A1JI-01A-11R-A13P-13 | TCGA-D8-A1JI |
| TCGA-BRCA | Primary Tumor | TCGA-D8-A1JJ-01A-31R-A14L-13 | TCGA-D8-A1JJ |
| TCGA-BRCA | Primary Tumor | TCGA-D8-A1JK-01A-11R-A13P-13 | TCGA-D8-A1JK |
| TCGA-BRCA | Primary Tumor | TCGA-D8-A1JL-01A-11R-A13P-13 | TCGA-D8-A1JL |
| TCGA-BRCA | Primary Tumor | TCGA-D8-A1JN-01A-11R-A13P-13 | TCGA-D8-A1JN |
| TCGA-BRCA | Primary Tumor | TCGA-D8-A1JP-01A-11R-A13P-13 | TCGA-D8-A1JP |

| | | | |
|---|---|---|---|
| TCGA-BRCA | Primary Tumor | TCGA-D8-A1JS-01A-11R-A13P-13 | TCGA-D8-A1JS |
| TCGA-BRCA | Primary Tumor | TCGA-D8-A1JT-01A-31R-A13P-13 | TCGA-D8-A1JT |
| TCGA-BRCA | Primary Tumor | TCGA-D8-A1JU-01A-11R-A13P-13 | TCGA-D8-A1JU |
| TCGA-BRCA | Primary Tumor | TCGA-D8-A1X5-01A-11R-A14C-13 | TCGA-D8-A1X5 |
| TCGA-BRCA | Primary Tumor | TCGA-D8-A1X6-01A-11R-A14L-13 | TCGA-D8-A1X6 |
| TCGA-BRCA | Primary Tumor | TCGA-D8-A1X7-01A-11R-A14L-13 | TCGA-D8-A1X7 |
| TCGA-BRCA | Primary Tumor | TCGA-D8-A1X8-01A-11R-A14L-13 | TCGA-D8-A1X8 |
| TCGA-BRCA | Primary Tumor | TCGA-D8-A1X9-01A-12R-A156-13 | TCGA-D8-A1X9 |
| TCGA-BRCA | Primary Tumor | TCGA-D8-A1XA-01A-11R-A14C-13 | TCGA-D8-A1XA |
| TCGA-BRCA | Primary Tumor | TCGA-D8-A1XB-01A-11R-A14C-13 | TCGA-D8-A1XB |
| TCGA-BRCA | Primary Tumor | TCGA-D8-A1XC-01A-11R-A14C-13 | TCGA-D8-A1XC |
| TCGA-BRCA | Primary Tumor | TCGA-D8-A1XD-01A-11R-A14C-13 | TCGA-D8-A1XD |
| TCGA-BRCA | Primary Tumor | TCGA-D8-A1XF-01A-11R-A14C-13 | TCGA-D8-A1XF |
| TCGA-BRCA | Primary Tumor | TCGA-D8-A1XG-01A-11R-A14C-13 | TCGA-D8-A1XG |
| TCGA-BRCA | Primary Tumor | TCGA-D8-A1XJ-01A-11R-A14L-13 | TCGA-D8-A1XJ |
| TCGA-BRCA | Primary Tumor | TCGA-D8-A1XK-01A-21R-A14L-13 | TCGA-D8-A1XK |
| TCGA-BRCA | Primary Tumor | TCGA-D8-A1XL-01A-11R-A14L-13 | TCGA-D8-A1XL |
| TCGA-BRCA | Primary Tumor | TCGA-D8-A1XM-01A-21R-A14L-13 | TCGA-D8-A1XM |
| TCGA-BRCA | Primary Tumor | TCGA-D8-A1XO-01A-11R-A14L-13 | TCGA-D8-A1XO |
| TCGA-BRCA | Primary Tumor | TCGA-D8-A1XQ-01A-11R-A14L-13 | TCGA-D8-A1XQ |
| TCGA-BRCA | Primary Tumor | TCGA-D8-A1XR-01A-11R-A14L-13 | TCGA-D8-A1XR |
| TCGA-BRCA | Primary Tumor | TCGA-D8-A1XS-01A-11R-A14L-13 | TCGA-D8-A1XS |
| TCGA-BRCA | Primary Tumor | TCGA-D8-A1XT-01A-11R-A14L-13 | TCGA-D8-A1XT |
| TCGA-BRCA | Primary Tumor | TCGA-D8-A1XU-01A-11R-A14L-13 | TCGA-D8-A1XU |
| TCGA-BRCA | Primary Tumor | TCGA-D8-A1XV-01A-11R-A14L-13 | TCGA-D8-A1XV |
| TCGA-BRCA | Primary Tumor | TCGA-D8-A1XW-01A-11R-A14L-13 | TCGA-D8-A1XW |
| TCGA-BRCA | Primary Tumor | TCGA-D8-A1XY-01A-11R-A14L-13 | TCGA-D8-A1XY |
| TCGA-BRCA | Primary Tumor | TCGA-D8-A1XZ-01A-11R-A14L-13 | TCGA-D8-A1XZ |
| TCGA-BRCA | Primary Tumor | TCGA-D8-A1Y0-01A-11R-A14L-13 | TCGA-D8-A1Y0 |
| TCGA-BRCA | Primary Tumor | TCGA-D8-A1Y1-01A-21R-A14L-13 | TCGA-D8-A1Y1 |
| TCGA-BRCA | Primary Tumor | TCGA-D8-A1Y2-01A-11R-A156-13 | TCGA-D8-A1Y2 |
| TCGA-BRCA | Primary Tumor | TCGA-D8-A1Y3-01A-11R-A156-13 | TCGA-D8-A1Y3 |
| TCGA-BRCA | Primary Tumor | TCGA-D8-A27E-01A-11R-A16E-13 | TCGA-D8-A27E |
| TCGA-BRCA | Primary Tumor | TCGA-D8-A27F-01A-11R-A16E-13 | TCGA-D8-A27F |
| TCGA-BRCA | Primary Tumor | TCGA-D8-A27G-01A-11R-A16E-13 | TCGA-D8-A27G |
| TCGA-BRCA | Primary Tumor | TCGA-D8-A27H-01A-11R-A16E-13 | TCGA-D8-A27H |
| TCGA-BRCA | Primary Tumor | TCGA-D8-A27I-01A-11R-A16E-13 | TCGA-D8-A27I |
| TCGA-BRCA | Primary Tumor | TCGA-D8-A27K-01A-11R-A16E-13 | TCGA-D8-A27K |
| TCGA-BRCA | Primary Tumor | TCGA-D8-A27L-01A-11R-A16E-13 | TCGA-D8-A27L |
| TCGA-BRCA | Primary Tumor | TCGA-D8-A27M-01A-11R-A16E-13 | TCGA-D8-A27M |
| TCGA-BRCA | Primary Tumor | TCGA-D8-A27N-01A-11R-A16E-13 | TCGA-D8-A27N |
| TCGA-BRCA | Primary Tumor | TCGA-D8-A27P-01A-11R-A16E-13 | TCGA-D8-A27P |
| TCGA-BRCA | Primary Tumor | TCGA-D8-A27R-01A-11R-A16E-13 | TCGA-D8-A27R |
| TCGA-BRCA | Primary Tumor | TCGA-D8-A27T-01A-11R-A16E-13 | TCGA-D8-A27T |
| TCGA-BRCA | Primary Tumor | TCGA-D8-A27V-01A-12R-A17A-13 | TCGA-D8-A27V |
| TCGA-BRCA | Primary Tumor | TCGA-D8-A27W-01A-11R-A16E-13 | TCGA-D8-A27W |
| TCGA-BRCA | Primary Tumor | TCGA-D8-A3Z5-01A-41R-A24J-13 | TCGA-D8-A3Z5 |
| TCGA-BRCA | Primary Tumor | TCGA-D8-A3Z6-01A-11R-A23A-13 | TCGA-D8-A3Z6 |
| TCGA-BRCA | Primary Tumor | TCGA-D8-A4Z1-01A-21R-A25Z-13 | TCGA-D8-A4Z1 |
| TCGA-BRCA | Primary Tumor | TCGA-D8-A73U-01A-11R-A33A-13 | TCGA-D8-A73U |
| TCGA-BRCA | Primary Tumor | TCGA-D8-A73W-01A-22R-A358-13 | TCGA-D8-A73W |
| TCGA-BRCA | Primary Tumor | TCGA-D8-A73X-01A-11R-A32K-13 | TCGA-D8-A73X |
| TCGA-BRCA | Primary Tumor | TCGA-E2-A105-01A-11R-A10I-13 | TCGA-E2-A105 |
| TCGA-BRCA | Primary Tumor | TCGA-E2-A106-01A-11R-A10I-13 | TCGA-E2-A106 |
| TCGA-BRCA | Primary Tumor | TCGA-E2-A107-01A-11R-A10I-13 | TCGA-E2-A107 |
| TCGA-BRCA | Primary Tumor | TCGA-E2-A108-01A-13R-A10I-13 | TCGA-E2-A108 |
| TCGA-BRCA | Primary Tumor | TCGA-E2-A109-01A-11R-A10I-13 | TCGA-E2-A109 |
| TCGA-BRCA | Primary Tumor | TCGA-E2-A10A-01A-21R-A114-13 | TCGA-E2-A10A |
| TCGA-BRCA | Primary Tumor | TCGA-E2-A10B-01A-11R-A10I-13 | TCGA-E2-A10B |

| | | | |
|---|---|---|---|
| TCGA-BRCA | Primary Tumor | TCGA-E2-A10C-01A-21R-A10I-13 | TCGA-E2-A10C |
| TCGA-BRCA | Primary Tumor | TCGA-E2-A10E-01A-21R-A10I-13 | TCGA-E2-A10E |
| TCGA-BRCA | Primary Tumor | TCGA-E2-A10F-01A-11R-A10I-13 | TCGA-E2-A10F |
| TCGA-BRCA | Primary Tumor | TCGA-E2-A14N-01A-31R-A136-13 | TCGA-E2-A14N |
| TCGA-BRCA | Primary Tumor | TCGA-E2-A14O-01A-31R-A114-13 | TCGA-E2-A14O |
| TCGA-BRCA | Primary Tumor | TCGA-E2-A14P-01A-31R-A12C-13 | TCGA-E2-A14P |
| TCGA-BRCA | Primary Tumor | TCGA-E2-A14Q-01A-11R-A12C-13 | TCGA-E2-A14Q |
| TCGA-BRCA | Primary Tumor | TCGA-E2-A14R-01A-11R-A114-13 | TCGA-E2-A14R |
| TCGA-BRCA | Primary Tumor | TCGA-E2-A14S-01A-11R-A12C-13 | TCGA-E2-A14S |
| TCGA-BRCA | Primary Tumor | TCGA-E2-A14T-01A-11R-A114-13 | TCGA-E2-A14T |
| TCGA-BRCA | Primary Tumor | TCGA-E2-A14U-01A-11R-A22I-13 | TCGA-E2-A14U |
| TCGA-BRCA | Primary Tumor | TCGA-E2-A14V-01A-11R-A12C-13 | TCGA-E2-A14V |
| TCGA-BRCA | Primary Tumor | TCGA-E2-A14W-01A-11R-A12C-13 | TCGA-E2-A14W |
| TCGA-BRCA | Primary Tumor | TCGA-E2-A14X-01A-11R-A114-13 | TCGA-E2-A14X |
| TCGA-BRCA | Primary Tumor | TCGA-E2-A14Y-01A-21R-A12C-13 | TCGA-E2-A14Y |
| TCGA-BRCA | Primary Tumor | TCGA-E2-A14Z-01A-11R-A114-13 | TCGA-E2-A14Z |
| TCGA-BRCA | Primary Tumor | TCGA-E2-A150-01A-11R-A12C-13 | TCGA-E2-A150 |
| TCGA-BRCA | Primary Tumor | TCGA-E2-A152-01A-11R-A12C-13 | TCGA-E2-A152 |
| TCGA-BRCA | Primary Tumor | TCGA-E2-A153-01A-12R-A12C-13 | TCGA-E2-A153 |
| TCGA-BRCA | Primary Tumor | TCGA-E2-A154-01A-11R-A114-13 | TCGA-E2-A154 |
| TCGA-BRCA | Primary Tumor | TCGA-E2-A155-01A-11R-A12C-13 | TCGA-E2-A155 |
| TCGA-BRCA | Primary Tumor | TCGA-E2-A156-01A-11R-A12C-13 | TCGA-E2-A156 |
| TCGA-BRCA | Primary Tumor | TCGA-E2-A158-01A-11R-A12C-13 | TCGA-E2-A158 |
| TCGA-BRCA | Primary Tumor | TCGA-E2-A159-01A-11R-A114-13 | TCGA-E2-A159 |
| TCGA-BRCA | Primary Tumor | TCGA-E2-A15A-01A-11R-A12C-13 | TCGA-E2-A15A |
| TCGA-BRCA | Primary Tumor | TCGA-E2-A15C-01A-31R-A12C-13 | TCGA-E2-A15C |
| TCGA-BRCA | Primary Tumor | TCGA-E2-A15D-01A-11R-A114-13 | TCGA-E2-A15D |
| TCGA-BRCA | Primary Tumor | TCGA-E2-A15E-01A-11R-A12C-13 | TCGA-E2-A15E |
| TCGA-BRCA | Primary Tumor | TCGA-E2-A15F-01A-11R-A114-13 | TCGA-E2-A15F |
| TCGA-BRCA | Primary Tumor | TCGA-E2-A15G-01A-11R-A12C-13 | TCGA-E2-A15G |
| TCGA-BRCA | Primary Tumor | TCGA-E2-A15H-01A-11R-A12C-13 | TCGA-E2-A15H |
| TCGA-BRCA | Primary Tumor | TCGA-E2-A15I-01A-21R-A136-13 | TCGA-E2-A15I |
| TCGA-BRCA | Primary Tumor | TCGA-E2-A15J-01A-11R-A12O-13 | TCGA-E2-A15J |
| TCGA-BRCA | Primary Tumor | TCGA-E2-A15K-01A-11R-A12O-13 | TCGA-E2-A15K |
| TCGA-BRCA | Primary Tumor | TCGA-E2-A15L-01A-11R-A12C-13 | TCGA-E2-A15L |
| TCGA-BRCA | Primary Tumor | TCGA-E2-A15M-01A-11R-A12C-13 | TCGA-E2-A15M |
| TCGA-BRCA | Primary Tumor | TCGA-E2-A15O-01A-11R-A114-13 | TCGA-E2-A15O |
| TCGA-BRCA | Primary Tumor | TCGA-E2-A15P-01A-11R-A114-13 | TCGA-E2-A15P |
| TCGA-BRCA | Primary Tumor | TCGA-E2-A15R-01A-11R-A114-13 | TCGA-E2-A15R |
| TCGA-BRCA | Primary Tumor | TCGA-E2-A15S-01A-11R-A114-13 | TCGA-E2-A15S |
| TCGA-BRCA | Primary Tumor | TCGA-E2-A15T-01A-11R-A114-13 | TCGA-E2-A15T |
| TCGA-BRCA | Primary Tumor | TCGA-E2-A1AZ-01A-11R-A12O-13 | TCGA-E2-A1AZ |
| TCGA-BRCA | Primary Tumor | TCGA-E2-A1B0-01A-11R-A12O-13 | TCGA-E2-A1B0 |
| TCGA-BRCA | Primary Tumor | TCGA-E2-A1B1-01A-21R-A12O-13 | TCGA-E2-A1B1 |
| TCGA-BRCA | Primary Tumor | TCGA-E2-A1B4-01A-11R-A12O-13 | TCGA-E2-A1B4 |
| TCGA-BRCA | Primary Tumor | TCGA-E2-A1B5-01A-21R-A12O-13 | TCGA-E2-A1B5 |
| TCGA-BRCA | Primary Tumor | TCGA-E2-A1B6-01A-31R-A12O-13 | TCGA-E2-A1B6 |
| TCGA-BRCA | Primary Tumor | TCGA-E2-A1BC-01A-11R-A12O-13 | TCGA-E2-A1BC |
| TCGA-BRCA | Primary Tumor | TCGA-E2-A1BD-01A-11R-A12O-13 | TCGA-E2-A1BD |
| TCGA-BRCA | Primary Tumor | TCGA-E2-A1IE-01A-11R-A13P-13 | TCGA-E2-A1IE |
| TCGA-BRCA | Primary Tumor | TCGA-E2-A1IF-01A-11R-A143-13 | TCGA-E2-A1IF |
| TCGA-BRCA | Primary Tumor | TCGA-E2-A1IG-01A-11R-A143-13 | TCGA-E2-A1IG |
| TCGA-BRCA | Primary Tumor | TCGA-E2-A1IH-01A-11R-A13P-13 | TCGA-E2-A1IH |
| TCGA-BRCA | Primary Tumor | TCGA-E2-A1II-01A-11R-A143-13 | TCGA-E2-A1II |
| TCGA-BRCA | Primary Tumor | TCGA-E2-A1IJ-01A-11R-A143-13 | TCGA-E2-A1IJ |
| TCGA-BRCA | Primary Tumor | TCGA-E2-A1IK-01A-11R-A143-13 | TCGA-E2-A1IK |
| TCGA-BRCA | Primary Tumor | TCGA-E2-A1IL-01A-11R-A14C-13 | TCGA-E2-A1IL |
| TCGA-BRCA | Primary Tumor | TCGA-E2-A1IN-01A-11R-A13P-13 | TCGA-E2-A1IN |
| TCGA-BRCA | Primary Tumor | TCGA-E2-A1IO-01A-11R-A143-13 | TCGA-E2-A1IO |

| | | | |
|---|---|---|---|
| TCGA-BRCA | Primary Tumor | TCGA-E2-A1IU-01A-11R-A14C-13 | TCGA-E2-A1IU |
| TCGA-BRCA | Primary Tumor | TCGA-E2-A1L6-01A-11R-A13P-13 | TCGA-E2-A1L6 |
| TCGA-BRCA | Primary Tumor | TCGA-E2-A1L7-01A-11R-A143-13 | TCGA-E2-A1L7 |
| TCGA-BRCA | Primary Tumor | TCGA-E2-A1L8-01A-11R-A13P-13 | TCGA-E2-A1L8 |
| TCGA-BRCA | Primary Tumor | TCGA-E2-A1L9-01A-11R-A13P-13 | TCGA-E2-A1L9 |
| TCGA-BRCA | Primary Tumor | TCGA-E2-A1LA-01A-11R-A143-13 | TCGA-E2-A1LA |
| TCGA-BRCA | Primary Tumor | TCGA-E2-A1LB-01A-11R-A143-13 | TCGA-E2-A1LB |
| TCGA-BRCA | Primary Tumor | TCGA-E2-A1LE-01A-12R-A19V-13 | TCGA-E2-A1LE |
| TCGA-BRCA | Primary Tumor | TCGA-E2-A1LG-01A-21R-A14L-13 | TCGA-E2-A1LG |
| TCGA-BRCA | Primary Tumor | TCGA-E2-A1LH-01A-11R-A14C-13 | TCGA-E2-A1LH |
| TCGA-BRCA | Primary Tumor | TCGA-E2-A1LI-01A-12R-A156-13 | TCGA-E2-A1LI |
| TCGA-BRCA | Primary Tumor | TCGA-E2-A1LK-01A-21R-A14C-13 | TCGA-E2-A1LK |
| TCGA-BRCA | Primary Tumor | TCGA-E2-A1LL-01A-11R-A143-13 | TCGA-E2-A1LL |
| TCGA-BRCA | Primary Tumor | TCGA-E2-A1LS-01A-12R-A156-13 | TCGA-E2-A1LS |
| TCGA-BRCA | Primary Tumor | TCGA-E2-A2P5-01A-11R-A19V-13 | TCGA-E2-A2P5 |
| TCGA-BRCA | Primary Tumor | TCGA-E2-A2P6-01A-11R-A19V-13 | TCGA-E2-A2P6 |
| TCGA-BRCA | Primary Tumor | TCGA-E2-A3DX-01A-21R-A214-13 | TCGA-E2-A3DX |
| TCGA-BRCA | Primary Tumor | TCGA-E2-A56Z-01A-12R-A29V-13 | TCGA-E2-A56Z |
| TCGA-BRCA | Primary Tumor | TCGA-E2-A570-01A-11R-A29V-13 | TCGA-E2-A570 |
| TCGA-BRCA | Primary Tumor | TCGA-E2-A572-01A-13R-A31S-13 | TCGA-E2-A572 |
| TCGA-BRCA | Primary Tumor | TCGA-E2-A573-01A-11R-A29V-13 | TCGA-E2-A573 |
| TCGA-BRCA | Primary Tumor | TCGA-E2-A574-01A-11R-A29V-13 | TCGA-E2-A574 |
| TCGA-BRCA | Primary Tumor | TCGA-E2-A576-01A-11R-A31S-13 | TCGA-E2-A576 |
| TCGA-BRCA | Primary Tumor | TCGA-E2-A9RU-01A-11R-A41G-13 | TCGA-E2-A9RU |
| TCGA-BRCA | Primary Tumor | TCGA-E9-A1N3-01A-12R-A156-13 | TCGA-E9-A1N3 |
| TCGA-BRCA | Primary Tumor | TCGA-E9-A1N4-01A-11R-A14L-13 | TCGA-E9-A1N4 |
| TCGA-BRCA | Primary Tumor | TCGA-E9-A1N5-01A-11R-A14C-13 | TCGA-E9-A1N5 |
| TCGA-BRCA | Primary Tumor | TCGA-E9-A1N6-01A-11R-A143-13 | TCGA-E9-A1N6 |
| TCGA-BRCA | Primary Tumor | TCGA-E9-A1N8-01A-11R-A143-13 | TCGA-E9-A1N8 |
| TCGA-BRCA | Primary Tumor | TCGA-E9-A1N9-01A-11R-A14C-13 | TCGA-E9-A1N9 |
| TCGA-BRCA | Primary Tumor | TCGA-E9-A1NA-01A-11R-A143-13 | TCGA-E9-A1NA |
| TCGA-BRCA | Primary Tumor | TCGA-E9-A1ND-01A-11R-A143-13 | TCGA-E9-A1ND |
| TCGA-BRCA | Primary Tumor | TCGA-E9-A1NE-01A-21R-A14L-13 | TCGA-E9-A1NE |
| TCGA-BRCA | Primary Tumor | TCGA-E9-A1NF-01A-11R-A14C-13 | TCGA-E9-A1NF |
| TCGA-BRCA | Primary Tumor | TCGA-E9-A1NG-01A-21R-A14L-13 | TCGA-E9-A1NG |
| TCGA-BRCA | Primary Tumor | TCGA-E9-A1NH-01A-11R-A14C-13 | TCGA-E9-A1NH |
| TCGA-BRCA | Primary Tumor | TCGA-E9-A1NI-01A-11R-A14C-13 | TCGA-E9-A1NI |
| TCGA-BRCA | Primary Tumor | TCGA-E9-A1QZ-01A-21R-A168-13 | TCGA-E9-A1QZ |
| TCGA-BRCA | Primary Tumor | TCGA-E9-A1R0-01A-22R-A16E-13 | TCGA-E9-A1R0 |
| TCGA-BRCA | Primary Tumor | TCGA-E9-A1R2-01A-11R-A14C-13 | TCGA-E9-A1R2 |
| TCGA-BRCA | Primary Tumor | TCGA-E9-A1R3-01A-31R-A14L-13 | TCGA-E9-A1R3 |
| TCGA-BRCA | Primary Tumor | TCGA-E9-A1R4-01A-21R-A14C-13 | TCGA-E9-A1R4 |
| TCGA-BRCA | Primary Tumor | TCGA-E9-A1R5-01A-11R-A14L-13 | TCGA-E9-A1R5 |
| TCGA-BRCA | Primary Tumor | TCGA-E9-A1R6-01A-11R-A14C-13 | TCGA-E9-A1R6 |
| TCGA-BRCA | Primary Tumor | TCGA-E9-A1R7-01A-11R-A14L-13 | TCGA-E9-A1R7 |
| TCGA-BRCA | Primary Tumor | TCGA-E9-A1RA-01A-11R-A14C-13 | TCGA-E9-A1RA |
| TCGA-BRCA | Primary Tumor | TCGA-E9-A1RB-01A-11R-A156-13 | TCGA-E9-A1RB |
| TCGA-BRCA | Primary Tumor | TCGA-E9-A1RC-01A-11R-A156-13 | TCGA-E9-A1RC |
| TCGA-BRCA | Primary Tumor | TCGA-E9-A1RD-01A-11R-A156-13 | TCGA-E9-A1RD |
| TCGA-BRCA | Primary Tumor | TCGA-E9-A1RE-01A-11R-A156-13 | TCGA-E9-A1RE |
| TCGA-BRCA | Primary Tumor | TCGA-E9-A1RF-01A-11R-A156-13 | TCGA-E9-A1RF |
| TCGA-BRCA | Primary Tumor | TCGA-E9-A1RG-01A-11R-A14C-13 | TCGA-E9-A1RG |
| TCGA-BRCA | Primary Tumor | TCGA-E9-A1RH-01A-21R-A168-13 | TCGA-E9-A1RH |
| TCGA-BRCA | Primary Tumor | TCGA-E9-A1RI-01A-11R-A168-13 | TCGA-E9-A1RI |
| TCGA-BRCA | Primary Tumor | TCGA-E9-A226-01A-21R-A156-13 | TCGA-E9-A226 |
| TCGA-BRCA | Primary Tumor | TCGA-E9-A227-01A-11R-A156-13 | TCGA-E9-A227 |
| TCGA-BRCA | Primary Tumor | TCGA-E9-A228-01A-31R-A156-13 | TCGA-E9-A228 |
| TCGA-BRCA | Primary Tumor | TCGA-E9-A229-01A-31R-A156-13 | TCGA-E9-A229 |
| TCGA-BRCA | Primary Tumor | TCGA-E9-A22A-01A-11R-A156-13 | TCGA-E9-A22A |

| | | | |
|---|---|---|---|
| TCGA-BRCA | Primary Tumor | TCGA-E9-A22B-01A-11R-A156-13 | TCGA-E9-A22B |
| TCGA-BRCA | Primary Tumor | TCGA-E9-A22D-01A-11R-A156-13 | TCGA-E9-A22D |
| TCGA-BRCA | Primary Tumor | TCGA-E9-A22E-01A-11R-A156-13 | TCGA-E9-A22E |
| TCGA-BRCA | Primary Tumor | TCGA-E9-A22G-01A-11R-A156-13 | TCGA-E9-A22G |
| TCGA-BRCA | Primary Tumor | TCGA-E9-A22H-01A-11R-A156-13 | TCGA-E9-A22H |
| TCGA-BRCA | Primary Tumor | TCGA-E9-A243-01A-21R-A168-13 | TCGA-E9-A243 |
| TCGA-BRCA | Primary Tumor | TCGA-E9-A244-01A-11R-A168-13 | TCGA-E9-A244 |
| TCGA-BRCA | Primary Tumor | TCGA-E9-A245-01A-22R-A16E-13 | TCGA-E9-A245 |
| TCGA-BRCA | Primary Tumor | TCGA-E9-A247-01A-11R-A168-13 | TCGA-E9-A247 |
| TCGA-BRCA | Primary Tumor | TCGA-E9-A248-01A-11R-A168-13 | TCGA-E9-A248 |
| TCGA-BRCA | Primary Tumor | TCGA-E9-A249-01A-11R-A168-13 | TCGA-E9-A249 |
| TCGA-BRCA | Primary Tumor | TCGA-E9-A24A-01A-11R-A168-13 | TCGA-E9-A24A |
| TCGA-BRCA | Primary Tumor | TCGA-E9-A295-01A-11R-A16E-13 | TCGA-E9-A295 |
| TCGA-BRCA | Primary Tumor | TCGA-E9-A2JS-01A-11R-A17X-13 | TCGA-E9-A2JS |
| TCGA-BRCA | Primary Tumor | TCGA-E9-A2JT-01A-22R-A18L-13 | TCGA-E9-A2JT |
| TCGA-BRCA | Primary Tumor | TCGA-E9-A3Q9-01A-11R-A21U-13 | TCGA-E9-A3Q9 |
| TCGA-BRCA | Primary Tumor | TCGA-E9-A3QA-01A-61R-A22I-13 | TCGA-E9-A3QA |
| TCGA-BRCA | Primary Tumor | TCGA-E9-A3X8-01A-31R-A22V-13 | TCGA-E9-A3X8 |
| TCGA-BRCA | Primary Tumor | TCGA-E9-A54X-01A-11R-A25Z-13 | TCGA-E9-A54X |
| TCGA-BRCA | Primary Tumor | TCGA-E9-A54Y-01A-11R-A25Z-13 | TCGA-E9-A54Y |
| TCGA-BRCA | Primary Tumor | TCGA-E9-A5FK-01A-11R-A27U-13 | TCGA-E9-A5FK |
| TCGA-BRCA | Primary Tumor | TCGA-E9-A5FL-01A-11R-A27U-13 | TCGA-E9-A5FL |
| TCGA-BRCA | Primary Tumor | TCGA-E9-A5UO-01A-11R-A28I-13 | TCGA-E9-A5UO |
| TCGA-BRCA | Primary Tumor | TCGA-E9-A5UP-01A-11R-A28I-13 | TCGA-E9-A5UP |
| TCGA-BRCA | Primary Tumor | TCGA-E9-A6HE-01A-11R-A31S-13 | TCGA-E9-A6HE |
| TCGA-BRCA | Primary Tumor | TCGA-EW-A1IW-01A-11R-A13P-13 | TCGA-EW-A1IW |
| TCGA-BRCA | Primary Tumor | TCGA-EW-A1IX-01A-12R-A143-13 | TCGA-EW-A1IX |
| TCGA-BRCA | Primary Tumor | TCGA-EW-A1IY-01A-11R-A13P-13 | TCGA-EW-A1IY |
| TCGA-BRCA | Primary Tumor | TCGA-EW-A1IZ-01A-11R-A13P-13 | TCGA-EW-A1IZ |
| TCGA-BRCA | Primary Tumor | TCGA-EW-A1J1-01A-11R-A13P-13 | TCGA-EW-A1J1 |
| TCGA-BRCA | Primary Tumor | TCGA-EW-A1J2-01A-21R-A13P-13 | TCGA-EW-A1J2 |
| TCGA-BRCA | Primary Tumor | TCGA-EW-A1J3-01A-11R-A13P-13 | TCGA-EW-A1J3 |
| TCGA-BRCA | Primary Tumor | TCGA-EW-A1J5-01A-11R-A13P-13 | TCGA-EW-A1J5 |
| TCGA-BRCA | Primary Tumor | TCGA-EW-A1J6-01A-11R-A13P-13 | TCGA-EW-A1J6 |
| TCGA-BRCA | Primary Tumor | TCGA-EW-A1OV-01A-11R-A143-13 | TCGA-EW-A1OV |
| TCGA-BRCA | Primary Tumor | TCGA-EW-A1OW-01A-21R-A143-13 | TCGA-EW-A1OW |
| TCGA-BRCA | Primary Tumor | TCGA-EW-A1OX-01A-11R-A143-13 | TCGA-EW-A1OX |
| TCGA-BRCA | Primary Tumor | TCGA-EW-A1OY-01A-11R-A143-13 | TCGA-EW-A1OY |
| TCGA-BRCA | Primary Tumor | TCGA-EW-A1OZ-01A-11R-A143-13 | TCGA-EW-A1OZ |
| TCGA-BRCA | Primary Tumor | TCGA-EW-A1P0-01A-11R-A143-13 | TCGA-EW-A1P0 |
| TCGA-BRCA | Primary Tumor | TCGA-EW-A1P1-01A-31R-A14C-13 | TCGA-EW-A1P1 |
| TCGA-BRCA | Primary Tumor | TCGA-EW-A1P3-01A-11R-A143-13 | TCGA-EW-A1P3 |
| TCGA-BRCA | Primary Tumor | TCGA-EW-A1P4-01A-21R-A143-13 | TCGA-EW-A1P4 |
| TCGA-BRCA | Primary Tumor | TCGA-EW-A1P5-01A-11R-A143-13 | TCGA-EW-A1P5 |
| TCGA-BRCA | Primary Tumor | TCGA-EW-A1P6-01A-11R-A143-13 | TCGA-EW-A1P6 |
| TCGA-BRCA | Primary Tumor | TCGA-EW-A1P7-01A-21R-A143-13 | TCGA-EW-A1P7 |
| TCGA-BRCA | Primary Tumor | TCGA-EW-A1P8-01A-11R-A143-13 | TCGA-EW-A1P8 |
| TCGA-BRCA | Primary Tumor | TCGA-EW-A1PA-01A-11R-A143-13 | TCGA-EW-A1PA |
| TCGA-BRCA | Primary Tumor | TCGA-EW-A1PB-01A-11R-A143-13 | TCGA-EW-A1PB |
| TCGA-BRCA | Primary Tumor | TCGA-EW-A1PC-01B-11R-A21U-13 | TCGA-EW-A1PC |
| TCGA-BRCA | Primary Tumor | TCGA-EW-A1PD-01A-11R-A143-13 | TCGA-EW-A1PD |
| TCGA-BRCA | Primary Tumor | TCGA-EW-A1PE-01A-11R-A143-13 | TCGA-EW-A1PE |
| TCGA-BRCA | Primary Tumor | TCGA-EW-A1PF-01A-11R-A143-13 | TCGA-EW-A1PF |
| TCGA-BRCA | Primary Tumor | TCGA-EW-A1PG-01A-11R-A143-13 | TCGA-EW-A1PG |
| TCGA-BRCA | Primary Tumor | TCGA-EW-A1PH-01A-11R-A14L-13 | TCGA-EW-A1PH |
| TCGA-BRCA | Primary Tumor | TCGA-EW-A2FR-01A-11R-A21U-13 | TCGA-EW-A2FR |
| TCGA-BRCA | Primary Tumor | TCGA-EW-A2FS-01A-11R-A17A-13 | TCGA-EW-A2FS |
| TCGA-BRCA | Primary Tumor | TCGA-EW-A2FV-01A-11R-A17A-13 | TCGA-EW-A2FV |
| TCGA-BRCA | Primary Tumor | TCGA-EW-A2FW-01A-11R-A17A-13 | TCGA-EW-A2FW |

| | | | |
|---|---|---|---|
| TCGA-BRCA | Primary Tumor | TCGA-EW-A3E8-01B-11R-A24J-13 | TCGA-EW-A3E8 |
| TCGA-BRCA | Primary Tumor | TCGA-EW-A3U0-01A-11R-A22I-13 | TCGA-EW-A3U0 |
| TCGA-BRCA | Primary Tumor | TCGA-EW-A423-01A-11R-A24J-13 | TCGA-EW-A423 |
| TCGA-BRCA | Primary Tumor | TCGA-EW-A424-01A-11R-A24J-13 | TCGA-EW-A424 |
| TCGA-BRCA | Primary Tumor | TCGA-EW-A6S9-01A-22R-A33A-13 | TCGA-EW-A6S9 |
| TCGA-BRCA | Primary Tumor | TCGA-EW-A6SA-01A-21R-A32K-13 | TCGA-EW-A6SA |
| TCGA-BRCA | Primary Tumor | TCGA-EW-A6SB-01A-12R-A32K-13 | TCGA-EW-A6SB |
| TCGA-BRCA | Primary Tumor | TCGA-EW-A6SC-01A-12R-A32K-13 | TCGA-EW-A6SC |
| TCGA-BRCA | Primary Tumor | TCGA-EW-A6SD-01A-12R-A33A-13 | TCGA-EW-A6SD |
| TCGA-BRCA | Primary Tumor | TCGA-GI-A2C8-01A-11R-A16E-13 | TCGA-GI-A2C8 |
| TCGA-BRCA | Primary Tumor | TCGA-GI-A2C9-01A-11R-A21U-13 | TCGA-GI-A2C9 |
| TCGA-BRCA | Primary Tumor | TCGA-GM-A2D9-01A-11R-A18L-13 | TCGA-GM-A2D9 |
| TCGA-BRCA | Primary Tumor | TCGA-GM-A2DA-01A-11R-A18L-13 | TCGA-GM-A2DA |
| TCGA-BRCA | Primary Tumor | TCGA-GM-A2DB-01A-31R-A18L-13 | TCGA-GM-A2DB |
| TCGA-BRCA | Primary Tumor | TCGA-GM-A2DC-01A-11R-A18L-13 | TCGA-GM-A2DC |
| TCGA-BRCA | Primary Tumor | TCGA-GM-A2DD-01A-11R-A17X-13 | TCGA-GM-A2DD |
| TCGA-BRCA | Primary Tumor | TCGA-GM-A2DF-01A-11R-A17X-13 | TCGA-GM-A2DF |
| TCGA-BRCA | Primary Tumor | TCGA-GM-A2DH-01A-11R-A17X-13 | TCGA-GM-A2DH |
| TCGA-BRCA | Primary Tumor | TCGA-GM-A2DI-01A-31R-A18L-13 | TCGA-GM-A2DI |
| TCGA-BRCA | Primary Tumor | TCGA-GM-A2DK-01A-21R-A17X-13 | TCGA-GM-A2DK |
| TCGA-BRCA | Primary Tumor | TCGA-GM-A2DL-01A-11R-A18L-13 | TCGA-GM-A2DL |
| TCGA-BRCA | Primary Tumor | TCGA-GM-A2DM-01A-11R-A17X-13 | TCGA-GM-A2DM |
| TCGA-BRCA | Primary Tumor | TCGA-GM-A2DN-01A-11R-A17X-13 | TCGA-GM-A2DN |
| TCGA-BRCA | Primary Tumor | TCGA-GM-A2DO-01A-11R-A18L-13 | TCGA-GM-A2DO |
| TCGA-BRCA | Primary Tumor | TCGA-GM-A3NW-01A-21R-A22I-13 | TCGA-GM-A3NW |
| TCGA-BRCA | Primary Tumor | TCGA-GM-A3NY-01A-11R-A21U-13 | TCGA-GM-A3NY |
| TCGA-BRCA | Primary Tumor | TCGA-GM-A3XG-01A-31R-A24J-13 | TCGA-GM-A3XG |
| TCGA-BRCA | Primary Tumor | TCGA-GM-A3XL-01A-11R-A22V-13 | TCGA-GM-A3XL |
| TCGA-BRCA | Primary Tumor | TCGA-GM-A3XN-01A-12R-A22V-13 | TCGA-GM-A3XN |
| TCGA-BRCA | Primary Tumor | TCGA-GM-A4E0-01A-12R-A25Z-13 | TCGA-GM-A4E0 |
| TCGA-BRCA | Primary Tumor | TCGA-GM-A5PV-01A-11R-A28I-13 | TCGA-GM-A5PV |
| TCGA-BRCA | Primary Tumor | TCGA-GM-A5PX-01A-12R-A28I-13 | TCGA-GM-A5PX |
| TCGA-BRCA | Primary Tumor | TCGA-HN-A2NL-01A-11R-A18L-13 | TCGA-HN-A2NL |
| TCGA-BRCA | Primary Tumor | TCGA-HN-A2OB-01A-21R-A27U-13 | TCGA-HN-A2OB |
| TCGA-BRCA | Primary Tumor | TCGA-JL-A3YW-01A-12R-A23A-13 | TCGA-JL-A3YW |
| TCGA-BRCA | Primary Tumor | TCGA-JL-A3YX-01A-11R-A22V-13 | TCGA-JL-A3YX |
| TCGA-BRCA | Primary Tumor | TCGA-LD-A66U-01A-11R-A31S-13 | TCGA-LD-A66U |
| TCGA-BRCA | Primary Tumor | TCGA-LD-A74U-01A-13R-A33A-13 | TCGA-LD-A74U |
| TCGA-BRCA | Primary Tumor | TCGA-LD-A7W5-01A-22R-A358-13 | TCGA-LD-A7W5 |
| TCGA-BRCA | Primary Tumor | TCGA-LD-A7W6-01A-81R-A358-13 | TCGA-LD-A7W6 |
| TCGA-BRCA | Primary Tumor | TCGA-LD-A9QF-01A-32R-A41G-13 | TCGA-LD-A9QF |
| TCGA-BRCA | Primary Tumor | TCGA-LL-A440-01A-11R-A24J-13 | TCGA-LL-A440 |
| TCGA-BRCA | Primary Tumor | TCGA-LL-A441-01A-11R-A24J-13 | TCGA-LL-A441 |
| TCGA-BRCA | Primary Tumor | TCGA-LL-A442-01A-11R-A24J-13 | TCGA-LL-A442 |
| TCGA-BRCA | Primary Tumor | TCGA-LL-A50Y-01A-11R-A25Z-13 | TCGA-LL-A50Y |
| TCGA-BRCA | Primary Tumor | TCGA-LL-A5YL-01A-12R-A29V-13 | TCGA-LL-A5YL |
| TCGA-BRCA | Primary Tumor | TCGA-LL-A5YM-01A-11R-A28I-13 | TCGA-LL-A5YM |
| TCGA-BRCA | Primary Tumor | TCGA-LL-A5YN-01A-11R-A28I-13 | TCGA-LL-A5YN |
| TCGA-BRCA | Primary Tumor | TCGA-LL-A5YO-01A-21R-A28I-13 | TCGA-LL-A5YO |
| TCGA-BRCA | Primary Tumor | TCGA-LL-A5YP-01A-21R-A28I-13 | TCGA-LL-A5YP |
| TCGA-BRCA | Primary Tumor | TCGA-LL-A6FP-01A-11R-A31S-13 | TCGA-LL-A6FP |
| TCGA-BRCA | Primary Tumor | TCGA-LL-A6FQ-01A-11R-A31S-13 | TCGA-LL-A6FQ |
| TCGA-BRCA | Primary Tumor | TCGA-LL-A6FR-01A-12R-A31S-13 | TCGA-LL-A6FR |
| TCGA-BRCA | Primary Tumor | TCGA-LL-A73Y-01A-11R-A33A-13 | TCGA-LL-A73Y |
| TCGA-BRCA | Primary Tumor | TCGA-LL-A73Z-01A-11R-A32K-13 | TCGA-LL-A73Z |
| TCGA-BRCA | Primary Tumor | TCGA-LL-A740-01A-21R-A32K-13 | TCGA-LL-A740 |
| TCGA-BRCA | Primary Tumor | TCGA-LL-A7SZ-01A-32R-A358-13 | TCGA-LL-A7SZ |
| TCGA-BRCA | Primary Tumor | TCGA-LL-A7T0-01A-31R-A358-13 | TCGA-LL-A7T0 |
| TCGA-BRCA | Primary Tumor | TCGA-LL-A8F5-01A-11R-A36A-13 | TCGA-LL-A8F5 |

| | | | |
|---|---|---|---|
| TCGA-BRCA | Primary Tumor | TCGA-LL-A9Q3-01A-11R-A41G-13 | TCGA-LL-A9Q3 |
| TCGA-BRCA | Primary Tumor | TCGA-LQ-A4E4-01A-11R-A25Z-13 | TCGA-LQ-A4E4 |
| TCGA-BRCA | Primary Tumor | TCGA-MS-A51U-01A-31R-A25Z-13 | TCGA-MS-A51U |
| TCGA-BRCA | Primary Tumor | TCGA-OK-A5Q2-01A-11R-A27U-13 | TCGA-OK-A5Q2 |
| TCGA-BRCA | Primary Tumor | TCGA-OL-A5D6-01A-21R-A27U-13 | TCGA-OL-A5D6 |
| TCGA-BRCA | Primary Tumor | TCGA-OL-A5D7-01A-11R-A27U-13 | TCGA-OL-A5D7 |
| TCGA-BRCA | Primary Tumor | TCGA-OL-A5D8-01A-11R-A27U-13 | TCGA-OL-A5D8 |
| TCGA-BRCA | Primary Tumor | TCGA-OL-A5DA-01A-11R-A27U-13 | TCGA-OL-A5DA |
| TCGA-BRCA | Primary Tumor | TCGA-OL-A5RU-01A-11R-A28I-13 | TCGA-OL-A5RU |
| TCGA-BRCA | Primary Tumor | TCGA-OL-A5RV-01A-12R-A28I-13 | TCGA-OL-A5RV |
| TCGA-BRCA | Primary Tumor | TCGA-OL-A5RW-01A-11R-A28I-13 | TCGA-OL-A5RW |
| TCGA-BRCA | Primary Tumor | TCGA-OL-A5RX-01A-11R-A28I-13 | TCGA-OL-A5RX |
| TCGA-BRCA | Primary Tumor | TCGA-OL-A5RY-01A-21R-A28I-13 | TCGA-OL-A5RY |
| TCGA-BRCA | Primary Tumor | TCGA-OL-A5RZ-01A-11R-A28I-13 | TCGA-OL-A5RZ |
| TCGA-BRCA | Primary Tumor | TCGA-OL-A5S0-01A-11R-A28I-13 | TCGA-OL-A5S0 |
| TCGA-BRCA | Primary Tumor | TCGA-OL-A66H-01A-11R-A29V-13 | TCGA-OL-A66H |
| TCGA-BRCA | Primary Tumor | TCGA-OL-A66I-01A-21R-A29V-13 | TCGA-OL-A66I |
| TCGA-BRCA | Primary Tumor | TCGA-OL-A66J-01A-11R-A29V-13 | TCGA-OL-A66J |
| TCGA-BRCA | Primary Tumor | TCGA-OL-A66K-01A-11R-A29V-13 | TCGA-OL-A66K |
| TCGA-BRCA | Primary Tumor | TCGA-OL-A66L-01A-12R-A31S-13 | TCGA-OL-A66L |
| TCGA-BRCA | Primary Tumor | TCGA-OL-A66N-01A-12R-A31S-13 | TCGA-OL-A66N |
| TCGA-BRCA | Primary Tumor | TCGA-OL-A66O-01A-11R-A31S-13 | TCGA-OL-A66O |
| TCGA-BRCA | Primary Tumor | TCGA-OL-A66P-01A-11R-A31S-13 | TCGA-OL-A66P |
| TCGA-BRCA | Primary Tumor | TCGA-OL-A6VO-01A-12R-A33A-13 | TCGA-OL-A6VO |
| TCGA-BRCA | Primary Tumor | TCGA-OL-A6VQ-01A-12R-A41G-13 | TCGA-OL-A6VQ |
| TCGA-BRCA | Primary Tumor | TCGA-OL-A6VR-01A-32R-A33A-13 | TCGA-OL-A6VR |
| TCGA-BRCA | Primary Tumor | TCGA-OL-A97C-01A-32R-A41G-13 | TCGA-OL-A97C |
| TCGA-BRCA | Primary Tumor | TCGA-PE-A5DC-01A-12R-A27U-13 | TCGA-PE-A5DC |
| TCGA-BRCA | Primary Tumor | TCGA-PE-A5DD-01A-12R-A27U-13 | TCGA-PE-A5DD |
| TCGA-BRCA | Primary Tumor | TCGA-PE-A5DE-01A-11R-A27U-13 | TCGA-PE-A5DE |
| TCGA-BRCA | Primary Tumor | TCGA-PL-A8LV-01A-21R-A41G-13 | TCGA-PL-A8LV |
| TCGA-BRCA | Primary Tumor | TCGA-PL-A8LX-01A-11R-A41G-13 | TCGA-PL-A8LX |
| TCGA-BRCA | Primary Tumor | TCGA-PL-A8LY-01A-11R-A41G-13 | TCGA-PL-A8LY |
| TCGA-BRCA | Primary Tumor | TCGA-PL-A8LZ-01A-31R-A36A-13 | TCGA-PL-A8LZ |
| TCGA-BRCA | Primary Tumor | TCGA-S3-A6ZF-01A-32R-A32K-13 | TCGA-S3-A6ZF |
| TCGA-BRCA | Primary Tumor | TCGA-S3-A6ZG-01A-22R-A32K-13 | TCGA-S3-A6ZG |
| TCGA-BRCA | Primary Tumor | TCGA-S3-A6ZH-01A-22R-A32K-13 | TCGA-S3-A6ZH |
| TCGA-BRCA | Primary Tumor | TCGA-S3-AA0Z-01A-11R-A41G-13 | TCGA-S3-AA0Z |
| TCGA-BRCA | Primary Tumor | TCGA-S3-AA10-01A-21R-A41G-13 | TCGA-S3-AA10 |
| TCGA-BRCA | Primary Tumor | TCGA-S3-AA11-01A-31R-A41G-13 | TCGA-S3-AA11 |
| TCGA-BRCA | Primary Tumor | TCGA-S3-AA12-01A-11R-A41G-13 | TCGA-S3-AA12 |
| TCGA-BRCA | Primary Tumor | TCGA-S3-AA14-01A-11R-A41G-13 | TCGA-S3-AA14 |
| TCGA-BRCA | Primary Tumor | TCGA-S3-AA15-01A-11R-A41G-13 | TCGA-S3-AA15 |
| TCGA-BRCA | Primary Tumor | TCGA-S3-AA17-01A-11R-A41G-13 | TCGA-S3-AA17 |
| TCGA-BRCA | Primary Tumor | TCGA-UL-AAZ6-01A-11R-A41G-13 | TCGA-UL-AAZ6 |
| TCGA-BRCA | Primary Tumor | TCGA-UU-A93S-01A-21R-A41G-13 | TCGA-UU-A93S |
| TCGA-BRCA | Primary Tumor | TCGA-V7-A7HQ-01A-11R-A33A-13 | TCGA-V7-A7HQ |
| TCGA-BRCA | Primary Tumor | TCGA-W8-A86G-01A-21R-A36A-13 | TCGA-W8-A86G |
| TCGA-BRCA | Primary Tumor | TCGA-WT-AB41-01A-11R-A41G-13 | TCGA-WT-AB41 |
| TCGA-BRCA | Primary Tumor | TCGA-WT-AB44-01A-11R-A41G-13 | TCGA-WT-AB44 |
| TCGA-BRCA | Primary Tumor | TCGA-XX-A899-01A-11R-A36A-13 | TCGA-XX-A899 |
| TCGA-BRCA | Primary Tumor | TCGA-XX-A89A-01A-11R-A36A-13 | TCGA-XX-A89A |
| TCGA-BRCA | Primary Tumor | TCGA-Z7-A8R5-01A-42R-A41G-13 | TCGA-Z7-A8R5 |
| TCGA-BRCA | Primary Tumor | TCGA-Z7-A8R6-01A-11R-A41G-13 | TCGA-Z7-A8R6 |
| TCGA-UCEC | Primary Tumor | TCGA-2E-A9G8-01A-11R-A404-13 | TCGA-2E-A9G8 |
| TCGA-UCEC | Primary Tumor | TCGA-4E-A92E-01A-11R-A37P-13 | TCGA-4E-A92E |
| TCGA-UCEC | Primary Tumor | TCGA-5B-A90C-01A-11R-A37P-13 | TCGA-5B-A90C |
| TCGA-UCEC | Primary Tumor | TCGA-5S-A9Q8-01A-11R-A404-13 | TCGA-5S-A9Q8 |
| TCGA-UCEC | Primary Tumor | TCGA-A5-A0G1-01A-11R-A119-13 | TCGA-A5-A0G1 |

| | | | |
|---|---|---|---|
| TCGA-UCEC | Primary Tumor | TCGA-A5-A0G2-01A-11R-A041-13 | TCGA-A5-A0G2 |
| TCGA-UCEC | Primary Tumor | TCGA-A5-A0G3-01A-11R-A041-13 | TCGA-A5-A0G3 |
| TCGA-UCEC | Primary Tumor | TCGA-A5-A0G5-01A-11R-A041-13 | TCGA-A5-A0G5 |
| TCGA-UCEC | Primary Tumor | TCGA-A5-A0G9-01A-11R-A041-13 | TCGA-A5-A0G9 |
| TCGA-UCEC | Primary Tumor | TCGA-A5-A0GA-01A-11R-A041-13 | TCGA-A5-A0GA |
| TCGA-UCEC | Primary Tumor | TCGA-A5-A0GB-01A-11R-A041-13 | TCGA-A5-A0GB |
| TCGA-UCEC | Primary Tumor | TCGA-A5-A0GD-01A-11R-A041-13 | TCGA-A5-A0GD |
| TCGA-UCEC | Primary Tumor | TCGA-A5-A0GE-01A-11R-A041-13 | TCGA-A5-A0GE |
| TCGA-UCEC | Primary Tumor | TCGA-A5-A0GG-01A-11R-A119-13 | TCGA-A5-A0GG |
| TCGA-UCEC | Primary Tumor | TCGA-A5-A0GH-01A-21R-A041-13 | TCGA-A5-A0GH |
| TCGA-UCEC | Primary Tumor | TCGA-A5-A0GI-01A-11R-A041-13 | TCGA-A5-A0GI |
| TCGA-UCEC | Primary Tumor | TCGA-A5-A0GJ-01A-11R-A041-13 | TCGA-A5-A0GJ |
| TCGA-UCEC | Primary Tumor | TCGA-A5-A0GM-01A-11R-A041-13 | TCGA-A5-A0GM |
| TCGA-UCEC | Primary Tumor | TCGA-A5-A0GN-01A-11R-A041-13 | TCGA-A5-A0GN |
| TCGA-UCEC | Primary Tumor | TCGA-A5-A0GP-01A-11R-A041-13 | TCGA-A5-A0GP |
| TCGA-UCEC | Primary Tumor | TCGA-A5-A0GQ-01A-11R-A119-13 | TCGA-A5-A0GQ |
| TCGA-UCEC | Primary Tumor | TCGA-A5-A0GR-01A-11R-A119-13 | TCGA-A5-A0GR |
| TCGA-UCEC | Primary Tumor | TCGA-A5-A0GU-01A-11R-A041-13 | TCGA-A5-A0GU |
| TCGA-UCEC | Primary Tumor | TCGA-A5-A0GV-01A-31R-A041-13 | TCGA-A5-A0GV |
| TCGA-UCEC | Primary Tumor | TCGA-A5-A0GW-01A-11R-A041-13 | TCGA-A5-A0GW |
| TCGA-UCEC | Primary Tumor | TCGA-A5-A0GX-01A-11R-A041-13 | TCGA-A5-A0GX |
| TCGA-UCEC | Primary Tumor | TCGA-A5-A0R6-01A-11R-A103-13 | TCGA-A5-A0R6 |
| TCGA-UCEC | Primary Tumor | TCGA-A5-A0R7-01A-31R-A103-13 | TCGA-A5-A0R7 |
| TCGA-UCEC | Primary Tumor | TCGA-A5-A0R8-01A-11R-A103-13 | TCGA-A5-A0R8 |
| TCGA-UCEC | Primary Tumor | TCGA-A5-A0R9-01A-11R-A103-13 | TCGA-A5-A0R9 |
| TCGA-UCEC | Primary Tumor | TCGA-A5-A0RA-01A-21R-A103-13 | TCGA-A5-A0RA |
| TCGA-UCEC | Primary Tumor | TCGA-A5-A0VO-01A-21R-A108-13 | TCGA-A5-A0VO |
| TCGA-UCEC | Primary Tumor | TCGA-A5-A0VP-01A-21R-A103-13 | TCGA-A5-A0VP |
| TCGA-UCEC | Primary Tumor | TCGA-A5-A0VQ-01A-11R-A103-13 | TCGA-A5-A0VQ |
| TCGA-UCEC | Primary Tumor | TCGA-A5-A1OF-01A-11R-A14C-13 | TCGA-A5-A1OF |
| TCGA-UCEC | Primary Tumor | TCGA-A5-A1OG-01A-11R-A14C-13 | TCGA-A5-A1OG |
| TCGA-UCEC | Primary Tumor | TCGA-A5-A1OH-01A-21R-A22I-13 | TCGA-A5-A1OH |
| TCGA-UCEC | Primary Tumor | TCGA-A5-A1OJ-01A-11R-A14C-13 | TCGA-A5-A1OJ |
| TCGA-UCEC | Primary Tumor | TCGA-A5-A1OK-01A-11R-A14L-13 | TCGA-A5-A1OK |
| TCGA-UCEC | Primary Tumor | TCGA-A5-A2K2-01A-11R-A18L-13 | TCGA-A5-A2K2 |
| TCGA-UCEC | Primary Tumor | TCGA-A5-A2K3-01A-11R-A17X-13 | TCGA-A5-A2K3 |
| TCGA-UCEC | Primary Tumor | TCGA-A5-A2K4-01A-11R-A18L-13 | TCGA-A5-A2K4 |
| TCGA-UCEC | Primary Tumor | TCGA-A5-A2K5-01A-11R-A17X-13 | TCGA-A5-A2K5 |
| TCGA-UCEC | Primary Tumor | TCGA-A5-A2K7-01A-11R-A17X-13 | TCGA-A5-A2K7 |
| TCGA-UCEC | Primary Tumor | TCGA-A5-A3LO-01A-11R-A22I-13 | TCGA-A5-A3LO |
| TCGA-UCEC | Primary Tumor | TCGA-A5-A3LP-01A-11R-A22I-13 | TCGA-A5-A3LP |
| TCGA-UCEC | Primary Tumor | TCGA-A5-A7WJ-01A-12R-A34O-13 | TCGA-A5-A7WJ |
| TCGA-UCEC | Primary Tumor | TCGA-A5-A7WK-01A-11R-A34O-13 | TCGA-A5-A7WK |
| TCGA-UCEC | Primary Tumor | TCGA-A5-AB3J-01A-11R-A404-13 | TCGA-A5-AB3J |
| TCGA-UCEC | Primary Tumor | TCGA-AJ-A23M-01A-11R-A156-13 | TCGA-AJ-A23M |
| TCGA-UCEC | Primary Tumor | TCGA-AJ-A23N-01A-11R-A22I-13 | TCGA-AJ-A23N |
| TCGA-UCEC | Primary Tumor | TCGA-AJ-A23O-01A-11R-A156-13 | TCGA-AJ-A23O |
| TCGA-UCEC | Primary Tumor | TCGA-AJ-A2QK-01A-11R-A18L-13 | TCGA-AJ-A2QK |
| TCGA-UCEC | Primary Tumor | TCGA-AJ-A2QL-01A-11R-A18L-13 | TCGA-AJ-A2QL |
| TCGA-UCEC | Primary Tumor | TCGA-AJ-A2QM-01A-11R-A18L-13 | TCGA-AJ-A2QM |
| TCGA-UCEC | Primary Tumor | TCGA-AJ-A2QN-01A-11R-A18L-13 | TCGA-AJ-A2QN |
| TCGA-UCEC | Primary Tumor | TCGA-AJ-A2QO-01A-11R-A18L-13 | TCGA-AJ-A2QO |
| TCGA-UCEC | Primary Tumor | TCGA-AJ-A3BD-01A-11R-A19V-13 | TCGA-AJ-A3BD |
| TCGA-UCEC | Primary Tumor | TCGA-AJ-A3BF-01A-11R-A214-13 | TCGA-AJ-A3BF |
| TCGA-UCEC | Primary Tumor | TCGA-AJ-A3BG-01A-11R-A19V-13 | TCGA-AJ-A3BG |
| TCGA-UCEC | Primary Tumor | TCGA-AJ-A3BH-01A-11R-A19V-13 | TCGA-AJ-A3BH |
| TCGA-UCEC | Primary Tumor | TCGA-AJ-A3BI-01A-11R-A214-13 | TCGA-AJ-A3BI |
| TCGA-UCEC | Primary Tumor | TCGA-AJ-A3BK-01A-11R-A19V-13 | TCGA-AJ-A3BK |
| TCGA-UCEC | Primary Tumor | TCGA-AJ-A3EJ-01A-11R-A19V-13 | TCGA-AJ-A3EJ |

| | | | |
|---|---|---|---|
| TCGA-UCEC | Primary Tumor | TCGA-AJ-A3EK-01A-11R-A19V-13 | TCGA-AJ-A3EK |
| TCGA-UCEC | Primary Tumor | TCGA-AJ-A3EL-01A-11R-A214-13 | TCGA-AJ-A3EL |
| TCGA-UCEC | Primary Tumor | TCGA-AJ-A3EM-01A-11R-A214-13 | TCGA-AJ-A3EM |
| TCGA-UCEC | Primary Tumor | TCGA-AJ-A3I9-01A-11R-A22I-13 | TCGA-AJ-A3I9 |
| TCGA-UCEC | Primary Tumor | TCGA-AJ-A3IA-01A-11R-A214-13 | TCGA-AJ-A3IA |
| TCGA-UCEC | Primary Tumor | TCGA-AJ-A3NC-01A-11R-A22I-13 | TCGA-AJ-A3NC |
| TCGA-UCEC | Primary Tumor | TCGA-AJ-A3NE-01A-11R-A22I-13 | TCGA-AJ-A3NE |
| TCGA-UCEC | Primary Tumor | TCGA-AJ-A3NF-01A-11R-A22I-13 | TCGA-AJ-A3NF |
| TCGA-UCEC | Primary Tumor | TCGA-AJ-A3NG-01A-11R-A22I-13 | TCGA-AJ-A3NG |
| TCGA-UCEC | Primary Tumor | TCGA-AJ-A3NH-01A-11R-A22I-13 | TCGA-AJ-A3NH |
| TCGA-UCEC | Primary Tumor | TCGA-AJ-A3OJ-01A-11R-A22I-13 | TCGA-AJ-A3OJ |
| TCGA-UCEC | Primary Tumor | TCGA-AJ-A3OK-01A-12R-A22I-13 | TCGA-AJ-A3OK |
| TCGA-UCEC | Primary Tumor | TCGA-AJ-A3OL-01A-11R-A22I-13 | TCGA-AJ-A3OL |
| TCGA-UCEC | Primary Tumor | TCGA-AJ-A3QS-01A-11R-A22I-13 | TCGA-AJ-A3QS |
| TCGA-UCEC | Primary Tumor | TCGA-AJ-A3TW-01A-11R-A22I-13 | TCGA-AJ-A3TW |
| TCGA-UCEC | Primary Tumor | TCGA-AJ-A5DV-01A-11R-A27R-13 | TCGA-AJ-A5DV |
| TCGA-UCEC | Primary Tumor | TCGA-AJ-A5DW-01A-11R-A27R-13 | TCGA-AJ-A5DW |
| TCGA-UCEC | Primary Tumor | TCGA-AJ-A6NU-01A-11R-A34O-13 | TCGA-AJ-A6NU |
| TCGA-UCEC | Primary Tumor | TCGA-AJ-A8CT-01A-11R-A37P-13 | TCGA-AJ-A8CT |
| TCGA-UCEC | Primary Tumor | TCGA-AJ-A8CV-01A-11R-A37P-13 | TCGA-AJ-A8CV |
| TCGA-UCEC | Primary Tumor | TCGA-AJ-A8CW-01A-11R-A37P-13 | TCGA-AJ-A8CW |
| TCGA-UCEC | Primary Tumor | TCGA-AP-A051-01A-21R-A00W-13 | TCGA-AP-A051 |
| TCGA-UCEC | Primary Tumor | TCGA-AP-A053-01A-21R-A00W-13 | TCGA-AP-A053 |
| TCGA-UCEC | Primary Tumor | TCGA-AP-A054-01A-11R-A041-13 | TCGA-AP-A054 |
| TCGA-UCEC | Primary Tumor | TCGA-AP-A059-01A-21R-A119-13 | TCGA-AP-A059 |
| TCGA-UCEC | Primary Tumor | TCGA-AP-A05O-01A-12R-A119-13 | TCGA-AP-A05O |
| TCGA-UCEC | Primary Tumor | TCGA-AP-A05P-01A-11R-A041-13 | TCGA-AP-A05P |
| TCGA-UCEC | Primary Tumor | TCGA-AP-A0L8-01A-11R-A041-13 | TCGA-AP-A0L8 |
| TCGA-UCEC | Primary Tumor | TCGA-AP-A0L9-01A-11R-A041-13 | TCGA-AP-A0L9 |
| TCGA-UCEC | Primary Tumor | TCGA-AP-A0LD-01A-11R-A041-13 | TCGA-AP-A0LD |
| TCGA-UCEC | Primary Tumor | TCGA-AP-A0LE-01A-11R-A103-13 | TCGA-AP-A0LE |
| TCGA-UCEC | Primary Tumor | TCGA-AP-A0LF-01A-11R-A119-13 | TCGA-AP-A0LF |
| TCGA-UCEC | Primary Tumor | TCGA-AP-A0LG-01A-11R-A041-13 | TCGA-AP-A0LG |
| TCGA-UCEC | Primary Tumor | TCGA-AP-A0LH-01A-11R-A041-13 | TCGA-AP-A0LH |
| TCGA-UCEC | Primary Tumor | TCGA-AP-A0LI-01A-11R-A041-13 | TCGA-AP-A0LI |
| TCGA-UCEC | Primary Tumor | TCGA-AP-A0LJ-01A-11R-A041-13 | TCGA-AP-A0LJ |
| TCGA-UCEC | Primary Tumor | TCGA-AP-A0LL-01A-12R-A103-13 | TCGA-AP-A0LL |
| TCGA-UCEC | Primary Tumor | TCGA-AP-A0LM-01A-11R-A119-13 | TCGA-AP-A0LM |
| TCGA-UCEC | Primary Tumor | TCGA-AP-A0LN-01A-11R-A041-13 | TCGA-AP-A0LN |
| TCGA-UCEC | Primary Tumor | TCGA-AP-A0LO-01A-11R-A041-13 | TCGA-AP-A0LO |
| TCGA-UCEC | Primary Tumor | TCGA-AP-A0LP-01A-12R-A103-13 | TCGA-AP-A0LP |
| TCGA-UCEC | Primary Tumor | TCGA-AP-A0LQ-01A-12R-A103-13 | TCGA-AP-A0LQ |
| TCGA-UCEC | Primary Tumor | TCGA-AP-A0LS-01A-11R-A14C-13 | TCGA-AP-A0LS |
| TCGA-UCEC | Primary Tumor | TCGA-AP-A0LT-01A-11R-A041-13 | TCGA-AP-A0LT |
| TCGA-UCEC | Primary Tumor | TCGA-AP-A0LV-01A-11R-A041-13 | TCGA-AP-A0LV |
| TCGA-UCEC | Primary Tumor | TCGA-AP-A1DH-01A-31R-A136-13 | TCGA-AP-A1DH |
| TCGA-UCEC | Primary Tumor | TCGA-AP-A1DK-01A-11R-A136-13 | TCGA-AP-A1DK |
| TCGA-UCEC | Primary Tumor | TCGA-AP-A1DM-01A-21R-A136-13 | TCGA-AP-A1DM |
| TCGA-UCEC | Primary Tumor | TCGA-AP-A1DO-01A-11R-A136-13 | TCGA-AP-A1DO |
| TCGA-UCEC | Primary Tumor | TCGA-AP-A1DP-01A-11R-A136-13 | TCGA-AP-A1DP |
| TCGA-UCEC | Primary Tumor | TCGA-AP-A1DQ-01A-11R-A136-13 | TCGA-AP-A1DQ |
| TCGA-UCEC | Primary Tumor | TCGA-AP-A1DR-01A-11R-A136-13 | TCGA-AP-A1DR |
| TCGA-UCEC | Primary Tumor | TCGA-AP-A1DV-01A-21R-A136-13 | TCGA-AP-A1DV |
| TCGA-UCEC | Primary Tumor | TCGA-AP-A1E0-01A-11R-A136-13 | TCGA-AP-A1E0 |
| TCGA-UCEC | Primary Tumor | TCGA-AP-A1E1-01A-11R-A136-13 | TCGA-AP-A1E1 |
| TCGA-UCEC | Primary Tumor | TCGA-AP-A1E3-01A-11R-A136-13 | TCGA-AP-A1E3 |
| TCGA-UCEC | Primary Tumor | TCGA-AP-A1E4-01A-12R-A136-13 | TCGA-AP-A1E4 |
| TCGA-UCEC | Primary Tumor | TCGA-AP-A3K1-01A-11R-A214-13 | TCGA-AP-A3K1 |
| TCGA-UCEC | Primary Tumor | TCGA-AP-A5FX-01A-11R-A27R-13 | TCGA-AP-A5FX |

| | | | |
|---|---|---|---|
| TCGA-UCEC | Primary Tumor | TCGA-AW-A1PO-01A-12R-A156-13 | TCGA-AW-A1PO |
| TCGA-UCEC | Primary Tumor | TCGA-AX-A05S-01A-11R-A00W-13 | TCGA-AX-A05S |
| TCGA-UCEC | Primary Tumor | TCGA-AX-A05T-01A-11R-A00W-13 | TCGA-AX-A05T |
| TCGA-UCEC | Primary Tumor | TCGA-AX-A05U-01A-11R-A00W-13 | TCGA-AX-A05U |
| TCGA-UCEC | Primary Tumor | TCGA-AX-A05W-01A-21R-A00W-13 | TCGA-AX-A05W |
| TCGA-UCEC | Primary Tumor | TCGA-AX-A05Y-01A-11R-A00W-13 | TCGA-AX-A05Y |
| TCGA-UCEC | Primary Tumor | TCGA-AX-A05Z-01A-11R-A00W-13 | TCGA-AX-A05Z |
| TCGA-UCEC | Primary Tumor | TCGA-AX-A060-01A-11R-A00W-13 | TCGA-AX-A060 |
| TCGA-UCEC | Primary Tumor | TCGA-AX-A062-01A-11R-A00W-13 | TCGA-AX-A062 |
| TCGA-UCEC | Primary Tumor | TCGA-AX-A063-01A-11R-A00W-13 | TCGA-AX-A063 |
| TCGA-UCEC | Primary Tumor | TCGA-AX-A064-01A-11R-A00W-13 | TCGA-AX-A064 |
| TCGA-UCEC | Primary Tumor | TCGA-AX-A06B-01A-11R-A00W-13 | TCGA-AX-A06B |
| TCGA-UCEC | Primary Tumor | TCGA-AX-A06D-01A-11R-A119-13 | TCGA-AX-A06D |
| TCGA-UCEC | Primary Tumor | TCGA-AX-A06F-01A-11R-A00W-13 | TCGA-AX-A06F |
| TCGA-UCEC | Primary Tumor | TCGA-AX-A06H-01A-11R-A119-13 | TCGA-AX-A06H |
| TCGA-UCEC | Primary Tumor | TCGA-AX-A06J-01A-11R-A00W-13 | TCGA-AX-A06J |
| TCGA-UCEC | Primary Tumor | TCGA-AX-A06L-01A-11R-A119-13 | TCGA-AX-A06L |
| TCGA-UCEC | Primary Tumor | TCGA-AX-A0IS-01A-12R-A10I-13 | TCGA-AX-A0IS |
| TCGA-UCEC | Primary Tumor | TCGA-AX-A0IU-01A-11R-A103-13 | TCGA-AX-A0IU |
| TCGA-UCEC | Primary Tumor | TCGA-AX-A0IW-01A-11R-A041-13 | TCGA-AX-A0IW |
| TCGA-UCEC | Primary Tumor | TCGA-AX-A0IZ-01A-11R-A119-13 | TCGA-AX-A0IZ |
| TCGA-UCEC | Primary Tumor | TCGA-AX-A0J0-01A-11R-A108-13 | TCGA-AX-A0J0 |
| TCGA-UCEC | Primary Tumor | TCGA-AX-A0J1-01A-11R-A041-13 | TCGA-AX-A0J1 |
| TCGA-UCEC | Primary Tumor | TCGA-AX-A1C4-01A-11R-A136-13 | TCGA-AX-A1C4 |
| TCGA-UCEC | Primary Tumor | TCGA-AX-A1C5-01A-11R-A136-13 | TCGA-AX-A1C5 |
| TCGA-UCEC | Primary Tumor | TCGA-AX-A1C7-01A-11R-A136-13 | TCGA-AX-A1C7 |
| TCGA-UCEC | Primary Tumor | TCGA-AX-A1C8-01A-11R-A136-13 | TCGA-AX-A1C8 |
| TCGA-UCEC | Primary Tumor | TCGA-AX-A1C9-01A-11R-A136-13 | TCGA-AX-A1C9 |
| TCGA-UCEC | Primary Tumor | TCGA-AX-A1CA-01A-12R-A136-13 | TCGA-AX-A1CA |
| TCGA-UCEC | Primary Tumor | TCGA-AX-A1CC-01A-11R-A136-13 | TCGA-AX-A1CC |
| TCGA-UCEC | Primary Tumor | TCGA-AX-A1CE-01A-11R-A136-13 | TCGA-AX-A1CE |
| TCGA-UCEC | Primary Tumor | TCGA-AX-A1CF-01A-11R-A136-13 | TCGA-AX-A1CF |
| TCGA-UCEC | Primary Tumor | TCGA-AX-A1CI-01A-11R-A136-13 | TCGA-AX-A1CI |
| TCGA-UCEC | Primary Tumor | TCGA-AX-A1CJ-01A-11R-A136-13 | TCGA-AX-A1CJ |
| TCGA-UCEC | Primary Tumor | TCGA-AX-A1CK-01A-11R-A136-13 | TCGA-AX-A1CK |
| TCGA-UCEC | Primary Tumor | TCGA-AX-A1CN-01A-11R-A136-13 | TCGA-AX-A1CN |
| TCGA-UCEC | Primary Tumor | TCGA-AX-A1CP-01A-11R-A136-13 | TCGA-AX-A1CP |
| TCGA-UCEC | Primary Tumor | TCGA-AX-A1CR-01A-12R-A136-13 | TCGA-AX-A1CR |
| TCGA-UCEC | Primary Tumor | TCGA-AX-A2H2-01A-11R-A17X-13 | TCGA-AX-A2H2 |
| TCGA-UCEC | Primary Tumor | TCGA-AX-A2H4-01A-21R-A18L-13 | TCGA-AX-A2H4 |
| TCGA-UCEC | Primary Tumor | TCGA-AX-A2H5-01A-11R-A17A-13 | TCGA-AX-A2H5 |
| TCGA-UCEC | Primary Tumor | TCGA-AX-A2H7-01A-12R-A18L-13 | TCGA-AX-A2H7 |
| TCGA-UCEC | Primary Tumor | TCGA-AX-A2H8-01A-11R-A17A-13 | TCGA-AX-A2H8 |
| TCGA-UCEC | Primary Tumor | TCGA-AX-A2HA-01A-12R-A18L-13 | TCGA-AX-A2HA |
| TCGA-UCEC | Primary Tumor | TCGA-AX-A2HC-01A-11R-A17A-13 | TCGA-AX-A2HC |
| TCGA-UCEC | Primary Tumor | TCGA-AX-A2HD-01A-21R-A17A-13 | TCGA-AX-A2HD |
| TCGA-UCEC | Primary Tumor | TCGA-AX-A2HF-01A-11R-A17A-13 | TCGA-AX-A2HF |
| TCGA-UCEC | Primary Tumor | TCGA-AX-A2HG-01A-11R-A17A-13 | TCGA-AX-A2HG |
| TCGA-UCEC | Primary Tumor | TCGA-AX-A2HH-01A-11R-A17A-13 | TCGA-AX-A2HH |
| TCGA-UCEC | Primary Tumor | TCGA-AX-A2HJ-01A-11R-A17A-13 | TCGA-AX-A2HJ |
| TCGA-UCEC | Primary Tumor | TCGA-AX-A2HK-01A-11R-A17A-13 | TCGA-AX-A2HK |
| TCGA-UCEC | Primary Tumor | TCGA-AX-A2IN-01A-12R-A17X-13 | TCGA-AX-A2IN |
| TCGA-UCEC | Primary Tumor | TCGA-AX-A2IO-01A-11R-A17X-13 | TCGA-AX-A2IO |
| TCGA-UCEC | Primary Tumor | TCGA-AX-A3FS-01A-11R-A22I-13 | TCGA-AX-A3FS |
| TCGA-UCEC | Primary Tumor | TCGA-AX-A3FT-01A-11R-A22I-13 | TCGA-AX-A3FT |
| TCGA-UCEC | Primary Tumor | TCGA-AX-A3FV-01A-11R-A22I-13 | TCGA-AX-A3FV |
| TCGA-UCEC | Primary Tumor | TCGA-AX-A3FW-01A-11R-A22I-13 | TCGA-AX-A3FW |
| TCGA-UCEC | Primary Tumor | TCGA-AX-A3FX-01A-11R-A22I-13 | TCGA-AX-A3FX |
| TCGA-UCEC | Primary Tumor | TCGA-AX-A3FZ-01A-11R-A22I-13 | TCGA-AX-A3FZ |

| | | | |
|---|---|---|---|
| TCGA-UCEC | Primary Tumor | TCGA-AX-A3G1-01A-11R-A22I-13 | TCGA-AX-A3G1 |
| TCGA-UCEC | Primary Tumor | TCGA-AX-A3G3-01A-11R-A214-13 | TCGA-AX-A3G3 |
| TCGA-UCEC | Primary Tumor | TCGA-AX-A3G4-01A-11R-A214-13 | TCGA-AX-A3G4 |
| TCGA-UCEC | Primary Tumor | TCGA-AX-A3G6-01A-11R-A214-13 | TCGA-AX-A3G6 |
| TCGA-UCEC | Primary Tumor | TCGA-AX-A3G7-01A-12R-A214-13 | TCGA-AX-A3G7 |
| TCGA-UCEC | Primary Tumor | TCGA-AX-A3G8-01A-11R-A22I-13 | TCGA-AX-A3G8 |
| TCGA-UCEC | Primary Tumor | TCGA-AX-A3G9-01A-11R-A22I-13 | TCGA-AX-A3G9 |
| TCGA-UCEC | Primary Tumor | TCGA-AX-A3GB-01A-11R-A22I-13 | TCGA-AX-A3GB |
| TCGA-UCEC | Primary Tumor | TCGA-AX-A3GI-01A-11R-A214-13 | TCGA-AX-A3GI |
| TCGA-UCEC | Primary Tumor | TCGA-B5-A0JN-01A-11R-A103-13 | TCGA-B5-A0JN |
| TCGA-UCEC | Primary Tumor | TCGA-B5-A0JR-01A-13R-A041-13 | TCGA-B5-A0JR |
| TCGA-UCEC | Primary Tumor | TCGA-B5-A0JS-01A-11R-A103-13 | TCGA-B5-A0JS |
| TCGA-UCEC | Primary Tumor | TCGA-B5-A0JT-01A-21R-A119-13 | TCGA-B5-A0JT |
| TCGA-UCEC | Primary Tumor | TCGA-B5-A0JU-01B-11R-A14C-13 | TCGA-B5-A0JU |
| TCGA-UCEC | Primary Tumor | TCGA-B5-A0JV-01A-11R-A103-13 | TCGA-B5-A0JV |
| TCGA-UCEC | Primary Tumor | TCGA-B5-A0JX-01A-21R-A14L-13 | TCGA-B5-A0JX |
| TCGA-UCEC | Primary Tumor | TCGA-B5-A0JY-01A-11R-A103-13 | TCGA-B5-A0JY |
| TCGA-UCEC | Primary Tumor | TCGA-B5-A0JZ-01A-11R-A041-13 | TCGA-B5-A0JZ |
| TCGA-UCEC | Primary Tumor | TCGA-B5-A0K0-01A-11R-A041-13 | TCGA-B5-A0K0 |
| TCGA-UCEC | Primary Tumor | TCGA-B5-A0K1-01A-11R-A041-13 | TCGA-B5-A0K1 |
| TCGA-UCEC | Primary Tumor | TCGA-B5-A0K2-01A-12R-A103-13 | TCGA-B5-A0K2 |
| TCGA-UCEC | Primary Tumor | TCGA-B5-A0K3-01A-11R-A041-13 | TCGA-B5-A0K3 |
| TCGA-UCEC | Primary Tumor | TCGA-B5-A0K4-01A-11R-A041-13 | TCGA-B5-A0K4 |
| TCGA-UCEC | Primary Tumor | TCGA-B5-A0K6-01A-11R-A041-13 | TCGA-B5-A0K6 |
| TCGA-UCEC | Primary Tumor | TCGA-B5-A0K7-01A-11R-A103-13 | TCGA-B5-A0K7 |
| TCGA-UCEC | Primary Tumor | TCGA-B5-A0K8-01A-11R-A14L-13 | TCGA-B5-A0K8 |
| TCGA-UCEC | Primary Tumor | TCGA-B5-A0K9-01A-21R-A103-13 | TCGA-B5-A0K9 |
| TCGA-UCEC | Primary Tumor | TCGA-B5-A0KB-01B-11R-A14C-13 | TCGA-B5-A0KB |
| TCGA-UCEC | Primary Tumor | TCGA-B5-A11E-01A-11R-A10I-13 | TCGA-B5-A11E |
| TCGA-UCEC | Primary Tumor | TCGA-B5-A11F-01A-11R-A10I-13 | TCGA-B5-A11F |
| TCGA-UCEC | Primary Tumor | TCGA-B5-A11G-01A-13R-A119-13 | TCGA-B5-A11G |
| TCGA-UCEC | Primary Tumor | TCGA-B5-A11H-01A-11R-A119-13 | TCGA-B5-A11H |
| TCGA-UCEC | Primary Tumor | TCGA-B5-A11I-01A-11R-A10I-13 | TCGA-B5-A11I |
| TCGA-UCEC | Primary Tumor | TCGA-B5-A11J-01A-11R-A119-13 | TCGA-B5-A11J |
| TCGA-UCEC | Primary Tumor | TCGA-B5-A11L-01B-21R-A13R-13 | TCGA-B5-A11L |
| TCGA-UCEC | Primary Tumor | TCGA-B5-A11M-01A-11R-A119-13 | TCGA-B5-A11M |
| TCGA-UCEC | Primary Tumor | TCGA-B5-A11N-01A-11R-A119-13 | TCGA-B5-A11N |
| TCGA-UCEC | Primary Tumor | TCGA-B5-A11O-01A-11R-A119-13 | TCGA-B5-A11O |
| TCGA-UCEC | Primary Tumor | TCGA-B5-A11P-01B-11R-A14C-13 | TCGA-B5-A11P |
| TCGA-UCEC | Primary Tumor | TCGA-B5-A11Q-01A-11R-A119-13 | TCGA-B5-A11Q |
| TCGA-UCEC | Primary Tumor | TCGA-B5-A11R-01A-11R-A119-13 | TCGA-B5-A11R |
| TCGA-UCEC | Primary Tumor | TCGA-B5-A11S-01A-11R-A119-13 | TCGA-B5-A11S |
| TCGA-UCEC | Primary Tumor | TCGA-B5-A11U-01A-11R-A119-13 | TCGA-B5-A11U |
| TCGA-UCEC | Primary Tumor | TCGA-B5-A11V-01A-11R-A10I-13 | TCGA-B5-A11V |
| TCGA-UCEC | Primary Tumor | TCGA-B5-A11W-01A-12R-A119-13 | TCGA-B5-A11W |
| TCGA-UCEC | Primary Tumor | TCGA-B5-A11X-01A-11R-A10I-13 | TCGA-B5-A11X |
| TCGA-UCEC | Primary Tumor | TCGA-B5-A11Y-01A-21R-A10I-13 | TCGA-B5-A11Y |
| TCGA-UCEC | Primary Tumor | TCGA-B5-A11Z-01A-11R-A10I-13 | TCGA-B5-A11Z |
| TCGA-UCEC | Primary Tumor | TCGA-B5-A121-01A-31R-A119-13 | TCGA-B5-A121 |
| TCGA-UCEC | Primary Tumor | TCGA-B5-A1MR-01A-31R-A14C-13 | TCGA-B5-A1MR |
| TCGA-UCEC | Primary Tumor | TCGA-B5-A1MS-01B-11R-A22I-13 | TCGA-B5-A1MS |
| TCGA-UCEC | Primary Tumor | TCGA-B5-A1MU-01A-11R-A13R-13 | TCGA-B5-A1MU |
| TCGA-UCEC | Primary Tumor | TCGA-B5-A1MV-01A-31R-A14C-13 | TCGA-B5-A1MV |
| TCGA-UCEC | Primary Tumor | TCGA-B5-A1MW-01A-11R-A17A-13 | TCGA-B5-A1MW |
| TCGA-UCEC | Primary Tumor | TCGA-B5-A1MX-01A-11R-A143-13 | TCGA-B5-A1MX |
| TCGA-UCEC | Primary Tumor | TCGA-B5-A1MY-01A-11R-A143-13 | TCGA-B5-A1MY |
| TCGA-UCEC | Primary Tumor | TCGA-B5-A1MZ-01A-11R-A143-13 | TCGA-B5-A1MZ |
| TCGA-UCEC | Primary Tumor | TCGA-B5-A1N2-01A-21R-A143-13 | TCGA-B5-A1N2 |
| TCGA-UCEC | Primary Tumor | TCGA-B5-A3F9-01A-21R-A22I-13 | TCGA-B5-A3F9 |

| | | | |
|---|---|---|---|
| TCGA-UCEC | Primary Tumor | TCGA-B5-A3FA-01A-11R-A19V-13 | TCGA-B5-A3FA |
| TCGA-UCEC | Primary Tumor | TCGA-B5-A3FB-01A-11R-A19V-13 | TCGA-B5-A3FB |
| TCGA-UCEC | Primary Tumor | TCGA-B5-A3FC-01A-11R-A22I-13 | TCGA-B5-A3FC |
| TCGA-UCEC | Primary Tumor | TCGA-B5-A3FD-01A-11R-A19V-13 | TCGA-B5-A3FD |
| TCGA-UCEC | Primary Tumor | TCGA-B5-A3FH-01A-11R-A19V-13 | TCGA-B5-A3FH |
| TCGA-UCEC | Primary Tumor | TCGA-B5-A3S1-01A-11R-A22I-13 | TCGA-B5-A3S1 |
| TCGA-UCEC | Primary Tumor | TCGA-B5-A5OC-01A-21R-A27R-13 | TCGA-B5-A5OC |
| TCGA-UCEC | Primary Tumor | TCGA-B5-A5OD-01A-11R-A31S-13 | TCGA-B5-A5OD |
| TCGA-UCEC | Primary Tumor | TCGA-B5-A5OE-01A-11R-A31S-13 | TCGA-B5-A5OE |
| TCGA-UCEC | Primary Tumor | TCGA-BG-A0LW-01A-11R-A041-13 | TCGA-BG-A0LW |
| TCGA-UCEC | Primary Tumor | TCGA-BG-A0LX-01A-11R-A041-13 | TCGA-BG-A0LX |
| TCGA-UCEC | Primary Tumor | TCGA-BG-A0M0-01A-11R-A103-13 | TCGA-BG-A0M0 |
| TCGA-UCEC | Primary Tumor | TCGA-BG-A0M2-01A-11R-A103-13 | TCGA-BG-A0M2 |
| TCGA-UCEC | Primary Tumor | TCGA-BG-A0M3-01A-11R-A103-13 | TCGA-BG-A0M3 |
| TCGA-UCEC | Primary Tumor | TCGA-BG-A0M4-01A-11R-A103-13 | TCGA-BG-A0M4 |
| TCGA-UCEC | Primary Tumor | TCGA-BG-A0M6-01A-31R-A103-13 | TCGA-BG-A0M6 |
| TCGA-UCEC | Primary Tumor | TCGA-BG-A0M7-01A-11R-A041-13 | TCGA-BG-A0M7 |
| TCGA-UCEC | Primary Tumor | TCGA-BG-A0M8-01A-12R-A103-13 | TCGA-BG-A0M8 |
| TCGA-UCEC | Primary Tumor | TCGA-BG-A0M9-01A-21R-A103-13 | TCGA-BG-A0M9 |
| TCGA-UCEC | Primary Tumor | TCGA-BG-A0MA-01A-11R-A17A-13 | TCGA-BG-A0MA |
| TCGA-UCEC | Primary Tumor | TCGA-BG-A0MC-01A-21R-A041-13 | TCGA-BG-A0MC |
| TCGA-UCEC | Primary Tumor | TCGA-BG-A0MG-01A-21R-A103-13 | TCGA-BG-A0MG |
| TCGA-UCEC | Primary Tumor | TCGA-BG-A0MH-01A-11R-A119-13 | TCGA-BG-A0MH |
| TCGA-UCEC | Primary Tumor | TCGA-BG-A0MI-01A-11R-A041-13 | TCGA-BG-A0MI |
| TCGA-UCEC | Primary Tumor | TCGA-BG-A0MK-01A-51R-A19V-13 | TCGA-BG-A0MK |
| TCGA-UCEC | Primary Tumor | TCGA-BG-A0MO-01A-11R-A041-13 | TCGA-BG-A0MO |
| TCGA-UCEC | Primary Tumor | TCGA-BG-A0MQ-01A-11R-A103-13 | TCGA-BG-A0MQ |
| TCGA-UCEC | Primary Tumor | TCGA-BG-A0MS-01A-11R-A103-13 | TCGA-BG-A0MS |
| TCGA-UCEC | Primary Tumor | TCGA-BG-A0MT-01A-11R-A103-13 | TCGA-BG-A0MT |
| TCGA-UCEC | Primary Tumor | TCGA-BG-A0MU-01A-11R-A103-13 | TCGA-BG-A0MU |
| TCGA-UCEC | Primary Tumor | TCGA-BG-A0RY-01A-11R-A103-13 | TCGA-BG-A0RY |
| TCGA-UCEC | Primary Tumor | TCGA-BG-A0VT-01A-11R-A10I-13 | TCGA-BG-A0VT |
| TCGA-UCEC | Primary Tumor | TCGA-BG-A0VV-01A-21R-A119-13 | TCGA-BG-A0VV |
| TCGA-UCEC | Primary Tumor | TCGA-BG-A0VW-01A-11R-A119-13 | TCGA-BG-A0VW |
| TCGA-UCEC | Primary Tumor | TCGA-BG-A0VX-01A-11R-A119-13 | TCGA-BG-A0VX |
| TCGA-UCEC | Primary Tumor | TCGA-BG-A0VZ-01A-11R-A108-13 | TCGA-BG-A0VZ |
| TCGA-UCEC | Primary Tumor | TCGA-BG-A0W1-01A-12R-A108-13 | TCGA-BG-A0W1 |
| TCGA-UCEC | Primary Tumor | TCGA-BG-A0W2-01A-11R-A108-13 | TCGA-BG-A0W2 |
| TCGA-UCEC | Primary Tumor | TCGA-BG-A0YU-01A-21R-A10I-13 | TCGA-BG-A0YU |
| TCGA-UCEC | Primary Tumor | TCGA-BG-A0YV-01A-11R-A10I-13 | TCGA-BG-A0YV |
| TCGA-UCEC | Primary Tumor | TCGA-BG-A186-01A-11R-A12H-13 | TCGA-BG-A186 |
| TCGA-UCEC | Primary Tumor | TCGA-BG-A187-01A-11R-A12H-13 | TCGA-BG-A187 |
| TCGA-UCEC | Primary Tumor | TCGA-BG-A18A-01A-21R-A12H-13 | TCGA-BG-A18A |
| TCGA-UCEC | Primary Tumor | TCGA-BG-A18B-01A-11R-A12H-13 | TCGA-BG-A18B |
| TCGA-UCEC | Primary Tumor | TCGA-BG-A18C-01A-11R-A12H-13 | TCGA-BG-A18C |
| TCGA-UCEC | Primary Tumor | TCGA-BG-A221-01A-21R-A156-13 | TCGA-BG-A221 |
| TCGA-UCEC | Primary Tumor | TCGA-BG-A222-01A-11R-A156-13 | TCGA-BG-A222 |
| TCGA-UCEC | Primary Tumor | TCGA-BG-A2AD-01A-21R-A16E-13 | TCGA-BG-A2AD |
| TCGA-UCEC | Primary Tumor | TCGA-BG-A2AE-01A-11R-A16E-13 | TCGA-BG-A2AE |
| TCGA-UCEC | Primary Tumor | TCGA-BG-A2L7-01A-11R-A18L-13 | TCGA-BG-A2L7 |
| TCGA-UCEC | Primary Tumor | TCGA-BG-A3EW-01A-11R-A22I-13 | TCGA-BG-A3EW |
| TCGA-UCEC | Primary Tumor | TCGA-BG-A3PP-01A-11R-A22I-13 | TCGA-BG-A3PP |
| TCGA-UCEC | Primary Tumor | TCGA-BK-A0C9-01A-11R-A00W-13 | TCGA-BK-A0C9 |
| TCGA-UCEC | Primary Tumor | TCGA-BK-A0CA-01A-21R-A119-13 | TCGA-BK-A0CA |
| TCGA-UCEC | Primary Tumor | TCGA-BK-A0CA-01A-21R-A27D-13 | TCGA-BK-A0CA |
| TCGA-UCEC | Primary Tumor | TCGA-BK-A0CA-01B-02R-A27D-13 | TCGA-BK-A0CA |
| TCGA-UCEC | Primary Tumor | TCGA-BK-A0CC-01A-21R-A00W-13 | TCGA-BK-A0CC |
| TCGA-UCEC | Primary Tumor | TCGA-BK-A0CC-01A-21R-A27D-13 | TCGA-BK-A0CC |
| TCGA-UCEC | Primary Tumor | TCGA-BK-A0CC-01B-04R-A27D-13 | TCGA-BK-A0CC |

| | | | |
|---|---|---|---|
| TCGA-UCEC | Primary Tumor | TCGA-BK-A139-01A-11R-A119-13 | TCGA-BK-A139 |
| TCGA-UCEC | Primary Tumor | TCGA-BK-A139-01A-11R-A27D-13 | TCGA-BK-A139 |
| TCGA-UCEC | Primary Tumor | TCGA-BK-A13B-01A-51R-A22I-13 | TCGA-BK-A13B |
| TCGA-UCEC | Primary Tumor | TCGA-BK-A13C-01A-11R-A119-13 | TCGA-BK-A13C |
| TCGA-UCEC | Primary Tumor | TCGA-BK-A26L-01A-11R-A16E-13 | TCGA-BK-A26L |
| TCGA-UCEC | Primary Tumor | TCGA-BK-A26L-01A-11R-A27D-13 | TCGA-BK-A26L |
| TCGA-UCEC | Primary Tumor | TCGA-BK-A26L-01C-04R-A27D-13 | TCGA-BK-A26L |
| TCGA-UCEC | Primary Tumor | TCGA-BK-A4ZD-01A-11R-A27R-13 | TCGA-BK-A4ZD |
| TCGA-UCEC | Primary Tumor | TCGA-BK-A56F-01A-32R-A27R-13 | TCGA-BK-A56F |
| TCGA-UCEC | Primary Tumor | TCGA-BK-A6W3-01A-12R-A34O-13 | TCGA-BK-A6W3 |
| TCGA-UCEC | Primary Tumor | TCGA-BK-A6W4-01A-12R-A34O-13 | TCGA-BK-A6W4 |
| TCGA-UCEC | Primary Tumor | TCGA-BS-A0T9-01A-11R-A12H-13 | TCGA-BS-A0T9 |
| TCGA-UCEC | Primary Tumor | TCGA-BS-A0TA-01A-11R-A103-13 | TCGA-BS-A0TA |
| TCGA-UCEC | Primary Tumor | TCGA-BS-A0TC-01A-11R-A103-13 | TCGA-BS-A0TC |
| TCGA-UCEC | Primary Tumor | TCGA-BS-A0TD-01A-11R-A103-13 | TCGA-BS-A0TD |
| TCGA-UCEC | Primary Tumor | TCGA-BS-A0TE-01A-11R-A103-13 | TCGA-BS-A0TE |
| TCGA-UCEC | Primary Tumor | TCGA-BS-A0TG-01A-32R-A103-13 | TCGA-BS-A0TG |
| TCGA-UCEC | Primary Tumor | TCGA-BS-A0TI-01A-11R-A103-13 | TCGA-BS-A0TI |
| TCGA-UCEC | Primary Tumor | TCGA-BS-A0TJ-01A-11R-A103-13 | TCGA-BS-A0TJ |
| TCGA-UCEC | Primary Tumor | TCGA-BS-A0U5-01A-11R-A108-13 | TCGA-BS-A0U5 |
| TCGA-UCEC | Primary Tumor | TCGA-BS-A0U7-01A-21R-A103-13 | TCGA-BS-A0U7 |
| TCGA-UCEC | Primary Tumor | TCGA-BS-A0U8-01A-11R-A103-13 | TCGA-BS-A0U8 |
| TCGA-UCEC | Primary Tumor | TCGA-BS-A0U9-01B-21R-A10I-13 | TCGA-BS-A0U9 |
| TCGA-UCEC | Primary Tumor | TCGA-BS-A0UA-01A-11R-A119-13 | TCGA-BS-A0UA |
| TCGA-UCEC | Primary Tumor | TCGA-BS-A0UF-01A-11R-A103-13 | TCGA-BS-A0UF |
| TCGA-UCEC | Primary Tumor | TCGA-BS-A0UJ-01A-12R-A103-13 | TCGA-BS-A0UJ |
| TCGA-UCEC | Primary Tumor | TCGA-BS-A0UL-01A-11R-A108-13 | TCGA-BS-A0UL |
| TCGA-UCEC | Primary Tumor | TCGA-BS-A0UM-01A-11R-A103-13 | TCGA-BS-A0UM |
| TCGA-UCEC | Primary Tumor | TCGA-BS-A0UT-01A-11R-A103-13 | TCGA-BS-A0UT |
| TCGA-UCEC | Primary Tumor | TCGA-BS-A0UV-01A-11R-A103-13 | TCGA-BS-A0UV |
| TCGA-UCEC | Primary Tumor | TCGA-BS-A0V4-01A-11R-A14C-13 | TCGA-BS-A0V4 |
| TCGA-UCEC | Primary Tumor | TCGA-BS-A0V6-01A-11R-A119-13 | TCGA-BS-A0V6 |
| TCGA-UCEC | Primary Tumor | TCGA-BS-A0V7-01A-21R-A119-13 | TCGA-BS-A0V7 |
| TCGA-UCEC | Primary Tumor | TCGA-BS-A0V8-01A-11R-A119-13 | TCGA-BS-A0V8 |
| TCGA-UCEC | Primary Tumor | TCGA-BS-A0VI-01A-11R-A14C-13 | TCGA-BS-A0VI |
| TCGA-UCEC | Primary Tumor | TCGA-BS-A0WQ-01A-21R-A108-13 | TCGA-BS-A0WQ |
| TCGA-UCEC | Primary Tumor | TCGA-D1-A0ZN-01A-11R-A119-13 | TCGA-D1-A0ZN |
| TCGA-UCEC | Primary Tumor | TCGA-D1-A0ZO-01A-11R-A119-13 | TCGA-D1-A0ZO |
| TCGA-UCEC | Primary Tumor | TCGA-D1-A0ZP-01A-21R-A10I-13 | TCGA-D1-A0ZP |
| TCGA-UCEC | Primary Tumor | TCGA-D1-A0ZQ-01A-11R-A119-13 | TCGA-D1-A0ZQ |
| TCGA-UCEC | Primary Tumor | TCGA-D1-A0ZR-01A-21R-A10I-13 | TCGA-D1-A0ZR |
| TCGA-UCEC | Primary Tumor | TCGA-D1-A0ZS-01A-11R-A119-13 | TCGA-D1-A0ZS |
| TCGA-UCEC | Primary Tumor | TCGA-D1-A0ZU-01A-11R-A10I-13 | TCGA-D1-A0ZU |
| TCGA-UCEC | Primary Tumor | TCGA-D1-A0ZV-01A-11R-A10I-13 | TCGA-D1-A0ZV |
| TCGA-UCEC | Primary Tumor | TCGA-D1-A0ZZ-01A-11R-A10I-13 | TCGA-D1-A0ZZ |
| TCGA-UCEC | Primary Tumor | TCGA-D1-A101-01A-12R-A10I-13 | TCGA-D1-A101 |
| TCGA-UCEC | Primary Tumor | TCGA-D1-A102-01A-11R-A10I-13 | TCGA-D1-A102 |
| TCGA-UCEC | Primary Tumor | TCGA-D1-A103-01A-11R-A10I-13 | TCGA-D1-A103 |
| TCGA-UCEC | Primary Tumor | TCGA-D1-A15V-01A-11R-A119-13 | TCGA-D1-A15V |
| TCGA-UCEC | Primary Tumor | TCGA-D1-A15W-01A-11R-A119-13 | TCGA-D1-A15W |
| TCGA-UCEC | Primary Tumor | TCGA-D1-A15X-01A-11R-A119-13 | TCGA-D1-A15X |
| TCGA-UCEC | Primary Tumor | TCGA-D1-A15Z-01A-11R-A119-13 | TCGA-D1-A15Z |
| TCGA-UCEC | Primary Tumor | TCGA-D1-A160-01A-11R-A119-13 | TCGA-D1-A160 |
| TCGA-UCEC | Primary Tumor | TCGA-D1-A161-01A-11R-A119-13 | TCGA-D1-A161 |
| TCGA-UCEC | Primary Tumor | TCGA-D1-A162-01A-11R-A119-13 | TCGA-D1-A162 |
| TCGA-UCEC | Primary Tumor | TCGA-D1-A163-01A-11R-A12H-13 | TCGA-D1-A163 |
| TCGA-UCEC | Primary Tumor | TCGA-D1-A165-01A-11R-A12H-13 | TCGA-D1-A165 |
| TCGA-UCEC | Primary Tumor | TCGA-D1-A167-01A-11R-A12H-13 | TCGA-D1-A167 |
| TCGA-UCEC | Primary Tumor | TCGA-D1-A168-01A-31R-A12H-13 | TCGA-D1-A168 |

| | | | |
|---|---|---|---|
| TCGA-UCEC | Primary Tumor | TCGA-D1-A169-01A-11R-A12H-13 | TCGA-D1-A169 |
| TCGA-UCEC | Primary Tumor | TCGA-D1-A16B-01A-11R-A12H-13 | TCGA-D1-A16B |
| TCGA-UCEC | Primary Tumor | TCGA-D1-A16D-01A-11R-A12H-13 | TCGA-D1-A16D |
| TCGA-UCEC | Primary Tumor | TCGA-D1-A16E-01A-22R-A12H-13 | TCGA-D1-A16E |
| TCGA-UCEC | Primary Tumor | TCGA-D1-A16F-01A-11R-A12H-13 | TCGA-D1-A16F |
| TCGA-UCEC | Primary Tumor | TCGA-D1-A16G-01A-31R-A12H-13 | TCGA-D1-A16G |
| TCGA-UCEC | Primary Tumor | TCGA-D1-A16I-01A-11R-A12H-13 | TCGA-D1-A16I |
| TCGA-UCEC | Primary Tumor | TCGA-D1-A16J-01A-11R-A12H-13 | TCGA-D1-A16J |
| TCGA-UCEC | Primary Tumor | TCGA-D1-A16N-01A-11R-A12H-13 | TCGA-D1-A16N |
| TCGA-UCEC | Primary Tumor | TCGA-D1-A16O-01A-11R-A12H-13 | TCGA-D1-A16O |
| TCGA-UCEC | Primary Tumor | TCGA-D1-A16Q-01A-12R-A12H-13 | TCGA-D1-A16Q |
| TCGA-UCEC | Primary Tumor | TCGA-D1-A16R-01A-11R-A12H-13 | TCGA-D1-A16R |
| TCGA-UCEC | Primary Tumor | TCGA-D1-A16S-01A-11R-A12H-13 | TCGA-D1-A16S |
| TCGA-UCEC | Primary Tumor | TCGA-D1-A16V-01A-11R-A12H-13 | TCGA-D1-A16V |
| TCGA-UCEC | Primary Tumor | TCGA-D1-A16X-01A-11R-A12H-13 | TCGA-D1-A16X |
| TCGA-UCEC | Primary Tumor | TCGA-D1-A16Y-01A-31R-A12H-13 | TCGA-D1-A16Y |
| TCGA-UCEC | Primary Tumor | TCGA-D1-A174-01A-11R-A12H-13 | TCGA-D1-A174 |
| TCGA-UCEC | Primary Tumor | TCGA-D1-A175-01A-11R-A12H-13 | TCGA-D1-A175 |
| TCGA-UCEC | Primary Tumor | TCGA-D1-A176-01A-11R-A12H-13 | TCGA-D1-A176 |
| TCGA-UCEC | Primary Tumor | TCGA-D1-A177-01A-21R-A12H-13 | TCGA-D1-A177 |
| TCGA-UCEC | Primary Tumor | TCGA-D1-A179-01A-11R-A12H-13 | TCGA-D1-A179 |
| TCGA-UCEC | Primary Tumor | TCGA-D1-A17A-01A-11R-A12H-13 | TCGA-D1-A17A |
| TCGA-UCEC | Primary Tumor | TCGA-D1-A17B-01A-22R-A12H-13 | TCGA-D1-A17B |
| TCGA-UCEC | Primary Tumor | TCGA-D1-A17C-01A-11R-A12H-13 | TCGA-D1-A17C |
| TCGA-UCEC | Primary Tumor | TCGA-D1-A17D-01A-12R-A12H-13 | TCGA-D1-A17D |
| TCGA-UCEC | Primary Tumor | TCGA-D1-A17F-01A-11R-A12H-13 | TCGA-D1-A17F |
| TCGA-UCEC | Primary Tumor | TCGA-D1-A17H-01A-11R-A12H-13 | TCGA-D1-A17H |
| TCGA-UCEC | Primary Tumor | TCGA-D1-A17K-01A-11R-A12H-13 | TCGA-D1-A17K |
| TCGA-UCEC | Primary Tumor | TCGA-D1-A17L-01A-11R-A12H-13 | TCGA-D1-A17L |
| TCGA-UCEC | Primary Tumor | TCGA-D1-A17M-01A-21R-A12H-13 | TCGA-D1-A17M |
| TCGA-UCEC | Primary Tumor | TCGA-D1-A17N-01A-11R-A12H-13 | TCGA-D1-A17N |
| TCGA-UCEC | Primary Tumor | TCGA-D1-A17Q-01A-11R-A12H-13 | TCGA-D1-A17Q |
| TCGA-UCEC | Primary Tumor | TCGA-D1-A17R-01A-11R-A12H-13 | TCGA-D1-A17R |
| TCGA-UCEC | Primary Tumor | TCGA-D1-A17S-01A-11R-A12H-13 | TCGA-D1-A17S |
| TCGA-UCEC | Primary Tumor | TCGA-D1-A17T-01A-11R-A12H-13 | TCGA-D1-A17T |
| TCGA-UCEC | Primary Tumor | TCGA-D1-A17U-01A-21R-A12H-13 | TCGA-D1-A17U |
| TCGA-UCEC | Primary Tumor | TCGA-D1-A1NS-01A-11R-A14C-13 | TCGA-D1-A1NS |
| TCGA-UCEC | Primary Tumor | TCGA-D1-A1NU-01A-11R-A14C-13 | TCGA-D1-A1NU |
| TCGA-UCEC | Primary Tumor | TCGA-D1-A1NW-01A-11R-A14L-13 | TCGA-D1-A1NW |
| TCGA-UCEC | Primary Tumor | TCGA-D1-A1NX-01A-11R-A16E-13 | TCGA-D1-A1NX |
| TCGA-UCEC | Primary Tumor | TCGA-D1-A1NY-01A-11R-A16E-13 | TCGA-D1-A1NY |
| TCGA-UCEC | Primary Tumor | TCGA-D1-A1NZ-01A-21R-A14C-13 | TCGA-D1-A1NZ |
| TCGA-UCEC | Primary Tumor | TCGA-D1-A1O0-01A-11R-A17A-13 | TCGA-D1-A1O0 |
| TCGA-UCEC | Primary Tumor | TCGA-D1-A1O5-01A-11R-A14C-13 | TCGA-D1-A1O5 |
| TCGA-UCEC | Primary Tumor | TCGA-D1-A1O7-01A-11R-A14C-13 | TCGA-D1-A1O7 |
| TCGA-UCEC | Primary Tumor | TCGA-D1-A1O8-01A-11R-A14C-13 | TCGA-D1-A1O8 |
| TCGA-UCEC | Primary Tumor | TCGA-D1-A2G0-01A-11R-A17A-13 | TCGA-D1-A2G0 |
| TCGA-UCEC | Primary Tumor | TCGA-D1-A2G5-01A-11R-A17A-13 | TCGA-D1-A2G5 |
| TCGA-UCEC | Primary Tumor | TCGA-D1-A2G6-01A-11R-A17A-13 | TCGA-D1-A2G6 |
| TCGA-UCEC | Primary Tumor | TCGA-D1-A2G7-01A-21R-A17X-13 | TCGA-D1-A2G7 |
| TCGA-UCEC | Primary Tumor | TCGA-D1-A3DA-01A-12R-A214-13 | TCGA-D1-A3DA |
| TCGA-UCEC | Primary Tumor | TCGA-D1-A3DG-01A-11R-A19V-13 | TCGA-D1-A3DG |
| TCGA-UCEC | Primary Tumor | TCGA-D1-A3DH-01A-11R-A19V-13 | TCGA-D1-A3DH |
| TCGA-UCEC | Primary Tumor | TCGA-D1-A3JP-01A-31R-A22I-13 | TCGA-D1-A3JP |
| TCGA-UCEC | Primary Tumor | TCGA-D1-A3JQ-01A-11R-A22I-13 | TCGA-D1-A3JQ |
| TCGA-UCEC | Primary Tumor | TCGA-DF-A2KN-01A-11R-A17X-13 | TCGA-DF-A2KN |
| TCGA-UCEC | Primary Tumor | TCGA-DF-A2KR-01A-11R-A17X-13 | TCGA-DF-A2KR |
| TCGA-UCEC | Primary Tumor | TCGA-DF-A2KS-01A-11R-A18L-13 | TCGA-DF-A2KS |
| TCGA-UCEC | Primary Tumor | TCGA-DF-A2KU-01A-11R-A17X-13 | TCGA-DF-A2KU |

| | | | |
|---|---|---|---|
| TCGA-UCEC | Primary Tumor | TCGA-DF-A2KV-01A-11R-A17X-13 | TCGA-DF-A2KV |
| TCGA-UCEC | Primary Tumor | TCGA-DF-A2KY-01A-21R-A214-13 | TCGA-DF-A2KY |
| TCGA-UCEC | Primary Tumor | TCGA-DF-A2KZ-01A-11R-A214-13 | TCGA-DF-A2KZ |
| TCGA-UCEC | Primary Tumor | TCGA-DF-A2L0-01A-11R-A17X-13 | TCGA-DF-A2L0 |
| TCGA-UCEC | Primary Tumor | TCGA-DI-A0WH-01A-12R-A12H-13 | TCGA-DI-A0WH |
| TCGA-UCEC | Primary Tumor | TCGA-DI-A1BU-01A-11R-A136-13 | TCGA-DI-A1BU |
| TCGA-UCEC | Primary Tumor | TCGA-DI-A1BY-01A-21R-A136-13 | TCGA-DI-A1BY |
| TCGA-UCEC | Primary Tumor | TCGA-DI-A1C3-01A-41R-A136-13 | TCGA-DI-A1C3 |
| TCGA-UCEC | Primary Tumor | TCGA-DI-A1NN-01A-11R-A16E-13 | TCGA-DI-A1NN |
| TCGA-UCEC | Primary Tumor | TCGA-DI-A1NO-01A-31R-A156-13 | TCGA-DI-A1NO |
| TCGA-UCEC | Primary Tumor | TCGA-DI-A2QT-01A-12R-A19V-13 | TCGA-DI-A2QT |
| TCGA-UCEC | Primary Tumor | TCGA-DI-A2QU-01A-11R-A18L-13 | TCGA-DI-A2QU |
| TCGA-UCEC | Primary Tumor | TCGA-DI-A2QY-01A-12R-A19V-13 | TCGA-DI-A2QY |
| TCGA-UCEC | Primary Tumor | TCGA-E6-A1LX-01A-11R-A14C-13 | TCGA-E6-A1LX |
| TCGA-UCEC | Primary Tumor | TCGA-E6-A1LZ-01A-11R-A143-13 | TCGA-E6-A1LZ |
| TCGA-UCEC | Primary Tumor | TCGA-E6-A1M0-01A-11R-A143-13 | TCGA-E6-A1M0 |
| TCGA-UCEC | Primary Tumor | TCGA-E6-A2P8-01A-11R-A19V-13 | TCGA-E6-A2P8 |
| TCGA-UCEC | Primary Tumor | TCGA-E6-A2P9-01A-11R-A19V-13 | TCGA-E6-A2P9 |
| TCGA-UCEC | Primary Tumor | TCGA-E6-A8L9-01A-21R-A37P-13 | TCGA-E6-A8L9 |
| TCGA-UCEC | Primary Tumor | TCGA-EC-A1NJ-01A-31R-A14C-13 | TCGA-EC-A1NJ |
| TCGA-UCEC | Primary Tumor | TCGA-EC-A1QX-01A-31R-A16E-13 | TCGA-EC-A1QX |
| TCGA-UCEC | Primary Tumor | TCGA-EC-A24G-01A-11R-A16E-13 | TCGA-EC-A24G |
| TCGA-UCEC | Primary Tumor | TCGA-EO-A1Y5-01A-11R-A156-13 | TCGA-EO-A1Y5 |
| TCGA-UCEC | Primary Tumor | TCGA-EO-A1Y7-01A-11R-A156-13 | TCGA-EO-A1Y7 |
| TCGA-UCEC | Primary Tumor | TCGA-EO-A1Y8-01A-11R-A156-13 | TCGA-EO-A1Y8 |
| TCGA-UCEC | Primary Tumor | TCGA-EO-A22R-01A-11R-A18L-13 | TCGA-EO-A22R |
| TCGA-UCEC | Primary Tumor | TCGA-EO-A22S-01A-11R-A18L-13 | TCGA-EO-A22S |
| TCGA-UCEC | Primary Tumor | TCGA-EO-A22T-01A-21R-A18L-13 | TCGA-EO-A22T |
| TCGA-UCEC | Primary Tumor | TCGA-EO-A22U-01A-11R-A17X-13 | TCGA-EO-A22U |
| TCGA-UCEC | Primary Tumor | TCGA-EO-A22X-01A-11R-A17X-13 | TCGA-EO-A22X |
| TCGA-UCEC | Primary Tumor | TCGA-EO-A22Y-01A-11R-A17X-13 | TCGA-EO-A22Y |
| TCGA-UCEC | Primary Tumor | TCGA-EO-A2CG-01A-12R-A17X-13 | TCGA-EO-A2CG |
| TCGA-UCEC | Primary Tumor | TCGA-EO-A2CH-01A-11R-A17X-13 | TCGA-EO-A2CH |
| TCGA-UCEC | Primary Tumor | TCGA-EO-A3AS-01A-11R-A19V-13 | TCGA-EO-A3AS |
| TCGA-UCEC | Primary Tumor | TCGA-EO-A3AU-01A-21R-A19V-13 | TCGA-EO-A3AU |
| TCGA-UCEC | Primary Tumor | TCGA-EO-A3AV-01A-12R-A19V-13 | TCGA-EO-A3AV |
| TCGA-UCEC | Primary Tumor | TCGA-EO-A3AY-01A-12R-A19V-13 | TCGA-EO-A3AY |
| TCGA-UCEC | Primary Tumor | TCGA-EO-A3AZ-01A-12R-A19V-13 | TCGA-EO-A3AZ |
| TCGA-UCEC | Primary Tumor | TCGA-EO-A3B0-01A-12R-A19V-13 | TCGA-EO-A3B0 |
| TCGA-UCEC | Primary Tumor | TCGA-EO-A3B1-01A-12R-A19V-13 | TCGA-EO-A3B1 |
| TCGA-UCEC | Primary Tumor | TCGA-EO-A3KU-01A-11R-A22I-13 | TCGA-EO-A3KU |
| TCGA-UCEC | Primary Tumor | TCGA-EO-A3KW-01A-11R-A22I-13 | TCGA-EO-A3KW |
| TCGA-UCEC | Primary Tumor | TCGA-EO-A3KX-01A-11R-A22I-13 | TCGA-EO-A3KX |
| TCGA-UCEC | Primary Tumor | TCGA-EO-A3L0-01A-11R-A22I-13 | TCGA-EO-A3L0 |
| TCGA-UCEC | Primary Tumor | TCGA-EY-A1G7-01A-11R-A13R-13 | TCGA-EY-A1G7 |
| TCGA-UCEC | Primary Tumor | TCGA-EY-A1G8-01A-11R-A13R-13 | TCGA-EY-A1G8 |
| TCGA-UCEC | Primary Tumor | TCGA-EY-A1GC-01A-11R-A13R-13 | TCGA-EY-A1GC |
| TCGA-UCEC | Primary Tumor | TCGA-EY-A1GD-01A-11R-A13R-13 | TCGA-EY-A1GD |
| TCGA-UCEC | Primary Tumor | TCGA-EY-A1GE-01A-11R-A13R-13 | TCGA-EY-A1GE |
| TCGA-UCEC | Primary Tumor | TCGA-EY-A1GF-01A-11R-A13R-13 | TCGA-EY-A1GF |
| TCGA-UCEC | Primary Tumor | TCGA-EY-A1GH-01A-11R-A13R-13 | TCGA-EY-A1GH |
| TCGA-UCEC | Primary Tumor | TCGA-EY-A1GI-01A-11R-A13R-13 | TCGA-EY-A1GI |
| TCGA-UCEC | Primary Tumor | TCGA-EY-A1GJ-01A-12R-A143-13 | TCGA-EY-A1GJ |
| TCGA-UCEC | Primary Tumor | TCGA-EY-A1GK-01A-11R-A13R-13 | TCGA-EY-A1GK |
| TCGA-UCEC | Primary Tumor | TCGA-EY-A1GL-01A-11R-A13R-13 | TCGA-EY-A1GL |
| TCGA-UCEC | Primary Tumor | TCGA-EY-A1GM-01A-12R-A14C-13 | TCGA-EY-A1GM |
| TCGA-UCEC | Primary Tumor | TCGA-EY-A1GO-01A-11R-A14C-13 | TCGA-EY-A1GO |
| TCGA-UCEC | Primary Tumor | TCGA-EY-A1GP-01A-11R-A13R-13 | TCGA-EY-A1GP |
| TCGA-UCEC | Primary Tumor | TCGA-EY-A1GQ-01A-21R-A13R-13 | TCGA-EY-A1GQ |

| | | | |
|---|---|---|---|
| TCGA-UCEC | Primary Tumor | TCGA-EY-A1GR-01A-11R-A13R-13 | TCGA-EY-A1GR |
| TCGA-UCEC | Primary Tumor | TCGA-EY-A1GS-01A-11R-A13R-13 | TCGA-EY-A1GS |
| TCGA-UCEC | Primary Tumor | TCGA-EY-A1GT-01A-11R-A13R-13 | TCGA-EY-A1GT |
| TCGA-UCEC | Primary Tumor | TCGA-EY-A1GU-01A-11R-A13R-13 | TCGA-EY-A1GU |
| TCGA-UCEC | Primary Tumor | TCGA-EY-A1GV-01A-11R-A13R-13 | TCGA-EY-A1GV |
| TCGA-UCEC | Primary Tumor | TCGA-EY-A1GW-01A-22R-A13R-13 | TCGA-EY-A1GW |
| TCGA-UCEC | Primary Tumor | TCGA-EY-A1GX-01A-12R-A13R-13 | TCGA-EY-A1GX |
| TCGA-UCEC | Primary Tumor | TCGA-EY-A1H0-01A-11R-A13R-13 | TCGA-EY-A1H0 |
| TCGA-UCEC | Primary Tumor | TCGA-EY-A210-01A-11R-A156-13 | TCGA-EY-A210 |
| TCGA-UCEC | Primary Tumor | TCGA-EY-A212-01A-11R-A14L-13 | TCGA-EY-A212 |
| TCGA-UCEC | Primary Tumor | TCGA-EY-A214-01A-12R-A156-13 | TCGA-EY-A214 |
| TCGA-UCEC | Primary Tumor | TCGA-EY-A215-01A-11R-A14L-13 | TCGA-EY-A215 |
| TCGA-UCEC | Primary Tumor | TCGA-EY-A2OM-01A-11R-A18L-13 | TCGA-EY-A2OM |
| TCGA-UCEC | Primary Tumor | TCGA-EY-A2ON-01A-21R-A18L-13 | TCGA-EY-A2ON |
| TCGA-UCEC | Primary Tumor | TCGA-EY-A2OO-01A-11R-A19V-13 | TCGA-EY-A2OO |
| TCGA-UCEC | Primary Tumor | TCGA-EY-A2OP-01A-11R-A19V-13 | TCGA-EY-A2OP |
| TCGA-UCEC | Primary Tumor | TCGA-EY-A2OQ-01A-11R-A19V-13 | TCGA-EY-A2OQ |
| TCGA-UCEC | Primary Tumor | TCGA-EY-A3L3-01A-11R-A22I-13 | TCGA-EY-A3L3 |
| TCGA-UCEC | Primary Tumor | TCGA-EY-A3QX-01A-11R-A22I-13 | TCGA-EY-A3QX |
| TCGA-UCEC | Primary Tumor | TCGA-EY-A4KR-01A-11R-A27R-13 | TCGA-EY-A4KR |
| TCGA-UCEC | Primary Tumor | TCGA-EY-A547-01A-11R-A27R-13 | TCGA-EY-A547 |
| TCGA-UCEC | Primary Tumor | TCGA-EY-A548-01A-11R-A27R-13 | TCGA-EY-A548 |
| TCGA-UCEC | Primary Tumor | TCGA-EY-A549-01A-11R-A27R-13 | TCGA-EY-A549 |
| TCGA-UCEC | Primary Tumor | TCGA-EY-A54A-01A-11R-A27R-13 | TCGA-EY-A54A |
| TCGA-UCEC | Primary Tumor | TCGA-EY-A5W2-01A-11R-A31S-13 | TCGA-EY-A5W2 |
| TCGA-UCEC | Primary Tumor | TCGA-EY-A72D-01A-12R-A34O-13 | TCGA-EY-A72D |
| TCGA-UCEC | Primary Tumor | TCGA-FI-A2CX-01A-11R-A17A-13 | TCGA-FI-A2CX |
| TCGA-UCEC | Primary Tumor | TCGA-FI-A2CY-01A-11R-A17A-13 | TCGA-FI-A2CY |
| TCGA-UCEC | Primary Tumor | TCGA-FI-A2D0-01A-11R-A17A-13 | TCGA-FI-A2D0 |
| TCGA-UCEC | Primary Tumor | TCGA-FI-A2D2-01A-11R-A17A-13 | TCGA-FI-A2D2 |
| TCGA-UCEC | Primary Tumor | TCGA-FI-A2D4-01A-12R-A17A-13 | TCGA-FI-A2D4 |
| TCGA-UCEC | Primary Tumor | TCGA-FI-A2D5-01A-11R-A17A-13 | TCGA-FI-A2D5 |
| TCGA-UCEC | Primary Tumor | TCGA-FI-A2D6-01A-11R-A17A-13 | TCGA-FI-A2D6 |
| TCGA-UCEC | Primary Tumor | TCGA-FI-A2EU-01A-11R-A17A-13 | TCGA-FI-A2EU |
| TCGA-UCEC | Primary Tumor | TCGA-FI-A2EW-01A-11R-A17A-13 | TCGA-FI-A2EW |
| TCGA-UCEC | Primary Tumor | TCGA-FI-A2EX-01A-11R-A17A-13 | TCGA-FI-A2EX |
| TCGA-UCEC | Primary Tumor | TCGA-FI-A2EY-01A-12R-A18L-13 | TCGA-FI-A2EY |
| TCGA-UCEC | Primary Tumor | TCGA-FI-A2F4-01A-11R-A17A-13 | TCGA-FI-A2F4 |
| TCGA-UCEC | Primary Tumor | TCGA-FI-A2F8-01A-12R-A17A-13 | TCGA-FI-A2F8 |
| TCGA-UCEC | Primary Tumor | TCGA-FI-A2F9-01A-11R-A17A-13 | TCGA-FI-A2F9 |
| TCGA-UCEC | Primary Tumor | TCGA-FI-A3PV-01A-11R-A22I-13 | TCGA-FI-A3PV |
| TCGA-UCEC | Primary Tumor | TCGA-FI-A3PX-01A-11R-A22I-13 | TCGA-FI-A3PX |
| TCGA-UCEC | Primary Tumor | TCGA-H5-A2HR-01A-11R-A17X-13 | TCGA-H5-A2HR |
| TCGA-UCEC | Primary Tumor | TCGA-JU-AAVI-01A-11R-A404-13 | TCGA-JU-AAVI |
| TCGA-UCEC | Primary Tumor | TCGA-K6-A3WQ-01A-11R-A22I-13 | TCGA-K6-A3WQ |
| TCGA-UCEC | Primary Tumor | TCGA-KJ-A3U4-01A-11R-A22I-13 | TCGA-KJ-A3U4 |
| TCGA-UCEC | Primary Tumor | TCGA-KP-A3VZ-01A-11R-A22I-13 | TCGA-KP-A3VZ |
| TCGA-UCEC | Primary Tumor | TCGA-KP-A3W0-01A-21R-A22I-13 | TCGA-KP-A3W0 |
| TCGA-UCEC | Primary Tumor | TCGA-KP-A3W1-01A-11R-A22I-13 | TCGA-KP-A3W1 |
| TCGA-UCEC | Primary Tumor | TCGA-KP-A3W3-01A-11R-A22I-13 | TCGA-KP-A3W3 |
| TCGA-UCEC | Primary Tumor | TCGA-KP-A3W4-01A-11R-A22I-13 | TCGA-KP-A3W4 |
| TCGA-UCEC | Primary Tumor | TCGA-PG-A5BC-01A-12R-A27R-13 | TCGA-PG-A5BC |
| TCGA-UCEC | Primary Tumor | TCGA-PG-A6IB-01A-21R-A31S-13 | TCGA-PG-A6IB |
| TCGA-UCEC | Primary Tumor | TCGA-PG-A7D5-01A-11R-A34O-13 | TCGA-PG-A7D5 |
| TCGA-UCEC | Primary Tumor | TCGA-PG-A914-01A-11R-A37P-13 | TCGA-PG-A914 |
| TCGA-UCEC | Primary Tumor | TCGA-PG-A915-01A-11R-A37P-13 | TCGA-PG-A915 |
| TCGA-UCEC | Primary Tumor | TCGA-PG-A916-01A-11R-A37P-13 | TCGA-PG-A916 |
| TCGA-UCEC | Primary Tumor | TCGA-PG-A917-01A-31R-A37P-13 | TCGA-PG-A917 |
| TCGA-UCEC | Primary Tumor | TCGA-QF-A5YS-01A-11R-A31S-13 | TCGA-QF-A5YS |

| | | | |
|---|---|---|---|
| TCGA-UCEC | Primary Tumor | TCGA-QF-A5YT-01A-11R-A31S-13 | TCGA-QF-A5YT |
| TCGA-UCEC | Primary Tumor | TCGA-QS-A5YQ-01A-11R-A31S-13 | TCGA-QS-A5YQ |
| TCGA-UCEC | Primary Tumor | TCGA-QS-A5YR-01A-31R-A31S-13 | TCGA-QS-A5YR |
| TCGA-UCEC | Primary Tumor | TCGA-QS-A744-01A-11R-A34O-13 | TCGA-QS-A744 |
| TCGA-UCEC | Primary Tumor | TCGA-QS-A8F1-01A-21R-A37P-13 | TCGA-QS-A8F1 |
| TCGA-UCEC | Primary Tumor | TCGA-SJ-A6ZI-01A-12R-A34O-13 | TCGA-SJ-A6ZI |
| TCGA-UCEC | Primary Tumor | TCGA-SJ-A6ZJ-01A-12R-A34O-13 | TCGA-SJ-A6ZJ |
| TCGA-UCEC | Primary Tumor | TCGA-SL-A6J9-01A-11R-A31S-13 | TCGA-SL-A6J9 |
| TCGA-UCEC | Primary Tumor | TCGA-SL-A6JA-01A-11R-A31S-13 | TCGA-SL-A6JA |
| TCGA-BRCA | Solid Tissue Normal | TCGA-A7-A0CE-11A-21R-A090-13 | TCGA-A7-A0CE |
| TCGA-BRCA | Solid Tissue Normal | TCGA-A7-A0D9-11A-53R-A090-13 | TCGA-A7-A0D9 |
| TCGA-BRCA | Solid Tissue Normal | TCGA-A7-A0DB-11A-33R-A090-13 | TCGA-A7-A0DB |
| TCGA-BRCA | Solid Tissue Normal | TCGA-A7-A0DC-11A-41R-A090-13 | TCGA-A7-A0DC |
| TCGA-BRCA | Solid Tissue Normal | TCGA-A7-A13E-11A-61R-A12O-13 | TCGA-A7-A13E |
| TCGA-BRCA | Solid Tissue Normal | TCGA-A7-A13F-11A-42R-A12O-13 | TCGA-A7-A13F |
| TCGA-BRCA | Solid Tissue Normal | TCGA-A7-A13G-11A-51R-A13P-13 | TCGA-A7-A13G |
| TCGA-BRCA | Solid Tissue Normal | TCGA-AC-A23H-11A-12R-A156-13 | TCGA-AC-A23H |
| TCGA-BRCA | Solid Tissue Normal | TCGA-AC-A2FB-11A-13R-A17A-13 | TCGA-AC-A2FB |
| TCGA-BRCA | Solid Tissue Normal | TCGA-AC-A2FM-11B-32R-A19V-13 | TCGA-AC-A2FM |
| TCGA-BRCA | Solid Tissue Normal | TCGA-BH-A0AU-11A-11R-A12O-13 | TCGA-BH-A0AU |
| TCGA-BRCA | Solid Tissue Normal | TCGA-BH-A0AY-11A-23R-A090-13 | TCGA-BH-A0AY |
| TCGA-BRCA | Solid Tissue Normal | TCGA-BH-A0AZ-11A-22R-A12O-13 | TCGA-BH-A0AZ |
| TCGA-BRCA | Solid Tissue Normal | TCGA-BH-A0B3-11B-21R-A090-13 | TCGA-BH-A0B3 |
| TCGA-BRCA | Solid Tissue Normal | TCGA-BH-A0B5-11A-23R-A12O-13 | TCGA-BH-A0B5 |
| TCGA-BRCA | Solid Tissue Normal | TCGA-BH-A0B7-11A-34R-A114-13 | TCGA-BH-A0B7 |
| TCGA-BRCA | Solid Tissue Normal | TCGA-BH-A0BC-11A-22R-A090-13 | TCGA-BH-A0BC |
| TCGA-BRCA | Solid Tissue Normal | TCGA-BH-A0BJ-11A-23R-A090-13 | TCGA-BH-A0BJ |
| TCGA-BRCA | Solid Tissue Normal | TCGA-BH-A0BQ-11A-33R-A114-13 | TCGA-BH-A0BQ |
| TCGA-BRCA | Solid Tissue Normal | TCGA-BH-A0BS-11A-11R-A12O-13 | TCGA-BH-A0BS |
| TCGA-BRCA | Solid Tissue Normal | TCGA-BH-A0BT-11A-21R-A12O-13 | TCGA-BH-A0BT |
| TCGA-BRCA | Solid Tissue Normal | TCGA-BH-A0BV-11A-31R-A090-13 | TCGA-BH-A0BV |
| TCGA-BRCA | Solid Tissue Normal | TCGA-BH-A0BW-11A-12R-A114-13 | TCGA-BH-A0BW |
| TCGA-BRCA | Solid Tissue Normal | TCGA-BH-A0BZ-11A-61R-A12O-13 | TCGA-BH-A0BZ |
| TCGA-BRCA | Solid Tissue Normal | TCGA-BH-A0C0-11A-21R-A090-13 | TCGA-BH-A0C0 |
| TCGA-BRCA | Solid Tissue Normal | TCGA-BH-A0C3-11A-23R-A12O-13 | TCGA-BH-A0C3 |
| TCGA-BRCA | Solid Tissue Normal | TCGA-BH-A0DD-11A-23R-A12O-13 | TCGA-BH-A0DD |
| TCGA-BRCA | Solid Tissue Normal | TCGA-BH-A0DG-11A-43R-A12O-13 | TCGA-BH-A0DG |
| TCGA-BRCA | Solid Tissue Normal | TCGA-BH-A0DK-11A-13R-A090-13 | TCGA-BH-A0DK |
| TCGA-BRCA | Solid Tissue Normal | TCGA-BH-A0DL-11A-13R-A114-13 | TCGA-BH-A0DL |
| TCGA-BRCA | Solid Tissue Normal | TCGA-BH-A0DO-11A-22R-A12C-13 | TCGA-BH-A0DO |
| TCGA-BRCA | Solid Tissue Normal | TCGA-BH-A0DP-11A-12R-A090-13 | TCGA-BH-A0DP |
| TCGA-BRCA | Solid Tissue Normal | TCGA-BH-A0DQ-11A-12R-A090-13 | TCGA-BH-A0DQ |
| TCGA-BRCA | Solid Tissue Normal | TCGA-BH-A0DT-11A-12R-A12C-13 | TCGA-BH-A0DT |
| TCGA-BRCA | Solid Tissue Normal | TCGA-BH-A0DV-11A-22R-A12O-13 | TCGA-BH-A0DV |
| TCGA-BRCA | Solid Tissue Normal | TCGA-BH-A0DZ-11A-22R-A090-13 | TCGA-BH-A0DZ |
| TCGA-BRCA | Solid Tissue Normal | TCGA-BH-A0E1-11A-13R-A090-13 | TCGA-BH-A0E1 |
| TCGA-BRCA | Solid Tissue Normal | TCGA-BH-A0H5-11A-62R-A114-13 | TCGA-BH-A0H5 |
| TCGA-BRCA | Solid Tissue Normal | TCGA-BH-A0H7-11A-13R-A090-13 | TCGA-BH-A0H7 |
| TCGA-BRCA | Solid Tissue Normal | TCGA-BH-A0HA-11A-31R-A12O-13 | TCGA-BH-A0HA |
| TCGA-BRCA | Solid Tissue Normal | TCGA-BH-A0HK-11A-11R-A090-13 | TCGA-BH-A0HK |
| TCGA-BRCA | Solid Tissue Normal | TCGA-BH-A18J-11A-31R-A12C-13 | TCGA-BH-A18J |
| TCGA-BRCA | Solid Tissue Normal | TCGA-BH-A18K-11A-13R-A12C-13 | TCGA-BH-A18K |
| TCGA-BRCA | Solid Tissue Normal | TCGA-BH-A18L-11A-42R-A12C-13 | TCGA-BH-A18L |
| TCGA-BRCA | Solid Tissue Normal | TCGA-BH-A18M-11A-33R-A12C-13 | TCGA-BH-A18M |
| TCGA-BRCA | Solid Tissue Normal | TCGA-BH-A18N-11A-43R-A12C-13 | TCGA-BH-A18N |
| TCGA-BRCA | Solid Tissue Normal | TCGA-BH-A18P-11A-43R-A12C-13 | TCGA-BH-A18P |
| TCGA-BRCA | Solid Tissue Normal | TCGA-BH-A18Q-11A-34R-A12C-13 | TCGA-BH-A18Q |
| TCGA-BRCA | Solid Tissue Normal | TCGA-BH-A18R-11A-42R-A12C-13 | TCGA-BH-A18R |
| TCGA-BRCA | Solid Tissue Normal | TCGA-BH-A18S-11A-43R-A12C-13 | TCGA-BH-A18S |

| | | | |
|---|---|---|---|
| TCGA-BRCA | Solid Tissue Normal | TCGA-BH-A18U-11A-23R-A12C-13 | TCGA-BH-A18U |
| TCGA-BRCA | Solid Tissue Normal | TCGA-BH-A1EN-11A-23R-A13P-13 | TCGA-BH-A1EN |
| TCGA-BRCA | Solid Tissue Normal | TCGA-BH-A1EO-11A-31R-A136-13 | TCGA-BH-A1EO |
| TCGA-BRCA | Solid Tissue Normal | TCGA-BH-A1ET-11B-23R-A136-13 | TCGA-BH-A1ET |
| TCGA-BRCA | Solid Tissue Normal | TCGA-BH-A1EU-11A-23R-A136-13 | TCGA-BH-A1EU |
| TCGA-BRCA | Solid Tissue Normal | TCGA-BH-A1EV-11A-24R-A136-13 | TCGA-BH-A1EV |
| TCGA-BRCA | Solid Tissue Normal | TCGA-BH-A1EW-11B-33R-A136-13 | TCGA-BH-A1EW |
| TCGA-BRCA | Solid Tissue Normal | TCGA-BH-A1F0-11B-23R-A136-13 | TCGA-BH-A1F0 |
| TCGA-BRCA | Solid Tissue Normal | TCGA-BH-A1F2-11A-32R-A13P-13 | TCGA-BH-A1F2 |
| TCGA-BRCA | Solid Tissue Normal | TCGA-BH-A1F6-11B-94R-A13P-13 | TCGA-BH-A1F6 |
| TCGA-BRCA | Solid Tissue Normal | TCGA-BH-A1F8-11B-21R-A13P-13 | TCGA-BH-A1F8 |
| TCGA-BRCA | Solid Tissue Normal | TCGA-BH-A1FB-11A-33R-A13P-13 | TCGA-BH-A1FB |
| TCGA-BRCA | Solid Tissue Normal | TCGA-BH-A1FC-11A-32R-A13P-13 | TCGA-BH-A1FC |
| TCGA-BRCA | Solid Tissue Normal | TCGA-BH-A1FD-11B-21R-A13P-13 | TCGA-BH-A1FD |
| TCGA-BRCA | Solid Tissue Normal | TCGA-BH-A1FE-11B-14R-A13P-13 | TCGA-BH-A1FE |
| TCGA-BRCA | Solid Tissue Normal | TCGA-BH-A1FG-11B-12R-A13P-13 | TCGA-BH-A1FG |
| TCGA-BRCA | Solid Tissue Normal | TCGA-BH-A1FH-11B-42R-A13P-13 | TCGA-BH-A1FH |
| TCGA-BRCA | Solid Tissue Normal | TCGA-BH-A1FJ-11B-42R-A13P-13 | TCGA-BH-A1FJ |
| TCGA-BRCA | Solid Tissue Normal | TCGA-BH-A1FM-11B-23R-A13P-13 | TCGA-BH-A1FM |
| TCGA-BRCA | Solid Tissue Normal | TCGA-BH-A1FN-11A-34R-A13P-13 | TCGA-BH-A1FN |
| TCGA-BRCA | Solid Tissue Normal | TCGA-BH-A1FR-11B-42R-A13P-13 | TCGA-BH-A1FR |
| TCGA-BRCA | Solid Tissue Normal | TCGA-BH-A1FU-11A-23R-A14C-13 | TCGA-BH-A1FU |
| TCGA-BRCA | Solid Tissue Normal | TCGA-BH-A203-11A-42R-A168-13 | TCGA-BH-A203 |
| TCGA-BRCA | Solid Tissue Normal | TCGA-BH-A204-11A-53R-A156-13 | TCGA-BH-A204 |
| TCGA-BRCA | Solid Tissue Normal | TCGA-BH-A208-11A-51R-A156-13 | TCGA-BH-A208 |
| TCGA-BRCA | Solid Tissue Normal | TCGA-BH-A209-11A-42R-A156-13 | TCGA-BH-A209 |
| TCGA-BRCA | Solid Tissue Normal | TCGA-E2-A153-11A-31R-A12C-13 | TCGA-E2-A153 |
| TCGA-BRCA | Solid Tissue Normal | TCGA-E2-A158-11A-22R-A12C-13 | TCGA-E2-A158 |
| TCGA-BRCA | Solid Tissue Normal | TCGA-E2-A15I-11A-32R-A136-13 | TCGA-E2-A15I |
| TCGA-BRCA | Solid Tissue Normal | TCGA-E2-A15K-11A-13R-A12O-13 | TCGA-E2-A15K |
| TCGA-BRCA | Solid Tissue Normal | TCGA-E2-A15M-11A-22R-A12C-13 | TCGA-E2-A15M |
| TCGA-BRCA | Solid Tissue Normal | TCGA-E2-A1BC-11A-32R-A12O-13 | TCGA-E2-A1BC |
| TCGA-BRCA | Solid Tissue Normal | TCGA-E2-A1IG-11A-22R-A143-13 | TCGA-E2-A1IG |
| TCGA-BRCA | Solid Tissue Normal | TCGA-E2-A1L7-11A-33R-A143-13 | TCGA-E2-A1L7 |
| TCGA-BRCA | Solid Tissue Normal | TCGA-E2-A1LB-11A-22R-A143-13 | TCGA-E2-A1LB |
| TCGA-BRCA | Solid Tissue Normal | TCGA-E2-A1LH-11A-22R-A14C-13 | TCGA-E2-A1LH |
| TCGA-BRCA | Solid Tissue Normal | TCGA-E2-A1LS-11A-32R-A156-13 | TCGA-E2-A1LS |
| TCGA-BRCA | Solid Tissue Normal | TCGA-E9-A1N4-11A-33R-A14L-13 | TCGA-E9-A1N4 |
| TCGA-BRCA | Solid Tissue Normal | TCGA-E9-A1N5-11A-41R-A14C-13 | TCGA-E9-A1N5 |
| TCGA-BRCA | Solid Tissue Normal | TCGA-E9-A1N6-11A-32R-A143-13 | TCGA-E9-A1N6 |
| TCGA-BRCA | Solid Tissue Normal | TCGA-E9-A1N9-11A-71R-A14C-13 | TCGA-E9-A1N9 |
| TCGA-BRCA | Solid Tissue Normal | TCGA-E9-A1NA-11A-33R-A143-13 | TCGA-E9-A1NA |
| TCGA-BRCA | Solid Tissue Normal | TCGA-E9-A1ND-11A-43R-A143-13 | TCGA-E9-A1ND |
| TCGA-BRCA | Solid Tissue Normal | TCGA-E9-A1NF-11A-73R-A14C-13 | TCGA-E9-A1NF |
| TCGA-BRCA | Solid Tissue Normal | TCGA-E9-A1NG-11A-52R-A14L-13 | TCGA-E9-A1NG |
| TCGA-BRCA | Solid Tissue Normal | TCGA-E9-A1R7-11A-42R-A14L-13 | TCGA-E9-A1R7 |
| TCGA-BRCA | Solid Tissue Normal | TCGA-E9-A1RB-11A-33R-A156-13 | TCGA-E9-A1RB |
| TCGA-BRCA | Solid Tissue Normal | TCGA-E9-A1RC-11A-33R-A156-13 | TCGA-E9-A1RC |
| TCGA-BRCA | Solid Tissue Normal | TCGA-E9-A1RD-11A-33R-A156-13 | TCGA-E9-A1RD |
| TCGA-BRCA | Solid Tissue Normal | TCGA-E9-A1RF-11A-32R-A156-13 | TCGA-E9-A1RF |
| TCGA-BRCA | Solid Tissue Normal | TCGA-E9-A1RH-11A-34R-A168-13 | TCGA-E9-A1RH |
| TCGA-BRCA | Solid Tissue Normal | TCGA-E9-A1RI-11A-41R-A168-13 | TCGA-E9-A1RI |
| TCGA-BRCA | Solid Tissue Normal | TCGA-GI-A2C8-11A-22R-A16E-13 | TCGA-GI-A2C8 |
| TCGA-BRCA | Solid Tissue Normal | TCGA-GI-A2C9-11A-22R-A21U-13 | TCGA-GI-A2C9 |
| TCGA-UCEC | Solid Tissue Normal | TCGA-AJ-A2QL-11A-11R-A18L-13 | TCGA-AJ-A2QL |
| TCGA-UCEC | Solid Tissue Normal | TCGA-AJ-A3NC-11A-11R-A22I-13 | TCGA-AJ-A3NC |
| TCGA-UCEC | Solid Tissue Normal | TCGA-AJ-A3NE-11A-11R-A22I-13 | TCGA-AJ-A3NE |
| TCGA-UCEC | Solid Tissue Normal | TCGA-AJ-A3NH-11A-11R-A22I-13 | TCGA-AJ-A3NH |
| TCGA-UCEC | Solid Tissue Normal | TCGA-AX-A05Y-11A-11R-A27R-13 | TCGA-AX-A05Y |

| | | | |
|---|---|---|---|
| TCGA-UCEC | Solid Tissue Normal | TCGA-AX-A0IZ-11A-11R-A27R-13 | TCGA-AX-A0IZ |
| TCGA-UCEC | Solid Tissue Normal | TCGA-AX-A0J0-11A-11R-A27R-13 | TCGA-AX-A0J0 |
| TCGA-UCEC | Solid Tissue Normal | TCGA-AX-A1CF-11A-11R-A136-13 | TCGA-AX-A1CF |
| TCGA-UCEC | Solid Tissue Normal | TCGA-AX-A1CI-11A-11R-A136-13 | TCGA-AX-A1CI |
| TCGA-UCEC | Solid Tissue Normal | TCGA-AX-A1CK-11A-11R-A136-13 | TCGA-AX-A1CK |
| TCGA-UCEC | Solid Tissue Normal | TCGA-AX-A2H8-11A-11R-A17A-13 | TCGA-AX-A2H8 |
| TCGA-UCEC | Solid Tissue Normal | TCGA-AX-A2HA-11A-11R-A18L-13 | TCGA-AX-A2HA |
| TCGA-UCEC | Solid Tissue Normal | TCGA-AX-A2HC-11A-11R-A17A-13 | TCGA-AX-A2HC |
| TCGA-UCEC | Solid Tissue Normal | TCGA-AX-A2HD-11A-11R-A17A-13 | TCGA-AX-A2HD |
| TCGA-UCEC | Solid Tissue Normal | TCGA-BG-A2AD-11A-11R-A16E-13 | TCGA-BG-A2AD |
| TCGA-UCEC | Solid Tissue Normal | TCGA-BG-A3EW-11A-22R-A22I-13 | TCGA-BG-A3EW |
| TCGA-UCEC | Solid Tissue Normal | TCGA-BG-A3PP-11A-11R-A22I-13 | TCGA-BG-A3PP |
| TCGA-UCEC | Solid Tissue Normal | TCGA-BK-A0CB-11A-33R-A103-13 | TCGA-BK-A0CB |
| TCGA-UCEC | Solid Tissue Normal | TCGA-BK-A4ZD-11A-12R-A27R-13 | TCGA-BK-A4ZD |
| TCGA-UCEC | Solid Tissue Normal | TCGA-DI-A2QU-11A-11R-A18L-13 | TCGA-DI-A2QU |
| TCGA-UCEC | Solid Tissue Normal | TCGA-DI-A2QY-11A-11R-A19V-13 | TCGA-DI-A2QY |
| TCGA-UCEC | Solid Tissue Normal | TCGA-E6-A1M0-11A-11R-A143-13 | TCGA-E6-A1M0 |
| TCGA-UCEC | Solid Tissue Normal | TCGA-FL-A1YF-11A-12R-A16E-13 | TCGA-FL-A1YF |
| TCGA-UCEC | Solid Tissue Normal | TCGA-FL-A1YG-11A-12R-A16E-13 | TCGA-FL-A1YG |
| TCGA-UCEC | Solid Tissue Normal | TCGA-FL-A1YH-11A-11R-A16E-13 | TCGA-FL-A1YH |
| TCGA-UCEC | Solid Tissue Normal | TCGA-FL-A1YI-11A-11R-A16E-13 | TCGA-FL-A1YI |
| TCGA-UCEC | Solid Tissue Normal | TCGA-FL-A1YL-11A-11R-A16E-13 | TCGA-FL-A1YL |
| TCGA-UCEC | Solid Tissue Normal | TCGA-FL-A1YM-11A-12R-A17A-13 | TCGA-FL-A1YM |
| TCGA-UCEC | Solid Tissue Normal | TCGA-FL-A1YN-11A-11R-A17A-13 | TCGA-FL-A1YN |
| TCGA-UCEC | Solid Tissue Normal | TCGA-FL-A1YQ-11A-11R-A17A-13 | TCGA-FL-A1YQ |
| TCGA-UCEC | Solid Tissue Normal | TCGA-FL-A1YT-11A-12R-A17A-13 | TCGA-FL-A1YT |
| TCGA-UCEC | Solid Tissue Normal | TCGA-FL-A1YU-11A-11R-A17A-13 | TCGA-FL-A1YU |
| TCGA-UCEC | Solid Tissue Normal | TCGA-FL-A3WE-11A-11R-A22I-13 | TCGA-FL-A3WE |

**Table S2:** Machine learning 205 ranked features

| Rank model | miRNA ID | Importance weighting |
|---|---|---|
| **1** | hsa-mir-183 | 100 |
| **2** | hsa-mir-139 | 93.87 |
| **3** | hsa-mir-145 | 89.75 |
| **4** | hsa-mir-10b | 85.99 |
| **5** | hsa-mir-337 | 84.56 |
| **6** | hsa-mir-200c | 83.26 |
| **7** | hsa-mir-200a | 82.17 |
| **8** | hsa-mir-100 | 81.09 |
| **9** | hsa-mir-1247 | 80.4 |
| **10** | hsa-mir-195 | 78.89 |
| **11** | hsa-mir-379 | 77.99 |
| **12** | hsa-mir-1301 | 77.47 |
| **13** | hsa-mir-210 | 76.05 |
| **14** | hsa-mir-200b | 75.21 |
| **15** | hsa-mir-381 | 75.13 |
| **16** | hsa-mir-143 | 71.74 |
| **17** | hsa-mir-1307 | 71.55 |
| **18** | hsa-mir-130b | 70.99 |
| **19** | hsa-mir-144 | 70.38 |
| **20** | hsa-mir-182 | 67.92 |
| **21** | hsa-mir-204 | 64.77 |
| **22** | hsa-mir-15b | 63.56 |
| **23** | hsa-mir-126 | 63.54 |
| **24** | hsa-mir-3200 | 62.23 |
| **25** | hsa-mir-154 | 60.62 |
| **26** | hsa-mir-140 | 60.14 |
| **27** | hsa-mir-3127 | 59.62 |
| **28** | hsa-mir-191 | 58.79 |
| **29** | hsa-mir-3615 | 57.73 |
| **30** | hsa-mir-125b-1 | 57.64 |
| **31** | hsa-mir-148b | 57.11 |
| **32** | hsa-mir-335 | 57.04 |
| **33** | hsa-mir-125b-2 | 56.99 |
| **34** | hsa-mir-141 | 55.47 |
| **35** | hsa-let-7c | 55.29 |
| **36** | hsa-mir-3074 | 54.75 |
| **37** | hsa-mir-345 | 54.57 |
| **38** | hsa-mir-1266 | 53.11 |
| **39** | hsa-mir-3614 | 53.08 |
| **40** | hsa-mir-331 | 52.59 |
| **41** | hsa-mir-1226 | 51.63 |
| **42** | hsa-mir-369 | 51.54 |
| **43** | hsa-mir-134 | 50.36 |
| **44** | hsa-mir-155 | 50.25 |
| **45** | hsa-mir-299 | 49.71 |
| **46** | hsa-mir-152 | 49.22 |
| **47** | hsa-mir-1976 | 49.08 |
| **48** | hsa-mir-18a | 48.79 |
| **49** | hsa-mir-218-2 | 48.7 |
| **50** | hsa-mir-378c | 48.16 |
| **51** | hsa-mir-101-2 | 47.74 |
| **52** | hsa-mir-101-1 | 47.59 |
| **53** | hsa-mir-218-1 | 47.47 |
| **54** | hsa-mir-199b | 46.53 |
| **55** | hsa-mir-3170 | 46.3 |
| **56** | hsa-mir-1271 | 45.96 |
| **57** | hsa-mir-181b-1 | 45.65 |

| | | |
|---|---|---|
| **58** | hsa-mir-199a-1 | 45.39 |
| **59** | hsa-mir-136 | 45.19 |
| **60** | hsa-mir-26a-1 | 44.99 |
| **61** | hsa-mir-199a-2 | 44.99 |
| **62** | hsa-mir-29c | 44.98 |
| **63** | hsa-mir-30a | 44.93 |
| **64** | hsa-mir-26a-2 | 44.93 |
| **65** | hsa-mir-324 | 43.95 |
| **66** | hsa-mir-28 | 43.59 |
| **67** | hsa-mir-1180 | 43.52 |
| **68** | hsa-mir-181b-2 | 43.14 |
| **69** | hsa-mir-128-2 | 43.09 |
| **70** | hsa-mir-128-1 | 43.03 |
| **71** | hsa-mir-106b | 43 |
| **72** | hsa-mir-1248 | 42.15 |
| **73** | hsa-mir-377 | 41.58 |
| **74** | hsa-mir-33b | 41.43 |
| **75** | hsa-mir-203a | 41.37 |
| **76** | hsa-mir-22 | 41.32 |
| **77** | hsa-mir-187 | 40.99 |
| **78** | hsa-mir-374b | 40.09 |
| **79** | hsa-mir-217 | 39.86 |
| **80** | hsa-mir-365b | 39.75 |
| **81** | hsa-mir-365a | 39.67 |
| **82** | hsa-mir-382 | 39.51 |
| **83** | hsa-mir-190a | 39.39 |
| **84** | hsa-mir-2355 | 38.07 |
| **85** | hsa-mir-130a | 38.02 |
| **86** | hsa-mir-29a | 37.41 |
| **87** | hsa-mir-30b | 37.33 |
| **88** | hsa-mir-151b | 37.11 |
| **89** | hsa-mir-3065 | 37.03 |
| **90** | hsa-mir-224 | 36.84 |
| **91** | hsa-mir-184 | 36.74 |
| **92** | hsa-mir-3917 | 36.36 |
| **93** | hsa-mir-330 | 35.29 |
| **94** | hsa-mir-107 | 35.27 |
| **95** | hsa-mir-342 | 35.16 |
| **96** | hsa-mir-219a-1 | 34.69 |
| **97** | hsa-let-7i | 34.14 |
| **98** | hsa-mir-1306 | 34.06 |
| **99** | hsa-mir-370 | 33.8 |
| **100** | hsa-mir-203b | 33.57 |
| **101** | hsa-mir-127 | 33.2 |
| **102** | hsa-mir-30e | 33.02 |
| **103** | hsa-mir-1468 | 32.93 |
| **104** | hsa-mir-223 | 32.63 |
| **105** | hsa-mir-3677 | 32.59 |
| **106** | hsa-mir-320b-2 | 32.16 |
| **107** | hsa-mir-21 | 32.12 |
| **108** | hsa-mir-375 | 31.5 |
| **109** | hsa-mir-181a-1 | 31.39 |
| **110** | hsa-mir-26b | 31.14 |
| **111** | hsa-let-7d | 30.78 |
| **112** | hsa-mir-149 | 30.61 |
| **113** | hsa-mir-146b | 29.65 |
| **114** | hsa-mir-326 | 29.11 |
| **115** | hsa-mir-188 | 28.77 |
| **116** | hsa-mir-181c | 27.98 |

| | | |
|---|---|---|
| **117** | hsa-mir-338 | 27.88 |
| **118** | hsa-mir-181a-2 | 27.36 |
| **119** | hsa-mir-106a | 26.83 |
| **120** | hsa-mir-103a-2 | 26.25 |
| **121** | hsa-mir-103a-1 | 26.17 |
| **122** | hsa-mir-320b-1 | 25.94 |
| **123** | hsa-mir-2110 | 25.76 |
| **124** | hsa-mir-32 | 25.72 |
| **125** | hsa-mir-150 | 25.66 |
| **126** | hsa-mir-10a | 25.65 |
| **127** | hsa-mir-192 | 25.08 |
| **128** | hsa-mir-378a | 24.87 |
| **129** | hsa-mir-1270 | 24.3 |
| **130** | hsa-let-7f-1 | 24.18 |
| **131** | hsa-mir-197 | 24.13 |
| **132** | hsa-let-7f-2 | 23.82 |
| **133** | hsa-mir-135b | 23.78 |
| **134** | hsa-mir-181d | 23.04 |
| **135** | hsa-mir-374a | 22.77 |
| **136** | hsa-mir-186 | 22.75 |
| **137** | hsa-mir-185 | 22.71 |
| **138** | hsa-mir-142 | 22.69 |
| **139** | hsa-mir-3613 | 22.57 |
| **140** | hsa-mir-339 | 21.55 |
| **141** | hsa-mir-301a | 21.2 |
| **142** | hsa-mir-20b | 20.78 |
| **143** | hsa-mir-17 | 20.74 |
| **144** | hsa-let-7e | 19.75 |
| **145** | hsa-mir-3605 | 19.71 |
| **146** | hsa-mir-193b | 19.4 |
| **147** | hsa-mir-33a | 18.69 |
| **148** | hsa-mir-363 | 18.5 |
| **149** | hsa-mir-29b-2 | 17.78 |
| **150** | hsa-mir-29b-1 | 17.4 |
| **151** | hsa-mir-323a | 16.94 |
| **152** | hsa-mir-323b | 16.72 |
| **153** | hsa-mir-296 | 16.6 |
| **154** | hsa-mir-193a | 16.39 |
| **155** | hsa-let-7b | 16.35 |
| **156** | hsa-mir-205 | 15.28 |
| **157** | hsa-mir-25 | 15.11 |
| **158** | hsa-mir-19b-2 | 14.38 |
| **159** | hsa-mir-196b | 13.9 |
| **160** | hsa-mir-15a | 13.84 |
| **161** | hsa-mir-151a | 13.22 |
| **162** | hsa-mir-328 | 13.21 |
| **163** | hsa-mir-16-2 | 12.96 |
| **164** | hsa-mir-19b-1 | 12.94 |
| **165** | hsa-mir-214 | 12.8 |
| **166** | hsa-mir-16-1 | 12.28 |
| **167** | hsa-mir-23b | 11.02 |
| **168** | hsa-mir-125a | 10.71 |
| **169** | hsa-mir-3653 | 10.33 |
| **170** | hsa-mir-340 | 10.32 |
| **171** | hsa-mir-30c-2 | 9.53 |
| **172** | hsa-mir-132 | 9.34 |
| **173** | hsa-mir-30c-1 | 9.29 |
| **174** | hsa-mir-3130-1 | 8.91 |
| **175** | hsa-mir-221 | 8.38 |

| | | |
|---|---|---|
| **176** | hsa-mir-194-2 | 8.11 |
| **177** | hsa-let-7a-2 | 7.96 |
| **178** | hsa-mir-222 | 7.9 |
| **179** | hsa-let-7a-1 | 7.81 |
| **180** | hsa-mir-146a | 7.77 |
| **181** | hsa-let-7a-3 | 7.75 |
| **182** | hsa-mir-3130-2 | 7.19 |
| **183** | hsa-mir-1249 | 7.09 |
| **184** | hsa-mir-23a | 6.9 |
| **185** | hsa-mir-34a | 6.75 |
| **186** | hsa-mir-212 | 6.12 |
| **187** | hsa-mir-20a | 5.14 |
| **188** | hsa-mir-196a-1 | 5.14 |
| **189** | hsa-mir-27a | 4.64 |
| **190** | hsa-mir-361 | 3.88 |
| **191** | hsa-let-7g | 3.62 |
| **192** | hsa-mir-320a | 3.45 |
| **193** | hsa-mir-30d | 3.29 |
| **194** | hsa-mir-24-2 | 2.81 |
| **195** | hsa-mir-31 | 2.75 |
| **196** | hsa-mir-24-1 | 2.36 |
| **197** | hsa-mir-194-1 | 2.3 |
| **198** | hsa-mir-27b | 2.1 |
| **199** | hsa-mir-34c | 1.63 |
| **200** | hsa-mir-34b | 1.33 |
| **201** | hsa-mir-3607 | 1.17 |
| **202** | hsa-mir-148a | 0.6 |
| **203** | hsa-mir-362 | 0.45 |
| **204** | hsa-mir-1287 | 0.43 |
| **205** | hsa-mir-1296 | 0.11 |

**Table S3:** Comparison of SVM with other learning methods

| Methods | n° of Features | Accuracy | AUC | Sensitivity | Specificity | F1 score |
|---|---|---|---|---|---|---|
| SVM[a] | 42 | 0.983 | 0.931 | 0.85 | 0.992 | 0.9 |
| Logistic Regression[a] | 42 | 0.98 | 0.911 | 0.89 | 0.98 | 0.87 |
| Boosted Logistic Regression[a] | 42 | 0.982 | 0.918 | 0.89 | 0.98 | 0.87 |
| Regression with LASSO penalty[a] | 42 | 0.975 | 0.92 | 0.83 | 0.984 | 0.85 |
| Elastic Net[a] | 42 | 0.979 | 0.922 | 0.85 | 0.987 | 0.87 |
| Random Forest | 42 | 0.99 | 0.97 | 0.92 | 0.99 | 0.93 |
| avNNet | 42 | 0.98 | 0.93 | 0.97 | 0.99 | 0.91 |
| nnet | 42 | 0.98 | 0.93 | 0.92 | 0.99 | 0.9 |

[a] Intersection of all feat. selected (205) with ERBB target (28 + 15 top importance, 1 in common)

**Table S4:** miRTarBase, 158 miRNAs targeting ERBB isoforms

| miRTarBase ID | miRNA | Target Gene | Experiments | Support Type | References (PMID) |
|---|---|---|---|---|---|
| MIRT000036 | hsa-miR-559 | ERBB2 | Luciferase reporter assay//Reporter assay;Other | Functional MTI | 19486885 |
| MIRT000037 | hsa-miR-548d-3p | ERBB2 | Luciferase reporter assay//Reporter assay;Other | Functional MTI | 19486885 |
| MIRT002938 | hsa-miR-125b-5p | ERBB3 | Luciferase reporter assay//Northern blot//qRT-PCR//Western blot | Functional MTI | 17110380 |
| MIRT002938 | hsa-miR-125b-5p | ERBB3 | Immunoblot//Immunohistochemistry//Luciferase reporter assay//qRT-PCR | Functional MTI | 23146892 |
| MIRT002938 | hsa-miR-125b-5p | ERBB3 | Western blot//qRT-PCR | Functional MTI | 23519125 |
| MIRT002939 | hsa-miR-125b-5p | ERBB2 | Western blot | Functional MTI | 19825990 |
| MIRT002939 | hsa-miR-125b-5p | ERBB2 | Luciferase reporter assay//Northern blot//qRT-PCR//Western blot | Functional MTI | 17110380 |
| MIRT002939 | hsa-miR-125b-5p | ERBB2 | Luciferase reporter assay//Microarray//Western blot | Functional MTI | 20864407 |
| MIRT002939 | hsa-miR-125b-5p | ERBB2 | Luciferase reporter assay//Western blot | Functional MTI | 22460089 |
| MIRT002939 | hsa-miR-125b-5p | ERBB2 | qRT-PCR//Luciferase reporter assay//Immunoblot//Western blot | Functional MTI | 25388283 |
| MIRT002939 | hsa-miR-125b-5p | ERBB2 | Luciferase reporter assay//Western blot | Functional MTI | 26966351 |
| MIRT002939 | hsa-miR-125b-5p | ERBB2 | Luciferase reporter assay | Functional MTI | 26544868 |
| MIRT002939 | hsa-miR-125b-5p | ERBB2 | Luciferase reporter assay//qRT-PCR//Western blot | Functional MTI | 25833836 |
| MIRT003532 | hsa-miR-372-3p | ERBB4 | Luciferase reporter assay//Microarray | Functional MTI | 19885849 |
| MIRT003533 | hsa-miR-19a-3p | ERBB4 | Luciferase reporter assay//Microarray | Functional MTI | 19885849 |
| MIRT003534 | hsa-miR-302d-3p | ERBB4 | Luciferase reporter assay//Microarray | Functional MTI | 19885849 |
| MIRT003659 | hsa-miR-205-5p | ERBB3 | Luciferase reporter assay//Reporter assay;Other | Functional MTI | 19238171 |
| MIRT003659 | hsa-miR-205-5p | ERBB3 | Luciferase reporter assay | Functional MTI | 20065103 |
| MIRT003659 | hsa-miR-205-5p | ERBB3 | Luciferase reporter assay//qRT-PCR//Western blot | Functional MTI | 19276373 |
| MIRT003659 | hsa-miR-205-5p | ERBB3 | Luciferase reporter assay//Microarray//Western blot | Functional MTI | 20864407 |
| MIRT003659 | hsa-miR-205-5p | ERBB3 | Western blot//qRT-PCR | Functional MTI | 23519125 |
| MIRT004318 | hsa-miR-21-5p | ERBB2 | qRT-PCR//Western blot | Functional MTI | 19419954 |
| MIRT005117 | hsa-miR-125a-5p | ERBB3 | Luciferase reporter assay//Northern blot//qRT-PCR//Western blot | Functional MTI | 17110380 |
| MIRT005117 | hsa-miR-125a-5p | ERBB3 | Luciferase reporter assay//Microarray//Western blot | Functional MTI | 20864407 |
| MIRT005117 | hsa-miR-125a-5p | ERBB3 | Western blot//qRT-PCR | Functional MTI | 23519125 |
| MIRT005118 | hsa-miR-125a-5p | ERBB2 | Luciferase reporter assay//Northern blot//qRT-PCR//Western blot | Functional MTI | 17110380 |
| MIRT005118 | hsa-miR-125a-5p | ERBB2 | Luciferase reporter assay | Functional MTI | 21220473 |
| MIRT005118 | hsa-miR-125a-5p | ERBB2 | Western blot//qRT-PCR | Functional MTI | 23519125 |
| MIRT005118 | hsa-miR-125a-5p | ERBB2 | Luciferase reporter assay//qRT-PCR//Western blot | Functional MTI | 25833836 |
| MIRT005805 | hsa-miR-331-3p | ERBB2 | Luciferase reporter assay//Microarray//Western blot | Functional MTI | 20864407 |
| MIRT005805 | hsa-miR-331-3p | ERBB2 | low//Immunohistochemistry//Luciferase reporter assay//qRT-PCR//Western blo | Functional MTI | 24775712 |
| MIRT005805 | hsa-miR-331-3p | ERBB2 | Luciferase reporter assay | Functional MTI | 24148764 |
| MIRT005805 | hsa-miR-331-3p | ERBB2 | Luciferase reporter assay//Western blot | Functional MTI | 26718987 |
| MIRT005806 | hsa-miR-146a-5p | ERBB4 | Luciferase reporter assay//Microarray//Western blot | Functional MTI | 20864407 |
| MIRT005806 | hsa-miR-146a-5p | ERBB4 | Luciferase reporter assay//Northern blot | Functional MTI | 23619365 |
| MIRT006210 | hsa-miR-22-3p | ERBB3 | Luciferase reporter assay//qRT-PCR//Western blot | Functional MTI | 22484852 |
| MIRT006982 | hsa-miR-205-5p | ERBB2 | qRT-PCR//Western blot | Functional MTI | 21787752 |

| | | | | | |
|---|---|---|---|---|---|
| MIRT006982 | hsa-miR-205-5p | ERBB2 | Luciferase reporter assay//qRT-PCR//Western blot | Functional MTI | 26181203 |
| MIRT007152 | hsa-miR-199b-5p | ERBB2 | Luciferase reporter assay//qRT-PCR//Western blot | Functional MTI | 23296799 |
| MIRT007265 | hsa-miR-199a-5p | ERBB2 | Western blot | Functional MTI | 23437196 |
| MIRT007265 | hsa-miR-199a-5p | ERBB2 | Immunoblot//Immunohistochemistry//Luciferase reporter assay//qRT-PCR | Functional MTI | 23146892 |
| MIRT023179 | hsa-miR-124-3p | ERBB2 | Microarray | Functional MTI (Weak) | 18668037 |
| MIRT030589 | hsa-miR-24-3p | ERBB3 | Microarray | Functional MTI (Weak) | 19748357 |
| MIRT030589 | hsa-miR-24-3p | ERBB3 | HITS-CLIP | Functional MTI (Weak) | 23824327 |
| MIRT043637 | hsa-miR-326 | ERBB2 | CLASH | Functional MTI (Weak) | 23622248 |
| MIRT053607 | hsa-miR-199a-5p | ERBB3 | Immunoblot//Immunohistochemistry//Luciferase reporter assay//qRT-PCR | Functional MTI | 23146892 |
| MIRT053647 | hsa-miR-143-3p | ERBB3 | Microarray | Functional MTI (Weak) | 22942087 |
| MIRT053661 | hsa-miR-145-5p | ERBB4 | Microarray | Functional MTI (Weak) | 22942087 |
| MIRT053704 | hsa-miR-221-3p | ERBB4 | Microarray | Functional MTI (Weak) | 22942087 |
| MIRT053780 | hsa-miR-375 | ERBB2 | Luciferase reporter assay//qRT-PCR//Western blot | Functional MTI | 24926380 |
| MIRT053780 | hsa-miR-375 | ERBB2 | HITS-CLIP | Functional MTI (Weak) | 24906430 |
| MIRT053780 | hsa-miR-375 | ERBB2 | Flow//qRT-PCR//Western blot | Functional MTI | 26893657 |
| MIRT054130 | hsa-miR-25-3p | ERBB2 | Luciferase reporter assay//qRT-PCR//Western blot | Functional MTI | 25043310 |
| MIRT054800 | hsa-miR-302b-3p | ERBB4 | Luciferase reporter assay//qRT-PCR//Western blot | Functional MTI | 24438167 |
| MIRT065956 | hsa-miR-33a-3p | ERBB3 | PAR-CLIP | Functional MTI (Weak) | 22012620 |
| MIRT065961 | hsa-miR-592 | ERBB3 | PAR-CLIP | Functional MTI (Weak) | 22012620 |
| MIRT065963 | hsa-miR-2113 | ERBB3 | PAR-CLIP | Functional MTI (Weak) | 22012620 |
| MIRT065968 | hsa-miR-4698 | ERBB3 | PAR-CLIP | Functional MTI (Weak) | 22012620 |
| MIRT065971 | hsa-miR-6507-5p | ERBB3 | PAR-CLIP | Functional MTI (Weak) | 22012620 |
| MIRT076987 | hsa-miR-670-3p | ERBB2 | HITS-CLIP | Functional MTI (Weak) | 24906430 |
| MIRT076993 | hsa-miR-4326 | ERBB2 | HITS-CLIP | Functional MTI (Weak) | 24906430 |
| MIRT438501 | hsa-miR-552-3p | ERBB2 | Luciferase reporter assay | Functional MTI | 24148764 |
| MIRT438502 | hsa-miR-541-3p | ERBB2 | Luciferase reporter assay | Functional MTI | 24148764 |
| MIRT438503 | hsa-miR-498 | ERBB2 | Luciferase reporter assay | Functional MTI | 24148764 |
| MIRT438504 | hsa-miR-323b-5p | ERBB2 | Luciferase reporter assay | Functional MTI | 24148764 |
| MIRT438505 | hsa-miR-193a-5p | ERBB2 | Luciferase reporter assay | Functional MTI | 24148764 |
| MIRT438505 | hsa-miR-193a-5p | ERBB2 | Luciferase reporter assay | Functional MTI | 27203740 |
| MIRT438506 | hsa-miR-193a-3p | ERBB2 | Luciferase reporter assay | Functional MTI | 24148764 |
| MIRT438507 | hsa-miR-134-5p | ERBB2 | Luciferase reporter assay | Functional MTI | 24148764 |
| MIRT537654 | hsa-miR-597-5p | ERBB3 | PAR-CLIP | Functional MTI (Weak) | 22012620 |
| MIRT537655 | hsa-miR-8063 | ERBB3 | PAR-CLIP | Functional MTI (Weak) | 22012620 |
| MIRT537656 | hsa-miR-7109-3p | ERBB3 | PAR-CLIP | Functional MTI (Weak) | 22012620 |
| MIRT537657 | hsa-miR-548e-5p | ERBB3 | PAR-CLIP | Functional MTI (Weak) | 22012620 |
| MIRT608163 | hsa-miR-6739-3p | ERBB2 | HITS-CLIP | Functional MTI (Weak) | 24906430 |
| MIRT608164 | hsa-miR-4677-5p | ERBB2 | HITS-CLIP | Functional MTI (Weak) | 24906430 |
| MIRT608165 | hsa-miR-7156-5p | ERBB2 | HITS-CLIP | Functional MTI (Weak) | 24906430 |

| | | | | | |
|---|---|---|---|---|---|
| MIRT608166 | hsa-miR-4273 | ERBB2 | HITS-CLIP | Functional MTI (Weak) | 24906430 |
| MIRT608167 | hsa-miR-7109-5p | ERBB2 | HITS-CLIP | Functional MTI (Weak) | 24906430 |
| MIRT608168 | hsa-miR-4790-5p | ERBB2 | HITS-CLIP | Functional MTI (Weak) | 24906430 |
| MIRT608169 | hsa-miR-6868-3p | ERBB2 | HITS-CLIP | Functional MTI (Weak) | 24906430 |
| MIRT608170 | hsa-miR-3127-5p | ERBB2 | HITS-CLIP | Functional MTI (Weak) | 24906430 |
| MIRT608171 | hsa-miR-22-5p | ERBB2 | HITS-CLIP | Functional MTI (Weak) | 24906430 |
| MIRT608172 | hsa-miR-2116-5p | ERBB2 | HITS-CLIP | Functional MTI (Weak) | 24906430 |
| MIRT608173 | hsa-miR-593-3p | ERBB2 | HITS-CLIP | Functional MTI (Weak) | 24906430 |
| MIRT608174 | hsa-miR-6885-5p | ERBB2 | HITS-CLIP | Functional MTI (Weak) | 24906430 |
| MIRT608175 | hsa-miR-328-5p | ERBB2 | HITS-CLIP | Functional MTI (Weak) | 24906430 |
| MIRT608176 | hsa-miR-6887-5p | ERBB2 | HITS-CLIP | Functional MTI (Weak) | 24906430 |
| MIRT608177 | hsa-miR-486-3p | ERBB2 | HITS-CLIP | Functional MTI (Weak) | 24906430 |
| MIRT608178 | hsa-miR-6795-5p | ERBB2 | HITS-CLIP | Functional MTI (Weak) | 24906430 |
| MIRT608179 | hsa-miR-3140-3p | ERBB2 | HITS-CLIP | Functional MTI (Weak) | 24906430 |
| MIRT608180 | hsa-miR-3116 | ERBB2 | HITS-CLIP | Functional MTI (Weak) | 24906430 |
| MIRT608181 | hsa-miR-1254 | ERBB2 | HITS-CLIP | Functional MTI (Weak) | 24906430 |
| MIRT608182 | hsa-miR-4790-3p | ERBB2 | HITS-CLIP | Functional MTI (Weak) | 24906430 |
| MIRT608183 | hsa-miR-6730-3p | ERBB2 | HITS-CLIP | Functional MTI (Weak) | 24906430 |
| MIRT608184 | hsa-miR-4680-3p | ERBB2 | HITS-CLIP | Functional MTI (Weak) | 24906430 |
| MIRT608185 | hsa-miR-5187-3p | ERBB2 | HITS-CLIP | Functional MTI (Weak) | 24906430 |
| MIRT608186 | hsa-miR-4427 | ERBB2 | HITS-CLIP | Functional MTI (Weak) | 24906430 |
| MIRT608187 | hsa-miR-4653-5p | ERBB2 | HITS-CLIP | Functional MTI (Weak) | 24906430 |
| MIRT608188 | hsa-miR-3921 | ERBB2 | HITS-CLIP | Functional MTI (Weak) | 24906430 |
| MIRT608189 | hsa-miR-301b-5p | ERBB2 | HITS-CLIP | Functional MTI (Weak) | 24906430 |
| MIRT608190 | hsa-miR-301a-5p | ERBB2 | HITS-CLIP | Functional MTI (Weak) | 24906430 |
| MIRT608191 | hsa-miR-4315 | ERBB2 | HITS-CLIP | Functional MTI (Weak) | 24906430 |
| MIRT608192 | hsa-miR-146a-3p | ERBB2 | HITS-CLIP | Functional MTI (Weak) | 24906430 |
| MIRT608193 | hsa-miR-3667-3p | ERBB2 | HITS-CLIP | Functional MTI (Weak) | 24906430 |
| MIRT608194 | hsa-miR-6734-3p | ERBB2 | HITS-CLIP | Functional MTI (Weak) | 24906430 |
| MIRT608195 | hsa-miR-6891-3p | ERBB2 | HITS-CLIP | Functional MTI (Weak) | 24906430 |
| MIRT608196 | hsa-miR-6072 | ERBB2 | HITS-CLIP | Functional MTI (Weak) | 24906430 |
| MIRT608197 | hsa-miR-6754-5p | ERBB2 | HITS-CLIP | Functional MTI (Weak) | 24906430 |
| MIRT608198 | hsa-miR-4441 | ERBB2 | HITS-CLIP | Functional MTI (Weak) | 24906430 |
| MIRT608199 | hsa-miR-4270 | ERBB2 | HITS-CLIP | Functional MTI (Weak) | 24906430 |
| MIRT608200 | hsa-miR-211-3p | ERBB2 | HITS-CLIP | Functional MTI (Weak) | 24906430 |
| MIRT608201 | hsa-miR-4748 | ERBB2 | HITS-CLIP | Functional MTI (Weak) | 24906430 |
| MIRT608202 | hsa-miR-329-5p | ERBB2 | HITS-CLIP | Functional MTI (Weak) | 24906430 |
| MIRT608203 | hsa-miR-4464 | ERBB2 | HITS-CLIP | Functional MTI (Weak) | 24906430 |
| MIRT608204 | hsa-miR-7845-5p | ERBB2 | HITS-CLIP | Functional MTI (Weak) | 24906430 |

| | | | | | |
|---|---|---|---|---|---|
| MIRT608205 | hsa-miR-888-3p | ERBB2 | HITS-CLIP | Functional MTI (Weak) | 24906430 |
| MIRT608206 | hsa-miR-4506 | ERBB2 | HITS-CLIP | Functional MTI (Weak) | 24906430 |
| MIRT615956 | hsa-miR-4446-5p | ERBB3 | HITS-CLIP | Functional MTI (Weak) | 23824327 |
| MIRT615956 | hsa-miR-4446-5p | ERBB3 | HITS-CLIP | Functional MTI (Weak) | 19536157 |
| MIRT615957 | hsa-miR-3679-3p | ERBB3 | HITS-CLIP | Functional MTI (Weak) | 23824327 |
| MIRT615957 | hsa-miR-3679-3p | ERBB3 | HITS-CLIP | Functional MTI (Weak) | 19536157 |
| MIRT615958 | hsa-miR-6826-3p | ERBB3 | HITS-CLIP | Functional MTI (Weak) | 23824327 |
| MIRT615958 | hsa-miR-6826-3p | ERBB3 | HITS-CLIP | Functional MTI (Weak) | 19536157 |
| MIRT615959 | hsa-miR-4732-5p | ERBB3 | HITS-CLIP | Functional MTI (Weak) | 23824327 |
| MIRT615959 | hsa-miR-4732-5p | ERBB3 | HITS-CLIP | Functional MTI (Weak) | 19536157 |
| MIRT615960 | hsa-miR-6832-5p | ERBB3 | HITS-CLIP | Functional MTI (Weak) | 23824327 |
| MIRT615960 | hsa-miR-6832-5p | ERBB3 | HITS-CLIP | Functional MTI (Weak) | 19536157 |
| MIRT615961 | hsa-miR-331-3p | ERBB3 | HITS-CLIP | Functional MTI (Weak) | 23824327 |
| MIRT615961 | hsa-miR-331-3p | ERBB3 | HITS-CLIP | Functional MTI (Weak) | 19536157 |
| MIRT615962 | hsa-miR-6810-3p | ERBB3 | HITS-CLIP | Functional MTI (Weak) | 23824327 |
| MIRT615962 | hsa-miR-6810-3p | ERBB3 | HITS-CLIP | Functional MTI (Weak) | 19536157 |
| MIRT615963 | hsa-miR-6801-3p | ERBB3 | HITS-CLIP | Functional MTI (Weak) | 23824327 |
| MIRT615963 | hsa-miR-6801-3p | ERBB3 | HITS-CLIP | Functional MTI (Weak) | 19536157 |
| MIRT630919 | hsa-miR-4328 | ERBB3 | HITS-CLIP | Functional MTI (Weak) | 23824327 |
| MIRT719394 | hsa-miR-301a-5p | ERBB3 | HITS-CLIP | Functional MTI (Weak) | 19536157 |
| MIRT719395 | hsa-miR-5701 | ERBB3 | HITS-CLIP | Functional MTI (Weak) | 19536157 |
| MIRT719396 | hsa-miR-323b-3p | ERBB3 | HITS-CLIP | Functional MTI (Weak) | 19536157 |
| MIRT732771 | hsa-miR-34a-5p | ERBB2 | Luciferase reporter assay//Western blot | Functional MTI | 27813227 |
| MIRT733558 | hsa-miR-155-3p | ERBB2 | Luciferase reporter assay//qRT-PCR//Western blot | Functional MTI | 27065318 |
| MIRT733635 | hsa-miR-130b-3p | ERBB2 | Luciferase reporter assay | Functional MTI | 26316103 |
| MIRT733746 | hsa-miR-1296-5p | ERBB2 | Luciferase reporter assay//qRT-PCR//Western blot | Functional MTI | 28099468 |
| MIRT733749 | hsa-miR-133a-3p | ERBB2 | Luciferase reporter assay//qRT-PCR//Western blot | Functional MTI | 28109082 |
| MIRT733913 | hsa-miR-219a-2-3p | ERBB3 | qRT-PCR | Functional MTI (Weak) | 26043902 |
| MIRT734059 | hsa-miR-193a-3p | ERBB4 | Luciferase reporter assay//qRT-PCR//Western blot | Functional MTI | 24469061 |
| MIRT734069 | hsa-miR-3622b-5p | ERBB2 | Luciferase reporter assay//qRT-PCR//Western blot | Functional MTI | 28160563 |
| MIRT734579 | hsa-miR-22-3p | ERBB2 | Luciferase reporter assay | Functional MTI | 26544868 |
| MIRT734641 | hsa-miR-143-5p | ERBB3 | Luciferase reporter assay//qRT-PCR//Western blot | Functional MTI | 25248370 |
| MIRT735021 | hsa-miR-146b-5p | ERBB4 | Luciferase reporter assay | Functional MTI | 27400799 |
| MIRT783296 | hsa-miR-1273f | ERBB3 | HITS-CLIP | Functional MTI (Weak) | 27418678 |
| MIRT783358 | hsa-miR-1276 | ERBB3 | HITS-CLIP | Functional MTI (Weak) | 27418678 |
| MIRT785707 | hsa-miR-4311 | ERBB3 | HITS-CLIP | Functional MTI (Weak) | 27418678 |
| MIRT787937 | hsa-miR-583 | ERBB3 | HITS-CLIP | Functional MTI (Weak) | 27418678 |
| MIRT788557 | hsa-miR-6751-5p | ERBB3 | HITS-CLIP | Functional MTI (Weak) | 27418678 |
| MIRT788561 | hsa-miR-6752-5p | ERBB3 | HITS-CLIP | Functional MTI (Weak) | 27418678 |

| | | | | | |
|---|---|---|---|---|---|
| MIRT788821 | hsa-miR-6803-5p | ERBB3 | HITS-CLIP | Functional MTI (Weak) | 27418678 |
| MIRT789021 | hsa-miR-6835-5p | ERBB3 | HITS-CLIP | Functional MTI (Weak) | 27418678 |
| MIRT789043 | hsa-miR-6842-5p | ERBB3 | HITS-CLIP | Functional MTI (Weak) | 27418678 |
| MIRT789412 | hsa-miR-7110-5p | ERBB3 | HITS-CLIP | Functional MTI (Weak) | 27418678 |

**Table S5:** Model Performance different combinations

| Model candidates | n° of Features | Accuracy | AUC | Sensitivity | Specificity |
|---|---|---|---|---|---|
| All features from Feature Selection | 205 | 0.968 | 0.926 | 0.55 | 0.995 |
| Only ERBBA target (158) available in TCGA dataset | 32 | 0.983 | 0.91 | 0.9 | 0.98 |
| Intersection of all feat. selected (205) with ERBB target (158) | 28 | 0.977 | 0.88 | 0.875 | 0.984 |
| Intersection of all feat. selected (205) with ERBB target (28 + 10 top importance, 1 in common) | 37 | 0.982 | 0.912 | 0.875 | 0.988 |
| **Intersection of all feat. selected (205) with ERBB target (28 + 15 top importance, 1 in common)** | **42** | **0.983** | **0.931** | **0.85** | **0.992** |
| Intersection of all feat. selected (205) with ERBB target (28 + 15 top importance, 1 in common)[a] | 42 | 0.976 | 0.92 | 0.7 | 0.99 |
| Intersection of all feat. selected (205) with ERBB target (28 + 20 top importance, 3 in common) | 45 | 0.983 | 0.931 | 0.85 | 0.992 |
| Intersection of all feat. selected (205) with ERBB target (28 + 25 top importance, 3 in common) | 50 | 0.979 | 0.914 | 0.8 | 0.99 |
| Intersection of all feat. selected (205) with ERBB target (28 + 30 top importance, 5 in common) | 53 | 0.98 | 0.926 | 0.8 | 0.992 |
| Top 10 Importance | 10 | 0.962 | 0.8 | 1 | 0.96 |
| Top 15 importance | 15 | 0.97 | 0.835 | 0.975 | 0.969 |
| Top 20 importance | 20 | 0.971 | 0.843 | 0.95 | 0.972 |
| Top 25 importance | 25 | 0.973 | 0.853 | 0.925 | 0.976 |
| Top 30 importance | 30 | 0.976 | 0.867 | 0.925 | 0.979 |
| All features from Feature Selection | | | | | |

a WITHOUT ARTIFICIAL SAMPLES

**Table S6: Comprehensive analyses on multiple databases retrieved the predominantly expressed miRNA mature sequences for *in vitro* validations.** The Cancer Genome Atlas (TCGA) breast, ovarian and uterine corpus endometrial cancer, Cancer cell line Encyclopaedia (CCLE) and Human miRNA-Disease Database (HMDD) (v.3.0) annotations for 14 semantic entries associated with breast, ovarian and endometrial cancers.

| Accession | miRNA | miRNA isoform reported in databases | *In vitro* validated mature miRNA |
|---|---|---|---|
| MI0006382 | hsa-mir-1247 | not specified | hsa-miR-1247-5p<br>hsa-miR-1247-3p |
| MI0003815 | hsa-mir-1301 | TCGA | hsa-miR-1301-3p |
| MI0000789 | hsa-mir-381 | TCGA and HMDD | hsa-miR-381-3p |
| MI0000812 | hsa-mir-331 | not specified | hsa-miR-331-5p<br>hsa-miR-331-3p |
| MI0003129 | hsa-mir-146b | not specified | hsa-miR-146b-5p<br>hsa-miR-146b-3p |
| MI0000091 | hsa-mir-33a | TCGA and HMDD | hsa-miR-33a-3p |
| MI0000487 | hsa-mir-193a | not specified | hsa-miR-193a-5p<br>hsa-miR-193a-3p |
| MI0003780 | hsa-mir-1296 | TCGA | hsa-miR-1296-5p |
| MI0014206 | hsa-mir-323b | TCGA and CCLE | hsa-miR-323b-3p<br>hsa-miR-323a-3p |
